# The descriptive epidemiology of brand-specific gun ownership in the US: results from the 2019 National Lawful Use of Guns Survey

**DOI:** 10.1186/s40621-021-00305-1

**Published:** 2021-03-22

**Authors:** Michael Siegel, Devon Dunn, Faizah Shareef, Miriam Neufeld, Claire Boine

**Affiliations:** Department of Community Health Sciences, Boston University School of Public Health, 801 Massachusetts Avenue, CT4, Boston, MA 02118 USA

**Keywords:** Firearms, Firearm laws, Firearm violence, Gun policy, Guns

## Abstract

**Background:**

No previous study has identified the specific brands of guns owned by gun owners. This study aimed to: (1) ascertain and describe patterns of brand- and model-specific gun ownership among US gun owners; and (2) investigate the relationship between gun owners’ brand and model preferences and their attitudes towards common firearm violence prevention policies.

**Methods:**

Using a national, pre-recruited internet panel of US adults in 2019, we surveyed gun owners (*N* = 2086) to ascertain their opinions regarding firearm violence prevention policies and to assess the brands and models of guns that they owned.

**Results:**

Brand-specific gun ownership was highly concentrated and was dominated by three pistol brands, two revolver brands, three rifle brands, and three shotgun brands. There was wide variation in policy attitudes among owners of different gun brands, but little variation across owners of different gun types (i.e., pistols, rifles, revolvers, shotguns). We were able to identify the specific gun models owned by 1218 (59.4%) of the gun owners. Based on the classification of these gun models into three types we categorized the gun ownership pattern of the sample as 33.4% recreational, 45.5% self-defense, and 21.1% tactical. There were marked differences in support for firearm-related policies among the three groups, with support generally highest among the Recreation group and lowest among the Tactical group.

**Conclusion:**

We conclude that gun brands and models are strong predictors of a gun owner’s attitudes regarding firearm-related policies. This information could help public health practitioners develop segment-specific communications that will appeal to each group in order to more effectively engage gun owners in firearm violence prevention.

**Supplementary Information:**

The online version contains supplementary material available at 10.1186/s40621-021-00305-1.

## Background

Nearly 40,000 people in the United States die each year from firearm violence (Centers for Disease Control and Prevention (CDC), 2020). In recent years, the problem of firearm violence has escalated into an unprecedentedly salient public policy issue marked by fierce and passionate debate. For example, in 2019, firearm violence prevention policy was, for the first time, a central focus of the Democratic presidential candidate debates (Talbot, 2019). That same year, the House of Representatives considered legislation to ban military-style weapons and high capacity ammunition magazines and advanced legislation to require universal background checks before gun purchase, although the bill has stalled in the Senate.

It is widely perceived that there is a polarizing split between firearm violence prevention advocates and gun owners (Joslyn et al., 2017) that contributes to the paucity of legislative action (Metzl, 2019). The truth, however, is more nuanced. Gun owners overwhelmingly support policies such as universal background checks that are intended to keep guns out of the hands of people who are at high risk for violence (Quinnipiac University, 2019), but are lukewarm toward policies that would prohibit certain types of firearms or ammunition or that are perceived as undermining their own ability to protect themselves (Siegel and Boine, 2020). Thus, the gap may not be as insurmountable as it may seem. To bridge this gap, public health practitioners need to develop a better understanding of gun owners (Crifasi et al., 2018; Metzl, 2019) though, as Yamane points out, there is an enormity of firearm crime research but outside of the military and law enforcement fields, scant research on the lawful uses of guns (Yamane, 2017, 2018). In particular, there are gaps in our understanding of the relationship between gun owner identity and political opinion. We do know, for example, that gun owners are more likely to be Republican and to see gun ownership as signaling a set of conservative values, such as rural living, individual autonomy, and limited governmental reach (Horwitz & Anderson, 2009). However, little is known about differences *among* gun owners; that is, how do differences in gun identity among gun owners relate to their political opinions, especially those relating to firearm prevention policies? Similarly, there are numerous studies that have examined policy opinions among gun owners generally (Barry et al., 2015, 2018, 2019; Crifasi et al., 2020), but few, if any, have explored differences *among* gun owners in their opinions regarding gun policies and how these differences may relate to their gun-related identity.

Marketing theory suggests that one way to understand gun owner identity is to examine the individual brand preferences of gun owners. Consumer brand preferences can provide essential clues regarding an individual’s beliefs and identity (Black & Veloutsou, 2017; Ilaw, 2014). Avery and Keinan (2015) posit that a brand is an individual and collective frame that “enables a consumer to identify, classify, interpret, and utilize the meaning of an associated product or service in a way that differs from other similar products or services” (p. 210). Through advertising and other marketing communication, such as logos and taglines, brand managers create a narrative. Many consumers utilize such narratives and view certain brands as an extension of their self-image (Avery and Keinan, 2015). Brands can be used by the individual to tell others about their lifestyle and beliefs, including their political beliefs (Evans & Hastings, 2008). The central premise behind this study is that gun brand identities may signal different sociopolitical identities among gun owners which may be reflected in divergent political attitudes towards firearm violence prevention policies.

Despite the importance of some consumer brand preferences to political and social identity, we are not aware of any previous study that has ascertained the brand preferences of gun owners, let alone tested whether there is a connection between gun owners’ brand preferences and their attitudes towards firearm violence prevention policies. The objective of our investigation was, therefore, to survey current gun owners and determine the relationship between their choices in the specific brands of guns they own and their support of firearm violence prevention policies. To the best of our knowledge, this is the first published, peer-reviewed study to ascertain the ownership of guns at the brand level.

We are aware of only one previous study that attempted to distinguish the political opinions of gun owners based on characteristics of their gun ownership. A recent poll found that those who own more than one gun are more likely to hold strong opinions about preserving gun rights than those who only own one gun (Zeballos-Roig & Hickey, 2019). However, to our knowledge, no studies have explored the relationship between the actual brands (e.g., Ruger, Glock, Smith & Wesson, etc.) or models (Px4 Storm SubCompact Type F, Buck Mark Standard Stainless URX, FN15 Tactical .300 BLK II, etc.) of guns a person owns and their gun-related policy opinions.

## Methods

### Sample and weighting

We conducted a national survey of 2086 gun owners using the Ipsos KnowledgePanel®, which is a 55,000 member adult internet panel whose members have agreed to take weekly internet-based surveys. Panel members are selected with known probability so that survey results can be weighted to properly reflect the U.S. population with a measurable level of accuracy, something that is not obtainable from non-probability panels. The panel is recruited using a national Address Based Sampling (ABS) methodology via the Delivery Sequence File of the U.S. Postal Service.

For our custom survey, a random sample of 3698 panelists who had reported owning a gun when they were recruited to the panel were invited by email to participate. Of these, 2321 (62.8%) responded by clicking on the link to go the screener page, and 2086 of those screened (89.9%) completed the survey. Therefore, the American Association for Public Opinion Research response rate 1 (American Association for Public Opinion Research (AAPOR), 2016) was 56.5%. The screener page checked to make sure that the respondent was still a personal gun owner at the time of the survey. Ipsos calculated survey weights that accounted for the probability of panelist selection and for survey non-response. We used these weights in an attempt to make the results representative of all U.S. adult gun owners in 2019. We verified the representativeness of the sample by comparing its demographic characteristics with those of gun owners in the 2018 General Social Survey (NORC at the University of Chicago, 2018), which is generally considered the gold standard for national gun ownership data (Siegel & Boine, 2020). The study was deemed exempt from human subjects review by the Boston University Medical Center Institutional Review Board because the investigators did not collect any personally identifiable data.

### Survey

The survey first ascertained the number of guns that respondents owned in each of four categories: pistols, revolvers, rifles, and shotguns. Within each relevant category, survey participants were then asked to identify each of the brands within that category that they own (e.g., Ruger, Glock, Smith & Wesson, etc.). Within each identified brand, they were then asked to identify which specific models they owned (e.g., Ruger Precision Rifle, Glock G45P pistol, Smith & Wesson Model 610 10 mm revolver, etc.). For each weapon type and brand, participants had the option to choose “Other” if their brand or weapon type did not appear in the list. If so, they were asked to identify the brand and model as specifically as they could.

### Identification of the most common firearm brands

In order to ensure that we listed as many of the brands as possible that were most likely to be owned but not including so many options that the survey was unmanageable, we identified the top 20 firearm brands using firearm production data maintained by the Bureau of Alcohol, Tobacco, Firearms, & Explosives and published annually as its Annual Firearms Manufacturing and Export Report (AFMER) (ATF (Bureau of Alcohol, Tobacco, Firearms,, and Explosives), 2020). We used the 2017 report since it was the most recently published at the time we created the survey. The identified brands represented 85.3% of the overall market share of domestic firearm production in 2017 (Supplementary Table 1, Additional File 1). By weapon type, the market shares represented by the brands we included in the survey were 91.7% for pistols, 87.7% for revolvers, 82.0% for rifles, and 93.9% for shotguns. Together, these brands accounted for a total of 6.3 million of the 7.1 million guns manufactured in 2017, and their market share has remained relatively stable over the past decade (Smith et al., 2017).

We supplemented this list by conducting internet searches to identify popular brands that we may have missed as well as to identify brands of tactical weapons (e.g., military-style weapons) that may not have appeared among the top brands by production market share. Our final list consisted of 25 brands of pistols, seven brands of revolvers, 35 brands of rifles, and 10 brands of shotguns (Supplementary Table 2, Additional File 2).

### Identification and categorization of firearm models

For each brand, we examined online catalogs for 2018 and 2019 to compile a list of all gun models currently being produced. We created a database containing each model and recording the following characteristics: type of weapon, brand, model, model number, ammunition used (pistols, revolvers, rifles) or chamber size and gauge (shotguns), length, and magazine capacity (Supplementary Appendix, Additional File 3).

Each firearm model was then categorized into one of three groups based on the primary purpose or use of the gun: recreation, self-defense, and tactical. This classification was based primarily on the way in which the model was displayed and marketed in the gun catalog. We also considered the model name, type of ammunition, length, and magazine capacity. For instance, guns classified as self-defense generally emphasized concealability and personal safety. Those classified as tactical were typically portrayed as military-style weapons. Firearms classified in the recreation category typically emphasized their use for hunting, target shooting, or competitive shooting. A detailed classification definition is shown in Table 1.

**Table 1:** Categorical definitions for types of guns by predominant marketing use

Category	Definition
Recreational	The primary intended use of the firearm appears to be for hunting, recreational shooting (e.g., target shooting, shooting competitions), or collecting. Imagery may include pictures of outdoor hunting-related scenes, animals, or targets or indoor shooting ranges. The name of the gun may include hunting themes or intended targets, such as “varmint.”
Self-Defense	The primary intended use of the firearm appears to be for self-defense, including concealed carry. The name of the gun may include references to self-defense or concealed carry. Features of the gun, such as a short barrel and easy concealability may help to indicate an intended self-defense or concealed carry use.
Tactical	The primary marketing or portrayal of the firearm is related to simulating military weapons. Imagery may portray military-style outerwear, camouflage, other military themes, or tactical gear. The firearm typically has additional features, including a telescoping stock, optics, a pistol grip, or high capacity magazines. The firearm is typically a version of a military weapon.

In categorizing models in the self-defense category, we did not distinguish between self-defense in the home and concealed carry. In differentiating between the recreation and tactical categories, we did consider the imagery used in the catalogs. For example, a rifle marketed with intense military images would be classified as tactical, while a similar rifle with images of shooting competitions would be classified as recreational.

### Grouping of gun owners based on brands/models of guns owned

Using this classification of gun models into the categories of recreational, defense, and tactical, we identified—for each gun owner—the number of guns in each of these three categories. Respondents were then divided into three groups based on the classification of their gun models: (1) Tactical—ownership of any tactical firearm; (2) Defense—ownership of any defense firearm (absent ownership of a tactical firearm); and (3) Recreation—ownership of only recreational firearms.

### Policy attitudes

Support for seven specific firearm laws was assessed using a five-point Likert scale with responses of strongly agree, agree, neither agree nor disagree, disagree, and strongly disagree. We classified respondents who replied “strongly agree” or “agree” as being in support of the policy. The seven policies assessed were: (1) bans on high capacity ammunition magazines; (2) bans on “assault weapons”; (3) banning concealed carry on college campuses; (4) increase the legal age of gun purchase to 21; (5) universal background checks; (6) permit requirements for gun purchase and (7) red flag laws (also called extreme risk protection orders) that allow confiscation of guns from people who are deemed by a judge to be a danger to themselves or others. These seven policies were chosen because they are the most common ones currently being considered by state legislatures.

### Data analysis

The significance of differences between groups in the proportion of respondents supporting a particular policy was assessed using a Z-test for the difference in proportions. Analyses were conducted in Stata version 15 (College Station, TX: StataCorp).

## Results

### Sample representativeness

The demographics of the sample were comparable to those of gun owners in the nationally representative 2018 General Social Survey, conducted by NORC at the University of Chicago (2018) (Siegel & Boine, 2020). It should be noted, however, that the generalizability of any survey study of gun owners may be limited because a certain segment of gun owners may be less likely to take part in surveys (Smith et al., 2015).

### Descriptive results

A total of 1477 of the 2086 survey respondents (70.8%) reported owning one or more pistols. By far, the most common brands were Smith & Wesson (owned by 30.7% of pistol owners), Ruger (25.5%), and Glock (23.2%) (Table 2). A total of 816 respondents (39.1%) reported owning a revolver. The most common brands were Smith & Wesson (owned by 46.8% of revolver owners) and Ruger (26.8%).

**Table 2:** Firearm brands reported by survey respondents, 2019 National Lawful Use of Guns Survey

PISTOLS				REVOLVERS			
Brand	Number who own brand	Percentage of all survey respondents who own brand	Percentage of pistol owners who own brand	Brand	Number who own brand	Percentage of all survey respondents who own brand	Percentage of revolver owners who own brand
Smith & Wesson	453	21.7	30.7	Smith & Wesson	382	18.3	46.8
Ruger	377	18.1	25.5	Ruger	219	10.5	26.8
Glock	343	16.4	23.2	Other	165	7.9	20.2
Other	316	15.1	21.4	Colt	138	6.6	16.9
Colt	183	8.8	12.4	Taurus	94	4.5	11.5
Beretta	163	7.8	11.0	Heritage	24	1.2	2.9
Taurus	149	7.1	10.1	North American	20	1.0	2.5
Remington	146	7.0	9.9	Kimber	11	0.5	1.3
Sig Sauer	132	6.3	8.9	**TOTAL**	**816**	**39.1**	**100**
Springfield	121	5.8	8.2				
Browning	100	4.8	6.8				
Kimber	53	2.5	3.6				
Hi-Point	40	1.9	2.7				
Heckler & Koch	38	1.8	2.6				
Mossberg	37	1.8	2.5				
Kel-Tec	28	1.3	1.9				
SCCY	18	0.9	1.2				
Century Arms	14	0.7	0.9				
North American	11	0.5	0.7				
FN America	11	0.5	0.7				
Bushmaster	9	0.4	0.6				
Diamondback	8	0.4	0.5				
Palmetto	6	0.3	0.4				
Steyr	5	0.2	0.3				
Alexander	2	0.1	0.1				
Christensen	1	0.0	0.1				
Radical	0	0.0	0.0				
**TOTAL**	**1477**	**70.8**	**100**				
RIFLES				SHOTGUNS			
Brand	Number who own brand	Percentage of all survey respondents who own brand	Percentage of rifle owners who own brand	Brand	Number who own brand	Percentage of all survey respondents who own brand	Percentage of shotgun owners who own brand
Remington	449	21.5	34.3	Remington	468	22.4	39.9
Other	313	15.0	23.9	Other	306	14.7	26.1
Winchester	304	14.6	23.2	Mossberg	270	12.9	23.0
Ruger	212	10.2	16.2	Winchester	221	10.6	18.8
Marlin	179	8.6	13.7	Browning	156	7.5	13.3
Savage	131	6.3	10.0	Savage	67	3.2	5.7
Browning	128	6.1	9.8	Beretta	34	1.6	2.9
Smith & Wesson	90	4.3	6.9	Century Arms	7	0.3	0.6
Mossberg	78	3.7	6.0	Kel-Tec	7	0.3	0.6
Springfield	69	3.3	5.3	Henry USA	5	0.2	0.4
Henry USA	58	2.8	4.4	FN America	1	0.0	0.1
Colt	49	2.3	3.7	**TOTAL**	**1174**	**56.3**	**100**
Bushmaster	41	2.0	3.1				
Unique ARs	25	1.2	1.9				
Beretta	17	0.8	1.3				
Palmetto	14	0.7	1.1				
Sig Sauer	14	0.7	1.1				
Hi-Point	12	0.6	0.9				
Armalite	11	0.5	0.8				
Century Arms	11	0.5	0.8				
Heckler & Koch	8	0.4	0.6				
Anderson	7	0.3	0.5				
Daniel Defense	7	0.3	0.5				
Kel-Tec	6	0.3	0.5				
Barrett	5	0.2	0.4				
Diamondback	5	0.2	0.4				
Kimber	5	0.2	0.4				
Alexander	4	0.2	0.3				
Eagle Arms	2	0.1	0.2				
FN America	2	0.1	0.2				
JP Enterprises	2	0.1	0.2				
Steyr	2	0.1	0.2				
Desert Tech	1	0.0	0.1				
Radical	1	0.0	0.1				
Christensen	0	0.0	0.0				
DRD Tactical	0	0.0	0.0				
F&D Defense	0	0.0	0.0				
Noreen Firearms	0	0.0	0.0				
**TOTAL**	**1310**	**62.8**	**100**				

A total of 1310 respondents (62.8%) reported owning one or more rifles. By far, the most common brand was Remington (owned by 34.3% of rifle owners), followed by Winchester (23.2%) and Ruger (16.2%) (Table 2). A total of 1174 respondents (56.3%) reported owning a shotgun. The most common brands were Remington (owned by 39.9% of shotgun owners), Mossberg (23.0%), and Winchester (18.8%).

We were able to identify the specific models for a total of 4337 guns among 1218 respondents (Table 3). Of these guns, 2362 (54.5%) were classified as Recreational, 1497 (34.5%) as Defense, and 478 (11.0%) as Tactical. Of the 1218 gun owners for whom we were able to identify one or more gun models, 880 (72.2%) owned a Recreational gun, 730 (59.9%) owned a Defense gun, and 257 (21.1%) owned a Tactical gun. Based on their ownership of these three types of guns, the categorization of these 1218 gun owners was 407 (33.4%) Recreational gun model ownership, 554 (45.5%) Defense gun model ownership, and 257 (21.1%) Tactical gun model ownership. For the other 868 gun owners, we were unable to identify any specific gun models because either they failed to check any of the models listed in the survey or because they checked “other” but failed to provide enough information for us to identify the model.

**Table 3:** Number of classified gun models by category: Recreational, defense, and tactical—National Lawful Use of Guns Survey, 2019

Type	Number who own	Percent who own	Total number of guns owned	Percent of all classified guns
Recreation	880	72.2	2362	54.5
Defense	730	59.9	1497	34.5
Tactical	257	21.1	478	11.0
**Total**	**1218**		**4337**	**100**
Combination of gun types possessed by a gun owner		Number of gun owners in category		Percent of gun owners in category
Recreational only		407		33.4
Defense only		262		21.5
Tactical only		33		2.7
Recreational and defense		292		24.0
Recreational and tactical		48		3.9
Defense and tactical		43		3.5
All three types		133		10.9
**Total**		**1218**		**100**
Final categorization of gun owners with identified models		Number in category		Percent in category
Recreational (owns recreational guns only)		407		33.4
Defense (owns a defense gun, but no tactical)		554		45.5
Tactical (owns a tactical gun)		257		21.1
**Total**		**1218**		**100.0**
Final categorization of all gun owners		Number in category		Percent in category
Recreational (owns recreational guns only)		407		19.5
Defense (owns a defense gun, but no tactical)		554		26.6
Tactical (owns a tactical gun)		257		12.3
No identified gun model		868		41.6
**Total**		**2086**		**100.0**

### Validity of gun model ownership categories

To test the validity of our gun model ownership categories, we compared a variety of gun-related practices and attitudes between the three ownership groups (recreational, defense, and tactical) (Table 4). For each practice and attitude, we found a monotonic increase in the proportion of gun owners moving from the recreational to the defense to the tactical gun owners, and a chi-square test for trend was statistically significant in each case. For example, the percentage of NRA members increased steadily from 10.6% in the recreational gun model group to 16.1% in the self-defense gun model group to 26.1% in the tactical gun model group.

**Table 4:** Gun-related attitudes and practices by gun model category (percentages and 95% confidence intervals)

Attitude or Practice	Gun ownership category		
	Recreation	Defense	Tactical
**Gun-related practices**			
Shot favorite gun in past month^***^	14.1%^*^ (10.4, 18.7)	19.4%^†^ (15.9, 23.4)	39.0%*^†^ (32.2, 46.2)
Carried favorite gun in past month^***^	26.5%^*^ (21.8, 31.7)	44.3%^*^ (39.6, 49.1)	55.6%^*^ (48.6, 62.4)
Carry concealed gun monthly or more^***^	15.3%^*^ (11.5, 20.1)	40.0%^*^ (35.4, 44.7)	53.2%^*^ (46.3, 60.1)
NRA member^***^	10.6%^*^ (7.7, 14.5)	16.1%^*^ (12.9, 19.9)	26.1%^*^ (20.4, 32.8)
Own any detachable magazine^***^	51.3%^*^ (45.8, 56.8)	70.9%^*^ (66.5, 75.0)	87.6%^*^ (82.3, 91.5)
Subscribe to any gun-related magazine^***^	15.3%^*^ (11.4, 20.1)	20.1%^†^ (16.5, 24.2)	33.7%^*†^ (27.3, 40.8)
Participate in any monthly gun-related activity^***^	19.8%^*^ (15.4, 25.0)	27.8%^*^ (23.8, 32.2)	50.5%^*^ (43.5, 57.4)
**Gun-related attitudes**			
Strongly agrees with any gun identity statement^***^	12.7%^*†^ (9.6, 16.6)	18.9%^*^ (15.3, 23.0)	24.7%^†^ (19.3, 31.1)
Agrees: I act like gun owners to a great extent^***^	10.1%^*†^ (7.2, 14.1)	15.9%^*^ (12.6, 19.9)	20.7%^†^ (15.8, 26.8)
Agrees: Gun owners’ successes are my successes^***^	15.6%^*^ (11.9, 20.2)	22.0%^*^ (18.3, 26.2)	29.6%^*^ (23.7, 36.2)
Agrees: Guns are important to my identity^***^	9.8%^*^ (6.8, 13.9)	10.9%^†^ (8.1, 14.5)	18.6%^*†^ (13.8, 24.7)
Agrees: Owning a gun is essential to my sense of freedom^***^	59.9%^*†^ (54.4, 65.1)	69.3%^*^ (64.8, 73.4)	75.7%^†^ (69.1, 81.2)
Agrees: Guns make me feel responsible^***^	44.2%^*†^ (38.9, 49.7)	58.3%^*^ (53.5, 62.8)	62.3%^†^ (55.4, 68.7)

^*^Values are significantly different at p < 0.05

^†^Values are significantly different at p < 0.05

^***^Chi-square test for trend is significant at *p* < 0.05

### Findings by gun type

There were only minor differences between owners of different types of guns (i.e., pistols, revolvers, rifles, and shotguns) in their attitudes toward high capacity ammunition magazine bans, with support ranging from 32.1% among pistol owners to 34.3% among shotgun owners (Table 5). In fact, there were only minor differences in attitudes towards almost all of the seven firearm violence prevention policy measures, with support differing by no more than five percentage points for any of these laws except for age restrictions on gun purchase.

**Table 5:** Attitudes toward firearm violence prevention policies by gun brand and type

Brand	% (95% confidence interval)						
	Supports ban on high capacity ammunition magazines	Supports ban on assault weapons	Supports ban on concealed gun carrying at colleges	Supports ban on gun purchase by anyone less than 21	Supports universal background checks	Supports permit requirement to purchase a firearm	Supports red flag laws (extreme risk protection order laws)
Kimber pistol (*N* = 53)	12.9% (5.1, 28.9)	13.2% (5.3, 29.1)	11.1% (4.0, 27.5)	23.6% (12.3, 40.5)	55.9% (40.6, 70.0)	33.4% (20.0, 50.2)	54.4% (38.5, 69.4)
Springfield pistol (*N* = 121)	14.5% (8.7, 23.1)	16.0% (9.6, 25.7)	11.7% (7.2, 18.5)	27.7% (19.7, 37.5)	69.1% (58.8, 77.8)	39.2% (29.8, 49.4)	72.1% (61.3, 80.8)
Sig Sauer pistol (*N* = 132)	15.0% (9.7, 22.4)	21.1% (14.8, 29.2)	21.7% (15.0, 30.3)	32.6% (24.3, 42.3)	70.1% (59.8, 78.7)	43.1% (33.8, 52.9)	78.0% (67.8, 85.6)
Henry USA rifle (*N* = 58)	17.0% (8.6, 31.0)	19.9% (10.6, 34.1)	23.6% (12.4, 40.2)	20.8% (10.6, 36.9)	62.3% (47.4, 75.2)	45.2% (30.8, 60.5)	79.9% (66.6, 88.8)
Ruger rifle (*N* = 212)	22.3% (16.5, 29.4)	23.4% (17.6, 30.6)	23.8% (18.1, 30.5)	26.4% (19.9, 34.3)	64.4% (56.9, 71.2)	39.4% (32.0, 47.2)	71.2% (63.9, 77.5)
Savage rifle (*N* = 131)	23.0% (16.2, 31.6)	24.6% (17.6, 33.4)	25.9% (18.6, 34.8)	20.0% (13.3, 29.0)	61.1% (51.5, 69.8)	31.7% (23.3, 41.3)	72.0% (62.4, 80.0)
Beretta pistol (*N* = 163)	23.7% (16.9, 32.3)	35.1% (27.2, 43.9)	28.4% (21.3, 36.6)	40.2% (32.0, 49.0)	65.3% (56.6, 73.0)	51.0% (42.3, 59.6)	78.9% (70.3, 85.6)
Taurus pistol (*N* = 149)	24.1% (17.0, 33.0)	24.0% (16.7, 33.3)	23.9% (16.8, 33.0)	38.4% (29.6, 48.1)	69.0% (59.6, 77.0)	49.4% (39.8, 59.0)	77.6% (68.4, 84.8)
Mossberg shotgun (*N* = 270)	24.1% (19.1, 30.0)	24.0% (18.7, 30.2)	20.3% (15.8, 25.6)	33.9% (27.5, 41.0)	67.0% (60.3, 73.0)	43.7% (37.0, 50.6)	79.1% (73.1, 84.1)
Springfield rifle (*N* = 69)	24.3% (13.5, 39.8)	22.9% (13.4, 36.3)	28.3% (17.0, 43.1)	21.5% (11.9, 35.8)	57.9% (44.1, 70.6)	39.2% (26.8, 53.1)	77.7% (64.0, 87.2)
Glock pistol (*N* = 343)	24.4% (19.7, 30.0)	28.5% (23.4, 34.3)	27.9% (22.8, 33.5)	39.2% (33.4, 45.3)	72.2% (66.5, 77.3)	49.7% (43.6, 55.7)	79.8% (74.5, 84.3)
Ruger revolver (*N* = 219)	26.8% (20.5, 34.1)	29.4% (22.9, 36.7)	25.1% (19.1, 32.2)	25.4% (19.4, 32.4)	65.7% (58.1, 72.6)	40.4% (33.2, 48.1)	78.4% (70.9, 84.4)
Marlin rifle (*N* = 179)	27.4% (21.0, 34.9)	29.6% (22.9, 37.3)	32.3% (25.1, 40.6)	18.4% (13.2, 25.2)	66.6% (58.7, 73.6)	38.7% (31.1, 46.9)	81.0% (73.7, 86.6)
Smith and Wesson pistol (*N* = 453)	27.8% (23.5, 32.6)	32.7% (28.0, 37.8)	28.8% (24.3, 33.8)	35.0% (30.1, 40.2)	70.0% (64.9, 74.6)	46.9% (41.6, 52.2)	78.1% (73.3, 82.3)
Browning pistol (*N* = 100)	29.3% (20.2, 40.5)	33.4% (24.3, 43.9)	31.8% (22.4, 42.8)	35.9% (26.3, 46.9)	58.1% (47.1, 68.4)	42.6% (32.4, 53.4)	78.3% (67.8, 86.1)
Ruger pistol (*N* = 377)	30.2% (25.2, 35.7)	32.5% (27.5, 38.1)	27.6% (22.9, 32.8)	34.1% (28.8, 39.9)	72.0% (66.8, 76.7)	42.9% (37.4, 48.7)	81.1% (76.3, 85.2)
Browning rifle (*N* = 128)	30.2% (22.1, 39.7)	32.3% (23.9, 42.0)	31.0% (22.4, 41.2)	24.6% (17.2, 33.8)	62.8% (52.5, 72.1)	48.5% (38.6, 58.6)	75.5% (65.0, 83.7)
Colt pistol (*N* = 183)	30.9% (24.1, 38.8)	33.1% (26.1, 41.0)	32.5% (25.4, 40.5)	36.6% (29.2, 44.8)	70.0% (62.2, 76.7)	49.0% (41.0, 57.0)	82.1% (75.1, 87.5)
Remington shotgun (*N* = 468)	31.6% (27.1, 36.4)	33.9% (29.2, 39.0)	34.6% (29.9, 39.6)	29.8% (25.1, 34.9)	70.1% (65.3, 74.6)	47.2% (42.1, 52.4)	79.7% (75.1, 83.7
*Any pistol (N = 1477)*	*32.1% (29.5, 34.8)*	*36.4% (33.7, 39.2)*	*31.8% (29.1, 34.5)*	*40.0% (37.1, 42.9)*	*74.3% (71.7, 76.8)*	*50.8% (47.9, 53.7)*	*80.5% (78.0, 82.7)*
Smith and Wesson rifle (*N* = 90)	32.8% (22.6, 44.9)	32.6% (22., 44.5)	27.0% (17.5, 39.2)	37.4% (26.6, 49.5)	75.6% (64.8, 83.9)	51.8% (39.9, 63.5)	83.8% (73.1, 90.8)
*Any rifle (N = 1310)*	*32.9% (30.1, 35.8)*	*33.5% (30.7, 36.4)*	*32.1% (29.3, 35.0)*	*31.1% (28.3, 34.1)*	*71.1% (68.3, 73.8)*	*46.1% (43.1, 49.2)*	*79.3% (76.6, 81.7)*
Browning shotgun (*N* = 156)	32.9% (25.4, 41.4)	35.7% (28.0, 44.2)	31.7% (24.3, 40.3)	23.8% (17.0, 32.3)	62.7% (53.9, 70.8)	46.0% (37.5, 54.7)	83.4% (75.9, 88.9)
Taurus revolver (*N* = 94)	34.2% (24.0, 46.1)	38.3% (27.5, 50.5)	23.9% (15.5, 34.9)	34.1% (24.3, 45.5)	66.0% (54.6, 75.9)	51.7% (40.1, 63.0)	74.2% (62.8, 83.0)
*Any revolver (N = 816)*	*34.1% (30.6, 37.8)*	*36.3% (32.7, 40.2)*	*31.7% (28.3, 35.4)*	*33.2% (29.6, 37.0)*	*69.1% (65.4, 72.6)*	*47.3% (43.4, 51.2)*	*78.7% (75.1, 81.9)*
*Any shotgun (N = 1174)*	*34.3% (31.3, 37.5)*	*35.1% (32.1, 38.3)*	*31.4% (28.5, 34.5)*	*31.4% (28.4, 34.7)*	*70.8% (67.8, 73.7)*	*47.9% (44.6, 51.2)*	*80.1% (77.3, 82.6)*
Colt revolver (*N* = 138)	34.9% (26.5, 44.5)	36.7% (27.8, 46.5)	37.7% (28.7, 47.6)	28.7% (20.9, 38.0)	62.5% (52.3, 71.6)	45.3% (35.8, 55.2)	71.3% (60.6, 80.1)
Smith and Wesson revolver (*N* = 382)	36.1% (30.9, 41.6)	36.4% (31.2, 42.0)	31.7% (26.6, 37.2)	35.8% (30.6, 41.3)	68.7% (63.3, 73.7)	47.9% (42.2, 53.6)	77.1% (71.5, 81.8)
Savage shotgun (*N* = 67)	36.6% (24.9, 50.0)	26.9% (17.2, 39.4)	29.6% (19.3, 42.5)	12.8% (6.2, 24.6)	58.4% (44.8, 70.9)	41.1% (28.2, 55.3)	87.8% (76.8, 94.0)
Winchester rifle (*N* = 304)	36.8% (31.0, 42.9)	32.4% (26.9, 38.4)	33.4% (27.9, 39.4)	25.1% (19.8, 31.3)	66.6% (60.6, 72.2)	43.3% (37.1, 49.6)	82.6% (77.5, 86.7)
***Owns any of the 82 listed brands (N = 1883)***	***37.1% (34.7, 39.6)***	***40.3% (37.8, 42.8)***	***35.4% (33.0, 37.9)***	***38.5% (36.0, 41.1)***	***75.3% (73.1, 77.4)***	***52.9% (50.3, 55.5)***	***81.7% (79.6, 83.7)***
Remington rifle (*N* = 449)	37.3% (32.5, 42.4)	36.8% (32.0, 41.9)	31.9% (27.4, 36.9)	29.5% (24.8, 34.6)	71.9% (67.1, 76.3)	44.9% (39.8, 50.1)	81.1% (76.7, 84.8)
Mossberg rifle (*N* = 78)	37.3% (26.0, 50.1)	30.1% (19.9, 42.8)	27.9% (18.1, 40.3)	33.3% (22.5, 46.2)	72.4% (60.1, 82.1)	52.0% (39.4, 64.4)	83.1% (69.3, 91.5)
**Total sample (*N*** **= 2086)**	**37.8% (35.5, 40.1)**	**41.2% (38.9, 43.6)**	**35.6% (33.3, 37.9)**	**39.4% (37.0, 41.9)**	**75.0% (72.8, 77.1)**	**53.0% (50.6, 55.4)**	**81.4% (79.4, 83.3)**
Winchester shotgun (*N* = 221)	38.9% (31.7, 46.8)	39.0% (31.8, 46.7)	35.0% (27.7, 43.0)	24.1% (18.0, 31.4)	72.3% (65.0, 78.5)	45.2% (37.6, 53.0)	84.7% (77.8, 89.8)
Remington pistol (*N* = 146)	39.5% (31.0, 48.8)	42.2% (33.4, 51.5)	36.3% (28.2, 45.2)	42.7% (33.9, 52.1)	77.9% (69.7, 84.3)	56.3% (47.1, 65.0)	87.1% (80.1, 91.9)
***Does not own any of the listed brands (N = 203)***	***44.1% (36.5, 52.0)***	***49.8% (41.9, 57.7)***	***37.1% (29.9, 44.9)***	***47.6% (39.8, 55.6)***	***72.2% (64.1, 79.1)***	***53.9% (45.9, 61.7)***	***78.6% (70.8, 84.8)***

*Note:* Only brands used by at least 50 gun owners are shown in table

### Findings by gun brand

In contrast to the findings by gun type, there were large differences between owners of different gun brands in their attitudes towards high capacity magazine bans, with support ranging from a low of 12.9% among owners of Kimber pistols to a high of 39.5% among those who owned Remington pistols (Table 5). There was also a substantial difference in support for magazine bans based on whether or not a gun owner reported owning one of the 82 brands listed in the survey, with respondents who did not own any of the listed brands demonstrating the highest level of support for these policies.

The brand-specific differences in support for high capacity ammunition magazine bans were mirrored by similar differences in support for bans on “assault weapons” and bans on concealed carry on college campuses (Table 5). For example, owners of Kimber pistols showed the lowest level of support for all three of these policies (between 11 and 13%) and owners of Remington pistols showing the highest level of support for all three of these policies (between 36 and 42%). This pattern was also evident in attitudes towards a permit requirement for firearm purchase, with Kimber pistol owners showing the second lowest level of support (33.4%) and Remington pistol owners showing the highest level of support (56.3%). These patterns were less clear for universal background checks and red flag laws, both of which had high levels of support across the board. Nevertheless, there were still substantial differences in support for these policies among gun owners. For example, only 55.9% of Kimber pistol owners supported universal background checks compared to 77.9% of Remington pistol owners. Similarly, only 54.4% of Kimber pistol owners supported red flag laws compared to 87.1% of Remington pistol owners.

### Findings by gun model ownership

Support for “assault weapon” bans was significantly higher among the Recreation group compared to the Defense group and among the Defense group compared to the Tactical group (Table 6). Support for banning concealed carry on college campuses was significantly higher among the Recreation group compared to the Defense and Tactical groups. For universal background checks, gun permit requirements, and red flag laws, support was significantly lower among the Tactical group than the Recreation and Defense gun model group. However, there were no significant differences between these three groups in support for age restrictions for gun purchase. Gun owners for whom we could not identify any gun models expressed the highest level of support for all but one of the gun policies. The only significant difference in party affiliation between groups was an increased proportion of Democrats in the no model group compared to the Tactical group.

**Table 6:** Attitudes towards firearm violence prevention policies by gun model category (percentages and 95% confidence intervals)

Policy	Gun ownership category			No identified gun model
	Recreation	Defense	Tactical	
Supports large capacity magazine ban	47.0%^*^ (41.6, 52.5)	28.9%^*†^ (24.9, 33.4)	14.6%^*†^ (10.2, 20.3)	47.3%^†^ (43.5, 51.0)
Supports assault weapon ban	46.5%^*^ (41.1, 52.0)	35.2%^*†^ (30.8, 39.9)	18.0%^*†^ (13.2, 23.9)	50.5%^†^ (46.7, 54.3)
Supports banning concealed carry on college campuses	42.9%^*†^ (37.6, 48.4)	29.7%^*‡^ (25.5, 34.2)	22.4%^†§^ (17.2, 28.6)	40.7%^‡§^ (37.1, 44.4)
Supports ban on gun purchase by anyone less than 21	33.6%^*^ (28.5, 39.2)	38.2%^†^ (33.6, 42.9)	32.5%^‡^ (26.2, 39.4)	45.0%^*†‡^ (41.2, 48.8)
Supports universal background checks	77.4%^*^ (72.7, 81.5)	75.8%^†^ (71.6, 79.6)	61.0%^*†‡^ (54.0, 67.5)	78.0%^‡^ (74.6, 81.0)
Supports permit requirement to purchase a firearm	52.3%^*^ (46.8, 57.7)	52.5%^†^ (47.7, 57.2)	41.7%^*†‡^ (34.9, 48.7)	57.3%^‡^ (53.5, 61.0)
Supports red flag laws (extreme risk protection order laws)	81.5%^*^ (76.8, 85.4)	83.3%^†^ (79.5, 86.6)	72.6%^*†‡^ (65.7, 78.6)	82.9%^‡^ (79.7, 85.7)
**Political party**				
Republican	52.1% (46.6, 57.5)	54.2% (49.4, 58.9)	55.9% (48.9, 62.7)	47.7% (44.0, 51.5)
Independent	23.0% (18.6, 28.0)	20.4% (16.7, 24.6)	25.1% (19.6, 31.5)	23.7% (20.5, 27.3)
Democrat	25.0% (20.6, 29.9)	25.5% (21.5, 30.0)	19.0%^*^ (14.2, 24.9)	28.6%^*^ (25.3, 32.1)

^*^Values are significantly different at p < 0.05

^†^Values are significantly different at *p* < 0.05

^‡^Values are significantly different at *p* < 0.05

^§^Values are significantly different at p < 0.05

### Relationship between brand and policy support after controlling for sociodemographic factors

We conducted a final analysis to determine whether the relationship between brand and policy support was attributable to differences in sociodemographic factors and political affiliation or ideology differences between owners of various gun brands. After controlling for age, gender, race/ethnicity, income level, political party, and political ideology, the relationship between ownership of gun brands and support for large capacity ammunition magazine bans was essentially unchanged (Table 7). Ownership of Kimber, Springfield, Sig Sauer, Glock, Mossberg, Savage, Ruger, Browning, Colt, and Smith & Wesson guns was significantly associated with lower support for this policy. Remington and Winchester gun owners were not significantly more likely to oppose magazine bans.

**Table 7:** Likelihood of supporting a ban on high capacity ammunition magazines by brand (types combined) of gun owned, controlling for age group, gender, race/ethnicity, income level, political party, and political ideology

	Odds Ratio
**Brand**	**(95% confidence interval)**
Henry USA (*N* = 62)	0.25^*^ (0.12–0.55)
Sig Sauer (*N* = 140)	0.35^*^ (0.21–0.59)
Springfield (*N* = 175)	0.36^*^ (0.22–0.59)
Kimber (*N* = 65)	0.37^*^ (0.16–0.83)
Glock (N = 343)	0.57^*^ (0.41–0.79)
Ruger (*N* = 598)	0.61^*^ (0.47–0.77)
Savage (*N* = 178)	0.62^*^ (0.43–0.90)
Beretta (*N* = 192)	0.66 (0.41–1.05)
Colt (*N* = 296)	0.66^*^ (0.49–0.90)
Marlin (*N* = 179)	0.68 (0.45–1.03)
Smith and Wesson (*N* = 702)	0.68^*^ (0.53–0.86)
Mossberg (*N* = 317)	0.69^*^ (0.51–0.95)
Taurus (*N* = 198)	0.70 (0.48–1.04)
Browning (*N* = 305)	0.70^*^ (0.51–0.95)
***Owns any of the 82 listed brands (N = 1883)***	*0.81 (0.56–1.17)*
Remington (*N* = 785)	0.99 (0.78–1.24)
Winchester (*N* = 449)	1.18 (0.89–1.56)
***Does not own any of the listed brands (N = 203)***	*1.23 (0.81–1.79)*

^*^Statistically significant at *p* < .05, compared to all other gun owners (who do not own that brand)

*Note:* Only brands used by at least 50 gun owners are shown in table. Logistic regression analysis controls for gender, age group, race/ethnicity, income level, political party, and political ideology

## Discussion

To our knowledge, this is the first paper to ascertain the brands and models of guns owned by U.S. gun owners and to relate them to support for firearm violence prevention policies. Several important findings emerged. First, gun owners are extremely brand conscious and gun ownership is highly concentrated among a small number of brands. Second, the gun brands owned by an individual, but not the type of gun, are a strong predictor of that person’s attitudes regarding firearm violence prevention policies. Third, the specific models of guns owned strongly predicts a gun owner’s policy opinions.

The magnitude of brand-specific differences in attitudes toward firearm violence prevention policies was substantial. There was a three-fold difference between brands in the highest and lowest proportions of gun owners who support high capacity ammunition magazine bans, “assault weapon” bans, and bans on the concealed carrying of guns on college campuses. Similarly striking was the finding of a relationship between the gun model ownership group (Recreational, Defense, or Tactical) and the policy opinions of gun owners. Moving from the Recreational to the Defense to the Tactical groups, there was a monotonic decrease in support for three firearm violence prevention policies with a two- to three-fold difference in support between the Recreational and Tactical groups. Gun owners for whom we could not identify a gun model generally had the highest level of support for firearm violence prevention policies.

Importantly, we did not find any relationship between the gun models owned and a gun owner’s political party affiliation, except for significantly more Democrats in the no model group compared to the Tactical group. However, this difference is not enough to explain the policy differences between these groups. For example, support for high capacity magazine bans among the group with no identified gun models was 38.6% even after excluding Democrats, compared to just 14.6% for the Tactical group, even with Democrats included. In addition, the relationship between gun brand identity and public opinion held even after controlling for age, sex, race, socioeconomic status, and political affiliation and ideology.

Consistent with our initial premise, it appears that differences in political opinions among gun owners are associated with the brands and models that they choose. The use of specific brands and models appears to be an important part of gun owner identity and as such, they are tied to the owner’s political identity as it pertains to firearm violence prevention policy, extending beyond formal political party affiliation and political ideology. These results suggest that specific brands, more than just types of guns, are used to signal political opinions and that these opinions flow from different symbolic consumer behavior associated with different gun brands. It is also possible that purchase of a brand influences attitudes. Because this is a cross-sectional study, it is not possible to determine the direction of this relationship.

Our findings are also important because they may be the first demonstration that there is heterogeneity in the policy opinions *among* gun owners. In fact, the differences we observed between owners of various brands often exceeded differences in opinion regarding firearm violence prevention policies between gun owners and non-gun owners. For example, in 2017, there was less than a five percentage point difference in support for universal background checks between gun owners and non-gun owners nationally (Barry et al., 2018). In our survey, the difference in support for universal background checks between Kimber pistol owners (55.9%) and Remington pistol owners (77.9%) was 22 percentage points.

These findings have implications for public health practice. They suggest that public health practitioners trying to engage gun owners in firearm violence prevention may need to segment the audience. Messages that appeal to each segment are likely to be different. Separate communication strategies may be needed, for example, to effectively reach recreational, defense, and tactical gun ownership groups. The development, evaluation, and testing of potential segment-specific messages is necessary and will require further study.

There are several study limitations. First, there is a possibility of selection bias. The survey completion rate of 56%, while excellent for an internet-based panel study, still leaves the door open for non-response bias and weighting may not completely correct it. Second, it is possible that a certain segment of gun owners is reluctant to join survey response panels; this could create a bias towards finding greater support for gun policies. This would not diminish, however, from the differences seen between those gun owners who responded to the survey. Third, the findings of this paper apply only to the United States. Both consumer ownership of firearms and gun marketing are much less prevalent in most other nations. Fourth, we did not ascertain when guns were obtained, so it is possible that the intended use or marketing depiction of a gun at the time of acquisition was different from its use as depicted in 2018 and 2019 catalogs. Fifth, we tested only seven gun policies and cannot infer that the observed patterns would hold for all policies. Finally, the term “assault weapon” is not clearly defined in our survey and gun owners may have differing interpretations of the term’s meaning. Therefore, caution must be exercised in drawing conclusions about the reported attitudes of gun owners towards this particular policy. The same is true for the interpretation of “high capacity magazines.”

## Conclusion

Despite these limitations, this study provides new evidence that understanding the brand-and model-specific nature of gun ownership may help to distinguish subgroups of gun owners that systematically differ in their gun-related policy attitudes. This information could help public health practitioners develop segment-specific communications that will appeal to each group in order to more effectively engage gun owners in firearm violence prevention. In addition, our results suggest that further study of gun marketing practices may be useful in better understanding and perhaps influencing gun owners’ attitudes toward firearm violence prevention policies.

## Supplementary Information

**Additional File 1 Supplementary Table 1** Top 20 firearm manufacturing companies by domestic gun production and production market shares, 2017.

**Additional File 2 Supplementary Table 2** List of brands assessed in National Lawful Use of Guns Survey, 2019.

**Additional File 3 Supplementary Appendix** Gun types, brands, and models by predominant marketing use classification.

## Abbreviations

CDC: Centers for Disease Control and Prevention
ATF: Bureau of Alcohol, Tobacco, and Firearms
ABS: Address-based sampling
AAPOR: American Association for Public Opinion Research
AFMER: Annual Firearms Manufacturing and Export Report
