# Supplementary material for: The descriptive epidemiology of brand-specific gun ownership in the US: results from the 2019 National Lawful Use of Guns Survey
Source: Inj Epidemiol. 2021 Mar 22;8:12. doi: 10.1186/s40621-021-00305-1 (PMC7983377; doi:10.1186/s40621-021-00305-1)
Supplement: Supplementary file 1 — Additional File 1 Supplementary Table 1 Top 20 firearm manufacturing companies by domestic gun production and production market shares, 2017. [file 40621_2021_305_MOESM1_ESM.docx]

**Supplementary Table 1** Top 20 firearm manufacturing companies by domestic gun production and production market shares, 2017

| Company | Pistols | Revolvers | Rifles | Shotguns | TOTAL | Market share | Cumulative market share |
| --- | --- | --- | --- | --- | --- | --- | --- |
| Ruger | 771,089 | 168,066 | 621,540 | 16 | 1,560,711 | 22.0 | 22.0 |
| Smith and Wesson | 1,010,010 | 191,154 | 253,521 | 21 | 1,454,706 | 20.5 | 42.6 |
| Remington | 56,559 | 0 | 386,168 | 251,153 | 693,880 | 9.8 | 52.4 |
| Sig Sauer | 359,360 | 0 | 34,166 | 0 | 393,526 | 5.6 | 57.9 |
| Maverick Arms | 0 | 0 | 69,095 | 292,271 | 361,366 | 5.1 | 63.0 |
| Heritage | 0 | 226,063 | 0 | 0 | 226,063 | 3.2 | 66.2 |
| Henry Rac | 0 | 0 | 224,504 | 0 | 224,504 | 3.2 | 69.4 |
| Kimber | 181,253 | 21,015 | 10,519 | 0 | 212,787 | 3.0 | 72.4 |
| Springfield | 81,225 | 0 | 69,352 | 0 | 150,577 | 2.1 | 74.5 |
| SCCY | 150,377 | 0 | 0 | 0 | 150,377 | 2.1 | 76.6 |
| Kel Tec | 58,631 | 0 | 66,235 | 24,899 | 149,765 | 2.1 | 78.8 |
| Glock | 127,835 | 0 | 0 | 0 | 127,835 | 1.8 | 80.6 |
| Radical | 2,775 | 0 | 88,430 | 0 | 91,205 | 1.3 | 81.8 |
| Strassells | 46,015 | 0 | 40,511 | 0 | 86,526 | 1.2 | 83.1 |
| FN America | 60,874 | 0 | 15,353 | 0 | 76,227 | 1.1 | 84.1 |
| Beretta | 52,661 | 0 | 2,754 | 16,358 | 71,773 | 1.0 | 85.2 |
| Taurus | 68,848 | 0 | 103 | 0 | 68,951 | 1.0 | 86.1 |
| Browning | 50,331 | 0 | 668 | 453 | 51,452 | 0.7 | 86.9 |
| Colt | 30,117 | 7,184 | 13,942 | 0 | 51,243 | 0.7 | 87.6 |
| Diamondback | 24,270 | 0 | 26,960 | 0 | 51,230 | 0.7 | 88.3 |
| *Total Top 20* | 3,132,230 | 613,482 | 1,923,821 | 585,171 | 6,254,704 | *88.3* | *88.3* |
| **Total Market** | **3,415,586** | **699,241** | **2,345,221** | **623,142** | **7,083,190** | **100** | **100** |
| ***Top 20 Market Share*** | ***91.7*** | ***87.7*** | ***82.0*** | ***93.9*** | ***88.3*** | ***88.3*** | ***88.3*** |
