# Supplementary material for: The descriptive epidemiology of brand-specific gun ownership in the US: results from the 2019 National Lawful Use of Guns Survey
Source: Inj Epidemiol. 2021 Mar 22;8:12. doi: 10.1186/s40621-021-00305-1 (PMC7983377; doi:10.1186/s40621-021-00305-1)
Supplement: Supplementary file 2 — Additional File 2 Supplementary Table 2 List of brands assessed in National Lawful Use of Guns Survey, 2019. [file 40621_2021_305_MOESM2_ESM.docx]

**Supplementary Table 2** List of brands assessed in National Lawful Use of Guns Survey, 2019

| **Pistols** | **Revolvers** | **Rifles** | **Shotguns** |
| --- | --- | --- | --- |
| Alexander | Colt | Alexander | Beretta |
| Anderson | Heritage | Anderson | Browning |
| Beretta | Kimber | Armalite | Century Arms |
| Browning | North American Arms | Barrett | FN America |
| Bushmaster | Ruger | Beretta | Henry USA |
| Century Arms | Smith & Wesson | Browning | Kel-Tec |
| Christensen Arms | Taurus | Bushmaster | Mossberg |
| Colt |  | Century Arms | Remington |
| FN America |  | Christensen Arms | Savage |
| Glock |  | Colt | Winchester |
| Heckler & Koch |  | Daniel Defense |  |
| Hi-Point Firearms |  | Desert Tech |  |
| Kel-Tec |  | DRD Tactical |  |
| Kimber |  | Eagle Arms |  |
| Mossberg |  | F&D Defense |  |
| North American Arms |  | FN America |  |
| Palmetto State Armory |  | Heckler & Koch |  |
| Remington |  | Henry USA |  |
| Ruger |  | Hi-Point Firearms |  |
| SCCY Firearms |  | JP Enterprises |  |
| Sig Sauer |  | Kel-Tec |  |
| Smith & Wesson |  | Kimber |  |
| Springfield |  | Marlin |  |
| Steyr |  | Mossberg |  |
| Taurus |  | Noreen Firearms |  |
|  |  | Palmetto State Armory |  |
|  |  | Remington |  |
|  |  | Ruger |  |
|  |  | Savage |  |
|  |  | Sig Sauer |  |
|  |  | Smith & Wesson |  |
|  |  | Springfield |  |
|  |  | Steyr |  |
|  |  | Unique Ars |  |
|  |  | Winchester |  |
