# Supplementary material for: The descriptive epidemiology of brand-specific gun ownership in the US: results from the 2019 National Lawful Use of Guns Survey
Source: Inj Epidemiol. 2021 Mar 22;8:12. doi: 10.1186/s40621-021-00305-1 (PMC7983377; doi:10.1186/s40621-021-00305-1)
Supplement: Supplementary file 3 — Additional File 3 Supplementary Appendix Gun types, brands, and models by predominant marketing use classification. [file 40621_2021_305_MOESM3_ESM.docx]

**Supplementary Appendix** Gun types, brands, and models by predominant marketing use classification

| **Brand** | **Model** | **Type** | **Caliber** | **Length (in)** | **Magazine Capacity** | **Predominant Marketing Use** |
| --- | --- | --- | --- | --- | --- | --- |
| Alexander  Alexander | Highlander .50 Beowulf Pistol | Pistol | 50 Beowulf |  | 7 | Tactical |
| Alexander | Highlander 300 AAC Pistol | Pistol | 300 AAC |  | 30 | Tactical |
| Alexander | Highlander 6.5 Grendel Pistol | Pistol | 6.5 Grendel |  | 10 | Tactical |
| Anderson | AM-15 EXT Pistol | Pistol | .300 BLK AAC | 25" | 30 | Tactical |
| Anderson | AM-15 EXT Pistol | Pistol | 5.56 NATO | 24" | 30 | Tactical |
| Beretta | 92A1 | Pistol | 9mm | 8.54 | 17 | Tactical |
| Beretta | 92FS | Pistol | 9mm | 8.54 | 15 | Tactical |
| Beretta | 92FS 22LR | Pistol | 22 LR | 8.54 | 15 | Tactical |
| Beretta | APX | Pistol | 40 S&W | 7.56 | 15 | Tactical |
| Beretta | APX | Pistol | 9mm | 7.56 | 17 | Tactical |
| Beretta | APX Centurion | Pistol | 40 S&W | 6.97 | 15 | Tactical |
| Beretta | APX Centurion | Pistol | 9mm | 6.97 | 15 | Tactical |
| Beretta | APX Combat | Pistol | 9mm | 8.19 | 17 | Tactical |
| Beretta | APX Compact | Pistol | 40 S&W | 6.97 | 11 | Tactical |
| Beretta | APX Compact | Pistol | 9mm | 6.97 | 13 | Tactical |
| Beretta | APX RDO | Pistol | 9mm | 7.56 | 17 | Tactical |
| Beretta | APX Tactical | Pistol | 9mm | 7.56 | 17 | Tactical |
| Beretta | M9A1 | Pistol | 9mm | 8.54 | 17 | Tactical |
| Beretta | M9A1 Compact | Pistol | 9mm | 7.76 | 13 | Tactical |
| Beretta | M9A3 | Pistol | 9mm | 8.66 | 17 | Tactical |
| Beretta | Px4 Storm Compact Inox | Pistol | 9mm | 6.81 | 15 | Tactical |
| Beretta | Px4 Storm Compact Type F | Pistol | 40 S&W | 6.81 | 12 | Tactical |
| Beretta | Px4 Storm Compact Type F | Pistol | 9mm | 6.81 | 15 | Tactical |
| Beretta | Px4 Storm Deluxe | Pistol | 9mm | 7.56 | 17 | Tactical |
| Beretta | Px4 Storm Inox | Pistol | 40 S&W | 7.56 | 14 | Tactical |
| Beretta | Px4 Storm Inox | Pistol | 9mm | 7.68 | 17 | Tactical |
| Beretta | Px4 Storm Special Duty | Pistol | 45 ACP | 8.27 | 10 | Tactical |
| Beretta | Px4 Storm SubCompact Type F | Pistol | 9mm | 6.22 | 13 | Tactical |
| Beretta | Px4 Storm Type F | Pistol | 40 S&W | 7.56 | 14 | Tactical |
| Beretta | Px4 Storm Type F | Pistol | 45 ACP | 7.68 | 10 | Tactical |
| Beretta | Px4 Storm Type F | Pistol | 9mm | 7.56 | 17 | Tactical |
| Beretta | Px4 Storm Type G | Pistol | 40 S&W | 7.56 | 14 | Tactical |
| Beretta | Px4 Storm Type G | Pistol | 9mm | 7.56 | 17 | Tactical |
| Browning | 1911 22 A1 Compact | Pistol | 22 LR | 3-5/8 | 10 | Recreation |
| Browning | 1911 22 A1 Compact, desert tan | Pistol | 22 LR | 3-5/8 | 10 | Recreation |
| Browning | 1911 22 A1 Full Size | Pistol | 22 LR | 4-1/4 | 10 | Recreation |
| Browning | 1911 22 A1 Full size, desert tan | Pistol | 22 LR | 4-1/4 | 10 | Recreation |
| Browning | 1911 22 Compact | Pistol | 22 LR | 3-5/8 | 10 | Recreation |
| Browning | 1911 22 Compact w rail | Pistol | 22 LR | 3-5/8 | 10 | Recreation |
| Browning | 1911 22 Full size | Pistol | 22 LR | 4-1/4 | 10 | Recreation |
| Browning | 1911 22 Full size w rail | Pistol | 22 LR | 4-1/4 | 10 | Recreation |
| Browning | 1911 22 Gray compact | Pistol | 22 LR | 3-5/8 | 10 | Recreation |
| Browning | 1911 22 Gray compact w rail | Pistol | 22 LR | 3-5/8 | 10 | Recreation |
| Browning | 1911 22 Gray full size | Pistol | 22 LR | 4-1/4 | 10 | Recreation |
| Browning | 1911 22 Gray full size w rail | Pistol | 22 LR | 4-1/4 | 10 | Recreation |
| Browning | 1911 22 Medallion, compact | Pistol | 22 LR | 3-5/8 | 10 | Recreation |
| Browning | 1911 22 Medallion, full size | Pistol | 22 LR | 4-1/4 | 10 | Recreation |
| Browning | 1911 22 SR, compact | Pistol | 22 LR | 4-1/4 | 10 | Recreation |
| Browning | 1911 22 SR, full size | Pistol | 22 LR | 4-7/8 | 10 | Recreation |
| Browning | 1911 380 compact | Pistol | 380 ACP | 3-5/8 | 8 | Recreation |
| Browning | 1911 380 full size | Pistol | 380 ACP | 4-1/4 | 8 | Recreation |
| Browning | 1911 380 medallion pro compact | Pistol | 380 ACP | 3-5/8 | 8 | Recreation |
| Browning | 1911 380 medallion pro full size | Pistol | 380 ACP | 4-1/4 | 8 | Recreation |
| Browning | 1911 380 pro compact | Pistol | 380 ACP | 3-5/8 | 8 | Recreation |
| Browning | 1911 380 pro compact w rail | Pistol | 380 ACP | 3-5/8 | 8 | Recreation |
| Browning | 1911 380 pro full size | Pistol | 380 ACP | 4-1/4 | 8 | Recreation |
| Browning | 1911 380 pro full size w rail | Pistol | 380 ACP | 4-1/4 | 8 | Recreation |
| Browning | 1911 380 pro stainless compact | Pistol | 380 ACP | 3-5/8 | 8 | Recreation |
| Browning | 1911 380 pro stainless compact w rail | Pistol | 380 ACP | 3-5/8 | 8 | Recreation |
| Browning | 1911 380 pro stainless full size | Pistol | 380 ACP | 4-1/4 | 8 | Recreation |
| Browning | 1911 380 pro stainless full size w rail | Pistol | 380 ACP | 4-1/4 | 8 | Recreation |
| Browning | Buck Mark Camper Stainless UFX | Pistol | 22 LR | 5-1/2 | 10 | Recreation |
| Browning | Buck Mark Camper Stainless URX | Pistol | 22 LR | 5-1/2 | 10 | Recreation |
| Browning | Buck Mark Camper Standard URX | Pistol | 22 LR | 5-1/2 | 10 | Recreation |
| Browning | Buck Mark Camper UFX | Pistol | 22 LR | 5-1/2 | 10 | Recreation |
| Browning | Buck Mark Challenge Rosewood | Pistol | 22 LR | 5-1/2 | 10 | Recreation |
| Browning | Buck Mark Contour Stainless URX | Pistol | 22 LR | 5-1/2 | 10 | Recreation |
| Browning | Buck Mark Contour Stainless URX | Pistol | 22 LR | 7-1/4 | 10 | Recreation |
| Browning | Buck Mark Contour URX | Pistol | 22 LR | 5-1/2 | 10 | Recreation |
| Browning | Buck Mark Contour URX | Pistol | 22 LR | 7-1/4 | 10 | Recreation |
| Browning | Buck Mark Field Target | Pistol | 22 LR | 5-1/2 | 10 | Recreation |
| Browning | Buck Mark Field Target SR | Pistol | 22 LR | 5-1/2 | 10 | Recreation |
| Browning | Buck Mark Hunter | Pistol | 22 LR | 7-1/4 | 10 | Recreation |
| Browning | Buck Mark Lite Gray URX | Pistol | 22 LR | 7-1/4 | 10 | Recreation |
| Browning | Buck Mark Lite Gray URX | Pistol | 22LR | 5-1/2 | 10 | Recreation |
| Browning | Buck Mark lite Green URX | Pistol | 22 LR | 5-1/2 | 10 | Recreation |
| Browning | Buck Mark plus Camper UFX SR | Pistol | 22LR | 6 | 10 | Recreation |
| Browning | Buck Mark plus lite flute UFX SR | Pistol | 22 LR | 6 | 10 | Recreation |
| Browning | Buck Mark plus practical URX | Pistol | 22LR | 5-1/2 | 10 | Recreation |
| Browning | Buck Mark plus rosewood UDX | Pistol | 22LR | 5-1/2 | 10 | Recreation |
| Browning | Buck Mark plus stainless UDX | Pistol | 22LR | 5-1/2 | 10 | Recreation |
| Browning | Buck Mark plus UDX | Pistol | 22LR | 5-1/2 | 10 | Recreation |
| Browning | Buck Mark Standard Micro URX | Pistol | 22 LR | 5-1/2 | 10 | Recreation |
| Browning | Buck Mark Standard Stainless URX | Pistol | 22 LR | 5-1/2 | 10 | Recreation |
| Bushmaster | 11" Squaredrop Pistol | Pistol | 5.56 NATO | 26.75 | 30 | Tactical |
| Bushmaster | 7" Squaredrop Pistol | Pistol | 5.56 NATO | 23.75 | 30 | Tactical |
| Bushmaster | 9" Squaredrop Pistol | Pistol | 300 BLK | 25.25 | 30 | Tactical |
| Century Arms | C39v2 Pistol | Pistol | 7.62x39mm | 20.50 | 30 | Tactical |
| Century Arms | Draco Pistol | Pistol | 7.62x39mm | 21.50 | 30 | Tactical |
| Century Arms | Micro Draco Pistol | Pistol | 7.62x39mm | 14.50 | 30 | Tactical |
| Century Arms | Mini Draco Pistol | Pistol | 7.62x39mm | 17.50 | 30 | Tactical |
| Century Arms | PAP M92 | Pistol | 7.62x39mm | 19.30 | 30 | Tactical |
| Century Arms | RAS-47 Pistol | Pistol | 7.62x39mm | 20.50 | 30 | Tactical |
| Christensen Arms | 1911 Classic Series (includes G5, G5-TI, G5-TR, C4, and C4-TI) | Pistol | 45 ACP |  | 7 | Self-Defense |
| Christensen Arms | 1911 Classic Series (includes G5, G5-TI, G5-TR, C4, and C4-TI) | Pistol | 9mm |  | 9 | Self-Defense |
| Christensen Arms | 1911 Series (includes A4, A5, and A5-TR) | Pistol | 45 ACP | 7.75 | 7 | Self-Defense |
| Christensen Arms | 1911 Series (includes A4, A5, and A5-TR) | Pistol | 9mm | 7.75 | 9 | Self-Defense |
| Colt | 570 Government | Pistol | .45 ACP | 8.5" | 7 | Self-Defense |
| Colt | Bright Stainless | Pistol | .38 Super | 8.5" | 9 | Self-Defense |
| Colt | Bright Stainless (45ACP) | Pistol | .45 ACP | 8.5" | 9 | Self-Defense |
| Colt | Clt Gold Cup LIte 9MM | Pistol | 9MM | 8.5" | 9 | Self-Defense |
| Colt | Colt 1903 Blued | Pistol | .32 ACP | 6.75" | 8 | Self-Defense |
| Colt | Colt Gold Cup Lite | Pistol | .38 Super | 8.5" | 9 | Self-Defense |
| Colt | Combat Commander (45 ACP) | Pistol | .45 ACP | 7.75" | 8 | Self-Defense |
| Colt | Combat Commander (9MM) | Pistol | 9MM | 7.75" | 9 | Self-Defense |
| Colt | Combat Elite Commander | Pistol | .45 ACP | 7.75" | 8 | Self-Defense |
| Colt | Combat Elite Commander (9MM) | Pistol | 9MM | 7.75" | 9 | Self-Defense |
| Colt | Combat Elite Defender | Pistol | .45 ACP | 6.75" | 7 | Self-Defense |
| Colt | Combat Elite Defender (9MM) | Pistol | 9MM | 6.75" | 9 | Self-Defense |
| Colt | Combat Elite Govt | Pistol | .45 ACP | 8.5" | 8 | Self-Defense |
| Colt | Combat Elite Govt (9MM) | Pistol | 9MM | 8.5" | 9 | Self-Defense |
| Colt | Combat Unit Rail (45 ACP) | Pistol | .45 ACP | 8.5" | 8 | Self-Defense |
| Colt | Competition | Pistol | .45 ACP | 8.5" | 8 | Self-Defense |
| Colt | Competition SS (38S) | Pistol | .38 Super | 8.5" | 9 | Self-Defense |
| Colt | Competition SS (45ACP) | Pistol | .45 ACP | 8.5" | 8 | Self-Defense |
| Colt | Custom Shop CQB (45ACP) | Pistol | .45 ACP | 8.5" | 8 | Self-Defense |
| Colt | Defender | Pistol | 9MM | 6.75" | 7 | Self-Defense |
| Colt | Defender Gray (45ACP) | Pistol | .45 ACP | 8.5" | 7 | Self-Defense |
| Colt | Defender Gray (9MM) | Pistol | 9MM | 6.75" | 7 | Self-Defense |
| Colt | Defender SS | Pistol | .45 ACP | 6.75" | 7 | Self-Defense |
| Colt | Delta Elite Rail (10MM) | Pistol | .41 Magnum | 8.5" | 8 | Self-Defense |
| Colt | Delta Elite TT | Pistol | 10MM | 8.5" | 8 | Self-Defense |
| Colt | Gold Cup Lite 45 acp SS 5 | Pistol | .45 ACP | 8.5" | 8 | Self-Defense |
| Colt | Gold Cup NM | Pistol | .45 ACP | 8.5" | 8 | Self-Defense |
| Colt | Gold Cup Trophy (45 ACP) | Pistol | .45 ACP | 8.5" | 8 | Self-Defense |
| Colt | Government 45 acp Blck 5 | Pistol | .45 ACP | 8.5" | 8 | Self-Defense |
| Colt | Government Model | Pistol | .45 ACP | 8.5" | 7 | Self-Defense |
| Colt | LW Commander (45 ACP) | Pistol | .45 ACP | 7.75" | 8 | Self-Defense |
| Colt | M45A1 Marine Pistol | Pistol | .45 ACP | 8.5" | 7 | Self-Defense |
| Colt | Mustang Lite | Pistol | .380 ACP | 5.5" | 6 | Self-Defense |
| Colt | Mustang Pocketlite | Pistol | .380 ACP | 5.5" | 6 | Self-Defense |
| Colt | Rail Gun SS (45 ACP) | Pistol | .45 ACP | 8.5" | 8 | Self-Defense |
| Colt | S70 Government | Pistol | .45 ACP | 8.5" | 7 | Self-Defense |
| Colt | Series 70 Government SS | Pistol | .45 ACP | 8.5" | 7 | Self-Defense |
| Colt | Special Combat Govt | Pistol | .45 ACP | 8.5" | 8 | Self-Defense |
| Colt | WC Commander SS (45ACP) | Pistol | .45 ACP | 7.75" | 8 | Self-Defense |
| Colt | WC Government 1911 | Pistol | .45 ACP | 8.5" | 7 | Self-Defense |
| Colt | WC LW Commander (45ACP) | Pistol | .45 ACP | 7.75" | 8 | Self-Defense |
| Colt | WC LW Commander (9MM) | Pistol | 9MM | 7.75" | 9 | Self-Defense |
| Colt | Wiley Clapp CCO | Pistol | .45 ACP | 7.75" | 6 | Self-Defense |
| FN America | FN 509 | Pistol | 9mm | 7.40 | 17 | Tactical |
| FN America | FN 509 Midsize | Pistol | 9mm | 7.40 | 15 | Tactical |
| FN America | FN 509 Tactical | Pistol | 9mm | 7.90 | 24 | Tactical |
| FN America | FN Five-seveN | Pistol | 5.7x28mm | 8.20 | 20 | Tactical |
| FN America | FN15 Pistol .300 BLK | Pistol | 7.62x35mm / 300 BLK | 28.63 | 30 | Tactical |
| FN America | FN15 Pistol 5.56 | Pistol | 5.56 NATO | 26.25 | 30 | Tactical |
| FN America | FNX-40 | Pistol | 40 S&W |  | 14 | Tactical |
| FN America | FNX-45 | Pistol | 45 ACP | 7.90 | 15 | Tactical |
| FN America | FNX-45 Tactical | Pistol | 45 ACP | 8.60 | 15 | Tactical |
| FN America | FNX-9 | Pistol | 9mm |  | 17 | Tactical |
| Glock | G17 | Pistol | 9mm | 7.95 | 17 | Self-Defense |
| Glock | G17L | Pistol | 9mm | 9.53 | 17 | Self-Defense |
| Glock | G19 | Pistol | 9mm | 7.28 | 15 | Self-Defense |
| Glock | G19X | Pistol | 9mm | 7.44 | 17 | Self-Defense |
| Glock | G20 | Pistol | 10mm Auto | 8.07 | 15 | Self-Defense |
| Glock | G21 | Pistol | 45 Auto | 8.07 | 13 | Self-Defense |
| Glock | G23 | Pistol | 40 S&W | 7.28 | 13 | Self-Defense |
| Glock | G24 | Pistol | 40 S&W | 9.57 | 15 | Self-Defense |
| Glock | G26 | Pistol | 9mm | 6.42 | 10 | Self-Defense |
| Glock | G28 | Pistol | 380 Auto | 6.50 | 10 | Self-Defense |
| Glock | G29 | Pistol | 10mm Auto | 6.97 | 10 | Self-Defense |
| Glock | G30 | Pistol | 45 Auto | 6.97 | 10 | Self-Defense |
| Glock | G31 | Pistol | 357 Sig | 7.95 | 15 | Self-Defense |
| Glock | G33 | Pistol | 357 Sig | 6.42 | 9 | Self-Defense |
| Glock | G35 | Pistol | 40 S&W | 8.74 | 15 | Recreation |
| Glock | G36 | Pistol | 45 Auto | 6.97 | 6 | Self-Defense |
| Glock | G37 | Pistol | 45 GAP | 8.03 | 10 | Self-Defense |
| Glock | G38 | Pistol | 45 GAP | 7.36 | 8 | Self-Defense |
| Glock | G39 | Pistol | 45 GAP | 6.50 | 6 | Self-Defense |
| Glock | G40 | Pistol | 10mm Auto | 9.49 | 15 | Self-Defense |
| Glock | G41 | Pistol | 45 Auto | 8.78 | 13 | Recreation |
| Glock | G42 | Pistol | 380 Auto | 5.94 | 6 | Self-Defense |
| Glock | G43 | Pistol | 9mm | 6.26 | 6 | Self-Defense |
| Heckler & Koch | HK416 | Pistol | 22 LR | 17.20 | 20 | Recreation |
| Heckler & Koch | HK45 | Pistol | 45 ACP | 8.03 | 10 | Self-Defense |
| Heckler & Koch | HK45 Compact | Pistol | 45 ACP | 7.24 | 10 | Self-Defense |
| Heckler & Koch | HK45 Compact Tactical | Pistol | 45 ACP | 7.91 | 10 | Tactical |
| Heckler & Koch | HK45 Tactical | Pistol | 45 ACP | 8.50 | 10 | Tactical |
| Heckler & Koch | MARK 23 | Pistol | 45 Auto | 9.65 | 12 | Self-Defense |
| Heckler & Koch | P2000 | Pistol | 40 S&W | 6.85 | 10 | Self-Defense |
| Heckler & Koch | P2000 | Pistol | 9mm | 6.85 | 13 | Self-Defense |
| Heckler & Koch | P2000SK | Pistol | 40 S&W | 6.40 | 9 | Self-Defense |
| Heckler & Koch | P2000SK | Pistol | 9mm | 6.40 | 10 | Self-Defense |
| Heckler & Koch | P30 | Pistol | 40 S&W | 7.12 | 13 | Self-Defense |
| Heckler & Koch | P30 | Pistol | 9mm | 7.12 | 15 | Self-Defense |
| Heckler & Koch | P30L | Pistol | 40 S&W | 7.71 | 13 | Self-Defense |
| Heckler & Koch | P30L | Pistol | 9mm | 7.71 | 15 | Self-Defense |
| Heckler & Koch | P30SK | Pistol | 9mm | 6.42 | 11 | Self-Defense |
| Heckler & Koch | SP5K | Pistol | 9mm | 13.90 | 30 | Tactical |
| Heckler & Koch | USP | Pistol | 40 S&W | 7.68 | 13 | Self-Defense |
| Heckler & Koch | USP | Pistol | 45 Auto | 7.91 | 12 | Self-Defense |
| Heckler & Koch | USP | Pistol | 9mm | 7.68 | 15 | Self-Defense |
| Heckler & Koch | USP Compact | Pistol | 40 S&W | 6.81 | 12 | Self-Defense |
| Heckler & Koch | USP Compact | Pistol | 45 Auto | 7.09 | 9 | Self-Defense |
| Heckler & Koch | USP Compact | Pistol | 9mm | 6.81 | 13 | Self-Defense |
| Heckler & Koch | USP Tactical | Pistol | 45 Auto | 8.64 | 12 | Tactical |
| Heckler & Koch | USP Tactical | Pistol | 9mm | 7.94 | 15 | Tactical |
| Heckler & Koch | VP40 | Pistol | 40 S&W | 7.34 | 13 | Self-Defense |
| Heckler & Koch | VP9 | Pistol | 9mm | 7.34 | 15 | Self-Defense |
| Heckler & Koch | VP9 Tactical | Pistol | 9mm | 7.95 | 15 | Tactical |
| Heckler & Koch | VP9SK | Pistol | 9mm | 6.61 | 10 | Self-Defense |
| Hi-Point Firearms | 380ACP | Pistol | 380ACP | 6.75" | 8 (10 Avail) | Self-Defense |
| Hi-Point Firearms | C-9 | Pistol | 9MM | 6.75" | 8 (10 also) | Self-Defense |
| Hi-Point Firearms | JCP 40 SW | Pistol | 40 S and W | 7.75" | 10 | Self-Defense |
| Hi-Point Firearms | JHP45ACP | Pistol | 45ACP | 7.75" | 9 | Self-Defense |
| Kel-Tec | P-11 | Pistol | 9mm | 5.60 | 10 | Self-Defense |
| Kel-Tec | P-32 | Pistol | 32 ACP | 5.10 | 7 | Self-Defense |
| Kel-Tec | P-3AT | Pistol | 380 ACP | 5.20 | 6 | Self-Defense |
| Kel-Tec | PF-9 | Pistol | 9mm | 5.85 | 7 | Self-Defense |
| Kel-Tec | PLR-16 | Pistol | 5.56mm NATO / 223 Rem | 18.50 | 10 | Recreation |
| Kel-Tec | PLR-22 | Pistol | 22 LR | 18.50 | 26 | Recreation |
| Kel-Tec | PMR-30 | Pistol | 22WMR | 7.90 | 30 | Recreation |
| Kimber | Aegis Elite Custom | Pistol | 9mm / 45 ACP | 8.70 | 9 | Self-Defense |
| Kimber | Aegis Elite Pro | Pistol | 9mm / 45 ACP | 7.70 | 9 | Self-Defense |
| Kimber | Aegis Elite Ultra | Pistol | 9mm / 45 ACP | 6.80 | 8 | Self-Defense |
| Kimber | Amethyst Ultra II | Pistol | 9mm / 45 ACP | 6.80 | 8 | Self-Defense |
| Kimber | Camp Guard 10 | Pistol | 10mm | 8.70 | 8 | Self-Defense |
| Kimber | Classic Carry Pro | Pistol | 45 ACP | 7.70 | 8 | Self-Defense |
| Kimber | Custom CDP | Pistol | 45 ACP | 8.70 | 7 | Self-Defense |
| Kimber | Custom Covert | Pistol | 45 ACP | 8.70 | 7 | Self-Defense |
| Kimber | Custom Crimson Carry II | Pistol | 45 ACP | 8.70 | 8 | Self-Defense |
| Kimber | Custom II | Pistol | 9mm / 45 ACP | 8.70 | 7 | Self-Defense |
| Kimber | Custom TLE II | Pistol | 45 ACP / 10mm | 8.70 | 8 | Self-Defense |
| Kimber | Custom TLE/RL II | Pistol | 45 ACP / 10mm | 8.70 | 8 | Self-Defense |
| Kimber | Custom TLE/RL II (EM) | Pistol | 45 ACP | 8.70 | 7 | Self-Defense |
| Kimber | Custom TLE/RL II (TFS) | Pistol | 9mm / 45 ACP | 9.19 | 9 | Self-Defense |
| Kimber | Desert Warrior | Pistol | 45 ACP | 8.70 | 7 | Self-Defense |
| Kimber | Desert Warrior (TFS) | Pistol | 45 ACP | 9.19 | 7 | Self-Defense |
| Kimber | Eclipse Custom | Pistol | 45 ACP / 10mm | 8.70 | 8 | Self-Defense |
| Kimber | Eclipse Pro | Pistol | 45 ACP | 7.70 | 8 | Self-Defense |
| Kimber | Eclipse Target | Pistol | 38 Super / 45 ACP | 8.70 | 8 | Self-Defense |
| Kimber | Gold Combat II | Pistol | 45 ACP | 8.70 | 8 | Self-Defense |
| Kimber | Gold Combat RL II | Pistol | 45 ACP | 8.70 | 8 | Self-Defense |
| Kimber | Gold Combat Stainless II | Pistol | 45 ACP | 8.70 | 8 | Self-Defense |
| Kimber | Gold Match II | Pistol | 45 ACP | 8.70 | 8 | Recreation |
| Kimber | Grand Raptor II | Pistol | 45 ACP | 8.70 | 8 | Self-Defense |
| Kimber | Hero Custom | Pistol | 45 ACP | 8.70 | 8 | Self-Defense |
| Kimber | KHX Custom | Pistol | 9mm / 45 ACP | 8.70 | 9 | Self-Defense |
| Kimber | KHX Pro | Pistol | 9mm / 45 ACP | 7.70 | 9 | Self-Defense |
| Kimber | KHX Ultra | Pistol | 9mm / 45 ACP | 6.80 | 8 | Self-Defense |
| Kimber | Master Carry Custom | Pistol | 45 ACP | 8.70 | 8 | Self-Defense |
| Kimber | Master Carry Pro | Pistol | 9mm / 45 ACP | 7.70 | 9 | Self-Defense |
| Kimber | Master Carry Ultra | Pistol | 45 ACP | 6.80 | 7 | Self-Defense |
| Kimber | Micro | Pistol | 380 ACP | 5.60 | 7 | Self-Defense |
| Kimber | Micro 9 | Pistol | 9mm | 6.10 | 7 | Self-Defense |
| Kimber | Pro Carry II | Pistol | 9mm / 45 ACP | 7.70 | 9 | Self-Defense |
| Kimber | Pro CDP | Pistol | 9mm / 45 ACP | 7.70 | 9 | Self-Defense |
| Kimber | Pro Covert | Pistol | 45 ACP | 7.70 | 7 | Self-Defense |
| Kimber | Pro Crimson Carry II | Pistol | 45 ACP | 7.70 | 8 | Self-Defense |
| Kimber | Pro Raptor II | Pistol | 45 ACP | 7.70 | 8 | Self-Defense |
| Kimber | Pro TLE II | Pistol | 45 ACP | 7.70 | 7 | Self-Defense |
| Kimber | Pro TLE/RL II | Pistol | 45 ACP | 7.70 | 7 | Self-Defense |
| Kimber | Raptor II | Pistol | 45 ACP | 8.70 | 8 | Self-Defense |
| Kimber | Rimfire Compact Conversion Kit | Pistol | 22 LR | 7.70 | 10 | Recreation |
| Kimber | Rimfire Target Conversion Kit | Pistol | 22LR | 8.70 | 10 | Recreation |
| Kimber | Rose Gold Ultra II | Pistol | 9mm / 45 ACP | 6.80 | 8 | Self-Defense |
| Kimber | Royal II | Pistol | 45 ACP | 8.70 | 7 | Self-Defense |
| Kimber | Sapphire Pro II | Pistol | 9mm | 7.70 | 9 | Self-Defense |
| Kimber | Sapphire Ultra II | Pistol | 9mm / 45 ACP | 6.80 | 8 | Self-Defense |
| Kimber | Stainless Gold Match II | Pistol | 45 ACP | 8.70 | 8 | Recreation |
| Kimber | Stainless II | Pistol | 9mm / 45 ACP | 8.70 | 9 | Self-Defense |
| Kimber | Stainless Pro Carry II | Pistol | 9mm / 45 ACP | 7.70 | 9 | Self-Defense |
| Kimber | Stainless Pro Raptor II | Pistol | 9mm / 45 ACP | 7.70 | 8 | Self-Defense |
| Kimber | Stainless Pro TLE II | Pistol | 45 ACP | 7.70 | 7 | Self-Defense |
| Kimber | Stainless Pro TLE/RL II | Pistol | 45 ACP | 7.70 | 7 | Self-Defense |
| Kimber | Stainless Raptor II | Pistol | 9mm / 45 ACP | 8.70 | 8 | Self-Defense |
| Kimber | Stainless Target (LS) | Pistol | 45 ACP / 10mm | 9.70 | 8 | Self-Defense |
| Kimber | Stainless Target II | Pistol | 9mm / 45 ACP | 8.70 | 9 | Self-Defense |
| Kimber | Stainless TLE II | Pistol | 45 ACP | 8.70 | 7 | Self-Defense |
| Kimber | Stainless TLE/RL II | Pistol | 45 ACP | 8.70 | 7 | Self-Defense |
| Kimber | Stainless Ultra Carry II | Pistol | 9mm / 45 ACP | 6.80 | 8 | Self-Defense |
| Kimber | Stainless Ultra Raptor II | Pistol | 9mm / 45 ACP | 6.80 | 8 | Self-Defense |
| Kimber | Stainless Ultra TLE II | Pistol | 45 ACP | 6.80 | 7 | Self-Defense |
| Kimber | Super Carry Custom | Pistol | 45 ACP | 8.70 | 8 | Self-Defense |
| Kimber | Super Carry Pro | Pistol | 45 ACP | 7.70 | 8 | Self-Defense |
| Kimber | Super Carry Ultra | Pistol | 45 ACP | 6.80 | 7 | Self-Defense |
| Kimber | Super Carry Ultra+ | Pistol | 45 ACP | 6.80 | 8 | Self-Defense |
| Kimber | Super Jagare | Pistol | 10mm | 9.70 | 8 | Self-Defense |
| Kimber | Super Match II | Pistol | 45 ACP | 8.70 | 8 | Self-Defense |
| Kimber | Team Match II | Pistol | 9mm / 45 ACP | 8.70 | 9 | Self-Defense |
| Kimber | Ultra Carry II | Pistol | 9mm / 45 ACP | 6.80 | 7 | Self-Defense |
| Kimber | Ultra CDP | Pistol | 9mm / 45 ACP | 6.80 | 8 | Self-Defense |
| Kimber | Ultra Covert | Pistol | 45 ACP | 6.80 | 7 | Self-Defense |
| Kimber | Ultra Crimson Carry II | Pistol | 45 ACP | 6.80 | 7 | Self-Defense |
| Kimber | Ultra Raptor II | Pistol | 45 ACP | 6.80 | 7 | Self-Defense |
| Kimber | Ultra RCP II | Pistol | 45 ACP | 6.80 | 7 | Self-Defense |
| Kimber | Ultra TLE II | Pistol | 45 ACP | 6.80 | 7 | Self-Defense |
| Kimber | Ultra+ CDP | Pistol | 45 ACP | 6.80 | 7 | Self-Defense |
| Kimber | Warrior | Pistol | 45 ACP | 8.70 | 7 | Self-Defense |
| Kimber | Warrior SOC | Pistol | 45 ACP | 8.70 | 7 | Self-Defense |
| Kimber | Warrior SOC (TFS) | Pistol | 45 ACP | 9.19 | 7 | Self-Defense |
| Mossberg | MC1sc | Pistol | 9mm | 6.25 | 7 | Self-Defense |
| Palmetto State Armory | 1911 Pistol | Pistol | 45 ACP | 8.75 | 7 | Recreation |
| Palmetto State Armory | AK-V | Pistol | 9mm |  | 35 | Tactical |
| Palmetto State Armory | KS-47 Pistol | Pistol | 7.62x39mm | 26.50 | 30 | Tactical |
| Palmetto State Armory | PA-15 Pistol | Pistol | 5.56 NATO / 6.5 Grendel / 300 BLK | 26.00 | 30 | Tactical |
| Palmetto State Armory | PA-9 Pistol | Pistol | 9mm |  | 31 | Tactical |
| Remington | Model 1911 R1 | Pistol | 45 Auto | 8.50 | 7 | Self-Defense |
| Remington | Model 1911 R1 Commander | Pistol | 45 Auto | 7.50 | 7 | Self-Defense |
| Remington | Model 1911 R1 Enhanced | Pistol | 9mm / 45 Auto | 8.50 | 9 | Self-Defense |
| Remington | Model 1911 R1 Enhanced Commander | Pistol | 45 Auto | 7.75 | 8 | Self-Defense |
| Remington | Model 1911 R1 Enhanced Crimson Trace | Pistol | 45 Auto | 8.50 | 8 | Self-Defense |
| Remington | Model 1911 R1 Enhanced Double Stack | Pistol | 45 Auto | 8.50 | 15 | Self-Defense |
| Remington | Model 1911 R1 Enhanced Stainless | Pistol | 45 Auto | 8.50 | 8 | Self-Defense |
| Remington | Model 1911 R1 Enhanced Threaded | Pistol | 45 Auto | 9.25 | 8 | Self-Defense |
| Remington | Model 1911 R1 Hunter | Pistol | 10mm | 9.50 | 8 | Recreation |
| Remington | Model 1911 R1 Limited | Pistol | 9mm / 40 S&W | 8.50 | 9 | Recreation |
| Remington | Model 1911 R1 Limited Double Stack | Pistol | 9mm / 40 S&W / 45 Auto | 8.50 | 19 | Self-Defense |
| Remington | Model 1911 R1 Recon | Pistol | 9mm / 45 Auto | 8.50 | 18 | Tactical |
| Remington | Model 1911 R1 Stainless | Pistol | 45 Auto | 8.50 | 7 | Self-Defense |
| Remington | Model 1911 R1 Tactical Double Stack | Pistol | 45 Auto | 8.50 | 15 | Tactical |
| Remington | Model 1911 R1 Tactical Single Stack | Pistol | 45 Auto | 8.50 | 8 | Tactical |
| Remington | Model 1911 R1 Tomasie | Pistol | 40 S&W | 8.50 | 18 | Recreation |
| Remington | Model 1911 R1 Ultralight Commander | Pistol | 45 Auto | 7.75 | 8 | Self-Defense |
| Remington | Model 1911 R1 Ultralight Executive | Pistol | 45 Auto | 7.00 | 7 | Self-Defense |
| Remington | R51 | Pistol | 9mm | 6.63 | 7 | Self-Defense |
| Remington | RM380 | Pistol | 380 Auto | 5.27 | 6 | Self-Defense |
| Remington | RP45 | Pistol | 45 Auto | 7.91 | 15 | Self-Defense |
| Remington | RP45 Restricted | Pistol | 45 Auto | 7.91 | 10 | Self-Defense |
| Remington | RP45 Titanium Night Sights | Pistol | 45 Auto | 7.91 | 15 | Self-Defense |
| Remington | RP9 | Pistol | 9mm | 7.91 | 18 | Self-Defense |
| Remington | RP9 Restricted | Pistol | 9mm | 7.91 | 10 | Self-Defense |
| Ruger | 22 Charger | Pistol | 22 LR | 19.25 | 15 | Recreation |
| Ruger | 22 Charger | Pistol | 22 LR | 19.25 | 15 | Recreation |
| Ruger | EC9 | Pistol | 9mm | 6.00 | 7 | Self-Defense |
| Ruger | LC380 | Pistol | 380 Auto | 6.00 | 7 | Self-Defense |
| Ruger | LC380CA | Pistol | 380 Auto | 6.00 | 7 | Self-Defense |
| Ruger | LCP | Pistol | 380 Auto | 5.16 | 6 | Self-Defense |
| Ruger | LCP | Pistol | 380 Auto | 5.16 | 6 | Self-Defense |
| Ruger | LCP | Pistol | 380 Auto | 5.16 | 6 | Self-Defense |
| Ruger | LCP | Pistol | 380 Auto | 5.16 | 6 | Self-Defense |
| Ruger | LCP II | Pistol | 380 Auto | 5.17 | 6 | Self-Defense |
| Ruger | LCP II | Pistol | 380 Auto | 5.17 | 6 | Self-Defense |
| Ruger | LCP II | Pistol | 380 Auto | 5.17 | 6 | Self-Defense |
| Ruger | LCP II | Pistol | 380 Auto | 5.17 | 6 | Self-Defense |
| Ruger | Mark IV 22/45 | Pistol | 22 LR | 9.75 | 10 | Recreation |
| Ruger | Mark IV 22/45 Lite | Pistol | 22 LR | 8.40 | 10 | Recreation |
| Ruger | Mark IV 22/45 Lite | Pistol | 22 LR | 8.40 | 10 | Recreation |
| Ruger | Mark IV 22/45 Lite | Pistol | 22 LR | 8.40 | 10 | Recreation |
| Ruger | Mark IV 22/45 Tactical | Pistol | 22 LR | 8.50 | 10 | Tactical |
| Ruger | Mark IV Competition | Pistol | 22 LR | 11.12 | 10 | Recreation |
| Ruger | Mark IV Hunter | Pistol | 22 LR | 11.12 | 10 | Recreation |
| Ruger | Mark IV Hunter | Pistol | 22 LR | 11.12 | 10 | Recreation |
| Ruger | Mark IV Standard | Pistol | 22 LR | 9.00 | 10 | Recreation |
| Ruger | Mark IV Standard | Pistol | 22 LR | 10.25 | 10 | Recreation |
| Ruger | Mark IV Tactical | Pistol | 22 LR | 8.50 | 10 | Tactical |
| Ruger | Mark IV Target | Pistol | 22 LR | 9.75 | 10 | Recreation |
| Ruger | Mark IV Target | Pistol | 22 LR | 9.75 | 10 | Recreation |
| Ruger | Mark IV Target | Pistol | 22 LR | 9.75 | 10 | Recreation |
| Ruger | Mark IV Target | Pistol | 22 LR | 9.75 | 10 | Recreation |
| Ruger | Ruger American Pistol Compact | Pistol | 45 Auto | 7.25 | 10 | Self-Defense |
| Ruger | Ruger American Pistol Compact | Pistol | 45 Auto | 7.25 | 10 | Self-Defense |
| Ruger | Ruger American Pistol Compact | Pistol | 9mm | 6.65 | 17 | Self-Defense |
| Ruger | Ruger American Pistol Compact | Pistol | 9mm | 6.65 | 10 | Self-Defense |
| Ruger | Ruger American Pistol Compact | Pistol | 9mm | 6.65 | 17 | Self-Defense |
| Ruger | Ruger American Pistol Compact | Pistol | 9mm | 6.65 | 10 | Self-Defense |
| Ruger | Ruger American Pistol Compact | Pistol | 9mm | 6.65 | 10 | Self-Defense |
| Ruger | Ruger American Pistol Duty | Pistol | 45 Auto | 8.00 | 10 | Self-Defense |
| Ruger | Ruger American Pistol Duty | Pistol | 45 Auto | 8.00 | 10 | Self-Defense |
| Ruger | Ruger American Pistol Duty | Pistol | 45 Auto | 8.00 | 10 | Self-Defense |
| Ruger | Ruger American Pistol Duty | Pistol | 9mm | 7.50 | 17 | Self-Defense |
| Ruger | Ruger American Pistol Duty | Pistol | 9mm | 7.50 | 10 | Self-Defense |
| Ruger | Ruger American Pistol Duty | Pistol | 9mm | 7.50 | 17 | Self-Defense |
| Ruger | Ruger American Pistol Duty | Pistol | 9mm | 7.50 | 10 | Self-Defense |
| Ruger | Ruger American Pistol Duty | Pistol | 9mm | 7.50 | 10 | Self-Defense |
| Ruger | Security-9 | Pistol | 9mm | 7.24 | 15 | Self-Defense |
| Ruger | Security-9 | Pistol | 9mm | 7.24 | 10 | Self-Defense |
| Ruger | SR1911 | Pistol | 45 Auto | 8.67 | 8 | Self-Defense |
| Ruger | SR1911 | Pistol | 45 Auto | 7.75 | 7 | Self-Defense |
| Ruger | SR1911 Lightweight | Pistol | 45 Auto | 7.75 | 7 | Self-Defense |
| Ruger | SR1911 Lightweight | Pistol | 9mm | 7.75 | 9 | Self-Defense |
| Ruger | SR1911 Officer Style | Pistol | 9mm | 7.25 | 7 | Self-Defense |
| Ruger | SR1911 Target | Pistol | 10mm Auto | 8.67 | 8 | Self-Defense |
| Ruger | SR1911 Target | Pistol | 45 Auto | 8.67 | 8 | Self-Defense |
| Ruger | SR1911 Target | Pistol | 9mm | 8.67 | 9 | Self-Defense |
| Ruger | SR22 | Pistol | 22 LR | 6.40 | 10 | Recreation |
| Ruger | SR22 | Pistol | 22 LR | 6.40 | 10 | Recreation |
| Ruger | SR22 | Pistol | 22 LR | 6.40 | 10 | Recreation |
| Ruger | SR22 | Pistol | 22 LR | 7.40 | 10 | Recreation |
| Ruger | SR22 | Pistol | 22 LR | 6.40 | 10 | Recreation |
| Ruger | SR22 | Pistol | 22 LR | 6.40 | 10 | Recreation |
| Ruger | SR40 | Pistol | 40 S&W | 7.50 | 15 | Self-Defense |
| Ruger | SR40 | Pistol | 40 S&W | 7.50 | 15 | Self-Defense |
| Ruger | SR40 | Pistol | 40 S&W | 7.50 | 10 | Self-Defense |
| Ruger | SR40 | Pistol | 40 S&W | 7.50 | 10 | Self-Defense |
| Ruger | SR40c | Pistol | 40 S&W | 6.85 | 15 | Self-Defense |
| Ruger | SR40c | Pistol | 40 S&W | 6.85 | 15 | Self-Defense |
| Ruger | SR40c | Pistol | 40 S&W | 6.85 | 9 | Self-Defense |
| Ruger | SR40c | Pistol | 40 S&W | 6.85 | 9 | Self-Defense |
| Ruger | SR45 | Pistol | 45 Auto | 8.00 | 10 | Self-Defense |
| Ruger | SR45 | Pistol | 45 Auto | 8.00 | 10 | Self-Defense |
| Ruger | SR9 | Pistol | 9mm | 7.50 | 17 | Self-Defense |
| Ruger | SR9 | Pistol | 9mm | 7.50 | 17 | Self-Defense |
| Ruger | SR9 | Pistol | 9mm | 7.50 | 10 | Self-Defense |
| Ruger | SR9 | Pistol | 9mm | 7.50 | 10 | Self-Defense |
| Ruger | SR9c | Pistol | 9mm | 6.85 | 17 | Self-Defense |
| Ruger | SR9c | Pistol | 9mm | 6.85 | 17 | Self-Defense |
| Ruger | SR9c | Pistol | 9mm | 6.85 | 10 | Self-Defense |
| Ruger | SR9c | Pistol | 9mm | 6.85 | 10 | Self-Defense |
| SCCY Firearms | CPX-1 | Pistol | 9mm | 6.00 | 10 | Self-Defense |
| SCCY Firearms | CPX-2 | Pistol | 9mm | 6.00 | 10 | Self-Defense |
| SCCY Firearms | CPX-3 | Pistol | 380 Auto | 5.70 | 10 | Self-Defense |
| Sig Sauer | 1911 C3 | Pistol | 45 Auto | 7.70 | 7 | Recreation |
| Sig Sauer | 1911 Emperor Scorpion | Pistol | 45 Auto | 8.70 | 8 | Recreation |
| Sig Sauer | 1911 Fastback Nightmare | Pistol | 45 Auto | 8.70 | 8 | Recreation |
| Sig Sauer | 1911 Fastback Nightmare Carry | Pistol | 357 SIG / 45 Auto | 7.70 | 8 | Recreation |
| Sig Sauer | 1911 Match Elite Stainless | Pistol | 9mm | 8.70 | 9 | Recreation |
| Sig Sauer | 1911 Max | Pistol | 9mm / 45 Auto | 8.70 | 9 | Recreation |
| Sig Sauer | 1911 Nickel Rail | Pistol | 45 Auto | 8.70 | 8 | Recreation |
| Sig Sauer | 1911 Select | Pistol | 45 Auto | 8.70 | 8 | Recreation |
| Sig Sauer | 1911 Spartan | Pistol | 45 Auto | 8.70 | 8 | Recreation |
| Sig Sauer | 1911 Stainless Super Target | Pistol | 45 Auto | 8.70 | 8 | Recreation |
| Sig Sauer | 1911 STX | Pistol | 45 Auto | 8.70 | 8 | Recreation |
| Sig Sauer | 1911 Tacops | Pistol | 45 Auto / 10mm | 8.70 | 8 | Recreation |
| Sig Sauer | 1911 Traditional Carry Emperor Scorpion | Pistol | 45 Auto | 7.70 | 8 | Recreation |
| Sig Sauer | 1911 Traditional Ultra Compact Two-Tone | Pistol | 9mm | 6.80 | 8 | Recreation |
| Sig Sauer | 1911 Ultra Compact | Pistol | 45 Auto | 6.80 | 7 | Recreation |
| Sig Sauer | 1911 Ultra Compact Two-Tone | Pistol | 45 Auto | 6.80 | 7 | Recreation |
| Sig Sauer | 1911 We the People | Pistol | 45 Auto | 8.70 | 9 | Recreation |
| Sig Sauer | 1911 XO | Pistol | 45 Auto | 8.70 | 8 | Recreation |
| Sig Sauer | P210 Target | Pistol | 9mm | 8.40 | 8 | Recreation |
| Sig Sauer | P220 ASE | Pistol | 45 Auto | 7.70 | 8 | Self-Defense |
| Sig Sauer | P220 Carry | Pistol | 45 Auto | 7.10 | 8 | Self-Defense |
| Sig Sauer | P220 Hunter | Pistol | 10mm | 7.70 | 8 | Recreation |
| Sig Sauer | P220 Legion | Pistol | 45 Auto | 7.70 | 8 | Self-Defense |
| Sig Sauer | P220 Legion 10mm | Pistol | 10mm | 7.70 | 8 | Self-Defense |
| Sig Sauer | P220 Legion SAO | Pistol | 45 Auto | 7.70 | 8 | Self-Defense |
| Sig Sauer | P220 Nitron | Pistol | 45 Auto | 7.70 | 8 | Self-Defense |
| Sig Sauer | P220 Stainless Elite | Pistol | 45 Auto | 7.70 | 8 | Self-Defense |
| Sig Sauer | P225-A1 Classic | Pistol | 9mm | 6.90 | 8 | Self-Defense |
| Sig Sauer | P225-A1 Classic Two-Tone | Pistol | 9mm | 6.90 | 8 | Self-Defense |
| Sig Sauer | P225-A1 Nitron | Pistol | 9mm | 6.90 | 8 | Self-Defense |
| Sig Sauer | P226 22 | Pistol | 22 LR | 7.70 | 10 | Recreation |
| Sig Sauer | P226 22 LR Version | Pistol | 22 LR | 7.90 | 10 | Recreation |
| Sig Sauer | P226 ASE | Pistol | 9mm | 7.70 | 15 | Self-Defense |
| Sig Sauer | P226 Elite SAO | Pistol | 22 LR | 7.90 | 10 | Self-Defense |
| Sig Sauer | P226 Legion | Pistol | 9mm / 40 S&W / 357 SIG | 7.70 | 15 | Self-Defense |
| Sig Sauer | P226 Legion Rx | Pistol | 9mm | 7.70 | 15 | Self-Defense |
| Sig Sauer | P226 Legion SAO Rx | Pistol | 9mm | 7.90 | 15 | Self-Defense |
| Sig Sauer | P226 MK25 | Pistol | 9mm | 7.70 | 15 | Self-Defense |
| Sig Sauer | P226 Nitron | Pistol | 9mm / 40 S&W | 7.70 | 15 | Self-Defense |
| Sig Sauer | P226 Nitron Rx | Pistol | 9mm | 7.70 | 15 | Self-Defense |
| Sig Sauer | P226 Stainless Elite | Pistol | 9mm | 7.70 | 15 | Self-Defense |
| Sig Sauer | P226 Tacops TB | Pistol | 9mm | 7.70 | 15 | Self-Defense |
| Sig Sauer | P227 Carry SAS Gen2 | Pistol | 45 Auto | 7.10 | 10 | Self-Defense |
| Sig Sauer | P227 Nitron | Pistol | 45 Auto | 7.70 | 10 | Self-Defense |
| Sig Sauer | P227 Nitron Rx | Pistol | 45 Auto | 7.70 | 10 | Self-Defense |
| Sig Sauer | P227 Tacops | Pistol | 45 Auto | 7.70 | 10 | Self-Defense |
| Sig Sauer | P229 Emperor Scorpion | Pistol | 9mm | 7.10 | 15 | Self-Defense |
| Sig Sauer | P229 Legion | Pistol | 9mm | 7.10 | 15 | Self-Defense |
| Sig Sauer | P229 Legion Rx | Pistol | 9mm | 7.10 | 15 | Self-Defense |
| Sig Sauer | P229 M11-A1 | Pistol | 9mm | 7.10 | 15 | Self-Defense |
| Sig Sauer | P229 Nitron | Pistol | 9mm / 40 S&W | 7.10 | 15 | Self-Defense |
| Sig Sauer | P229 Nitron Rx | Pistol | 9mm | 7.10 | 15 | Self-Defense |
| Sig Sauer | P229 Select | Pistol | 9mm | 7.10 | 15 | Self-Defense |
| Sig Sauer | P238 Black Pearl | Pistol | 380 ACP | 5.50 | 7 | Self-Defense |
| Sig Sauer | P238 Blackwood | Pistol | 380 ACP | 5.50 | 7 | Self-Defense |
| Sig Sauer | P238 BRG | Pistol | 380 ACP | 5.50 | 7 | Self-Defense |
| Sig Sauer | P238 Desert | Pistol | 380 ACP | 5.50 | 7 | Self-Defense |
| Sig Sauer | P238 Emperor Scorpion | Pistol | 380 ACP | 5.50 | 7 | Self-Defense |
| Sig Sauer | P238 Nightmare | Pistol | 380 ACP | 5.50 | 7 | Self-Defense |
| Sig Sauer | P238 Nitron | Pistol | 380 ACP | 5.50 | 7 | Self-Defense |
| Sig Sauer | P238 Rainbow | Pistol | 380 ACP | 5.50 | 7 | Self-Defense |
| Sig Sauer | P238 Rose Gold | Pistol | 380 ACP | 5.50 | 7 | Self-Defense |
| Sig Sauer | P238 Rosewood | Pistol | 380 ACP | 5.50 | 7 | Self-Defense |
| Sig Sauer | P238 SAS | Pistol | 380 ACP | 5.50 | 7 | Self-Defense |
| Sig Sauer | P238 Select | Pistol | 380 ACP | 5.50 | 7 | Self-Defense |
| Sig Sauer | P238 We the People | Pistol | 380 ACP | 5.50 | 7 | Self-Defense |
| Sig Sauer | P320 Carry | Pistol | 9mm / 357 SIG | 7.20 | 21 | Self-Defense |
| Sig Sauer | P320 Carry Tacops | Pistol | 9mm | 7.20 | 21 | Self-Defense |
| Sig Sauer | P320 Compact | Pistol | 9mm / 40 S&W / 45 Auto | 7.20 | 15 | Self-Defense |
| Sig Sauer | P320 Compact FDE | Pistol | 9mm | 7.20 | 15 | Self-Defense |
| Sig Sauer | P320 Full-Size | Pistol | 9mm / 40 S&W / 45 Auto | 8.10 | 17 | Self-Defense |
| Sig Sauer | P320 Full-Size RX | Pistol | 9mm | 8.10 | 17 | Self-Defense |
| Sig Sauer | P320 M17 | Pistol | 9mm | 8.10 | 17 | Self-Defense |
| Sig Sauer | P320 MHS | Pistol | 9mm | 8.10 | 17 | Self-Defense |
| Sig Sauer | P320 RX Compact | Pistol | 9mm | 7.20 | 15 | Self-Defense |
| Sig Sauer | P320 Subcompact | Pistol | 9mm / 40 S&W | 6.70 | 12 | Self-Defense |
| Sig Sauer | P320 X Carry | Pistol | 9mm | 7.40 | 17 | Self-Defense |
| Sig Sauer | P320 X Five | Pistol | 9mm | 8.60 | 21 | Self-Defense |
| Sig Sauer | P320 X VTAC | Pistol | 9mm | 8.30 | 17 | Self-Defense |
| Sig Sauer | P365 | Pistol | 9mm | 5.80 | 10 | Self-Defense |
| Sig Sauer | P938 22 Target | Pistol | 22 LR | 5.90 | 10 | Recreation |
| Sig Sauer | P938 Blackwood | Pistol | 9mm | 5.90 | 7 | Self-Defense |
| Sig Sauer | P938 BRG | Pistol | 9mm | 5.90 | 7 | Self-Defense |
| Sig Sauer | P938 Combat | Pistol | 9mm | 5.90 | 7 | Self-Defense |
| Sig Sauer | P938 Emperor Scorpion | Pistol | 9mm | 5.90 | 7 | Self-Defense |
| Sig Sauer | P938 Nightmare | Pistol | 9mm | 5.90 | 7 | Self-Defense |
| Sig Sauer | P938 Nitron | Pistol | 9mm | 5.90 | 7 | Self-Defense |
| Sig Sauer | P938 Rose Gold | Pistol | 9mm | 5.90 | 7 | Self-Defense |
| Sig Sauer | P938 SAS | Pistol | 9mm | 5.90 | 7 | Self-Defense |
| Sig Sauer | P938 Select | Pistol | 9mm | 5.90 | 7 | Self-Defense |
| Sig Sauer | P938 Stand | Pistol | 9mm | 5.90 | 7 | Self-Defense |
| Sig Sauer | P938 We the People | Pistol | 9mm | 5.90 | 7 | Self-Defense |
| Sig Sauer | SIG MPX Pistol | Pistol | 9mm | 22.50 | 30 | Tactical |
| Sig Sauer | SP2022 FDE | Pistol | 9mm | 7.40 | 15 | Self-Defense |
| Sig Sauer | SP2022 Nitron | Pistol | 9mm / 40 S&W | 7.40 | 15 | Self-Defense |
| Smith & Wesson | M&P Bodyguard | Pistol | 380 Auto | 2.75 | 6 | Self Defense |
| Smith & Wesson | M&P M2.0 Carry & Range | Pistol | 40 S&W | 4.25 | 15 | Self Defense |
| Smith & Wesson | M&P M2.0 Carry & Range | Pistol | 9mm | 4.25 | 17 | Self Defense |
| Smith & Wesson | M&P M2.0 Compact | Pistol | 40 S&W | 3.60 | 13 | Self Defense |
| Smith & Wesson | M&P M2.0 Compact | Pistol | 40 S&W | 4 | 13 | Self Defense |
| Smith & Wesson | M&P M2.0 Compact | Pistol | 45 Auto | 4 | 10 | Self Defense |
| Smith & Wesson | M&P M2.0 Compact | Pistol | 9mm | 3.60 | 15 | Self Defense |
| Smith & Wesson | M&P M2.0 Compact | Pistol | 9mm | 4.00 | 15 | Self Defense |
| Smith & Wesson | M&P M2.0 Full | Pistol | 40 S&W | 4.25 | 15 | Self Defense |
| Smith & Wesson | M&P M2.0 Full | Pistol | 40 S&W | 5.00 | 15 | Self Defense |
| Smith & Wesson | M&P M2.0 Full | Pistol | 45 auto | 4.60 | 10 | Self Defense |
| Smith & Wesson | M&P M2.0 Full | Pistol | 9mm | 5.00 | 17 | Self Defense |
| Smith & Wesson | M&P M2.0 Shield | Pistol | 40 S&W | 3.1 | 7 | Self Defense |
| Smith & Wesson | M&P M2.0 Shield | Pistol | 45 Auto | 3.3 | 7 | Self Defense |
| Smith & Wesson | M&P M2.0 Shield | Pistol | 9mm | 3.1 | 8 | Self Defense |
| Smith & Wesson | M&P M2.0 Shield EZ | Pistol | 380 Auto | 3.675 | 8 | Self Defense |
| Smith & Wesson | M&P Original Series-Compact | Pistol | 40 S&W | 3.5 | 10 | Self Defense |
| Smith & Wesson | M&P Original Series-Compact | Pistol | 45 auto | 4 | 8 | Self Defense |
| Smith & Wesson | M&P Original Series-Compact | Pistol | 9mm | 3.5 | 12 | Self Defense |
| Smith & Wesson | M&P Original Series-Full | Pistol | 40 S&W | 4.25 | 15 | Self Defense |
| Smith & Wesson | M&P Original Series-Full | Pistol | 45 auto | 4.5 | 10 | Self Defense |
| Smith & Wesson | M&P Original Series-Full | Pistol | 9mm | 4.25 | 17 | Self Defense |
| Smith & Wesson | M&P Original Series-Shield | Pistol | 40 S&W | 3.1 | 7 | Self Defense |
| Smith & Wesson | M&P Orignial Series-Shield | Pistol | 9mm | 3.1 | 8 | Self Defense |
| Smith & Wesson | M&P22 | Pistol | 22LR | 4.1 | 12 | Self Defense |
| Smith & Wesson | M&P22-compact | Pistol | 22LR | 3.6 | 10 | Self Defense |
| Smith & Wesson | Performance Center Model 41 | Pistol | 22LR | 5.5 | 11 | Recreation |
| Smith & Wesson | Performance Center SW1911 Pro Series | Pistol | 45 auto | 5 | 8 | Recreation |
| Smith & Wesson | Performance Center SW1911 Pro Series | Pistol | 9mm | 5 | 10 | Recreation |
| Smith & Wesson | Performance Center SW22 Victory | Pistol | 22LR | 6 | 11 | Tactical |
| Smith & Wesson | S&W Model 41 | Pistol | 22LR | 7 | 10 | Recreation |
| Smith & Wesson | SD &SDVE | Pistol | 40 S&W | 4 | 14 | Self Defense |
| Smith & Wesson | SD &SDVE | Pistol | 9mm | 4 | 16 | Self Defense |
| Smith & Wesson | SW1911 E-series | Pistol | 45 auto | 5 | 8 | Recreation |
| Smith & Wesson | SW22 Victory Standard | Pistol | 22LR | 5.5 | 10 | Recreation |
| Smith & Wesson | SW22 Victory Target | Pistol | 22LR | 5.5 | 10 | Recreation |
| Springfield | 1911 EMP Models | Pistol | 40 S&W | 7.50 | 9 | Self-Defense |
| Springfield | 1911 EMP Models | Pistol | 9mm | 7.50 | 10 | Self-Defense |
| Springfield | 1911 Loaded Models | Pistol | 45 ACP | 8.60 | 8 | Self-Defense |
| Springfield | 1911 Mil-Spec Models | Pistol | 45 ACP | 8.60 | 7 | Self-Defense |
| Springfield | 1911 RO Elite Models | Pistol | 45 ACP | 8.60 | 7 | Self-Defense |
| Springfield | 1911 RO Elite Models | Pistol | 9mm | 8.60 | 9 | Self-Defense |
| Springfield | 1911 TRP Models | Pistol | 10mm | 9.60 | 8 | Self-Defense |
| Springfield | 1911 TRP Models | Pistol | 45 ACP | 8.60 | 7 | Self-Defense |
| Springfield | 911 Models | Pistol | 380 ACP | 5.50 | 6 | Self-Defense |
| Springfield | SAINT Pistol | Pistol | 5.56 NATO / 300 BLK | 27.75 | 30 | Tactical |
| Springfield | XD Mod. 2 Service Model | Pistol | 40 S&W | 7.30 | 12 | Self-Defense |
| Springfield | XD Mod. 2 Service Model | Pistol | 45 ACP | 7.30 | 13 | Self-Defense |
| Springfield | XD Mod. 2 Service Model | Pistol | 9mm | 7.30 | 16 | Self-Defense |
| Springfield | XD Mod. 2 Sub-Compact Model | Pistol | 40 S&W | 6.25 | 9 | Self-Defense |
| Springfield | XD Mod. 2 Sub-Compact Model | Pistol | 45 ACP | 6.50 | 9 | Self-Defense |
| Springfield | XD Mod. 2 Sub-Compact Model | Pistol | 9mm | 6.25 | 13 | Self-Defense |
| Springfield | XD Mod. 2 Tactical Model | Pistol | 45 ACP | 8.10 | 13 | Self-Defense |
| Springfield | XD Mod. 2 Tactical Model | Pistol | 9mm | 8.30 | 16 | Self-Defense |
| Springfield | XD Service Model | Pistol | 40 S&W | 7.30 | 12 | Self-Defense |
| Springfield | XD Service Model | Pistol | 45 ACP | 7.30 | 13 | Self-Defense |
| Springfield | XD Service Model | Pistol | 9mm | 7.30 | 16 | Self-Defense |
| Springfield | XD Sub-Compact Model | Pistol | 40 S&W | 6.25 | 9 | Self-Defense |
| Springfield | XD Sub-Compact Model | Pistol | 9mm | 6.25 | 13 | Self-Defense |
| Springfield | XD(M) 3.8" Compact Model | Pistol | 40 S&W | 6.75 | 11 | Self-Defense |
| Springfield | XD(M) 3.8" Compact Model | Pistol | 45 ACP | 6.90 | 9 | Self-Defense |
| Springfield | XD(M) 3.8" Compact Model | Pistol | 9mm | 6.75 | 13 | Self-Defense |
| Springfield | XD(M) 3.8" Full Size Model | Pistol | 40 S&W | 6.75 | 16 | Self-Defense |
| Springfield | XD(M) 3.8" Full Size Model | Pistol | 9mm | 6.75 | 19 | Self-Defense |
| Springfield | XD(M) 4.5" Full Size Models | Pistol | 40 S&W | 7.60 | 16 | Self-Defense |
| Springfield | XD(M) 4.5" Full Size Models | Pistol | 45 ACP | 8.40 | 13 | Self-Defense |
| Springfield | XD(M) 4.5" Full Size Models | Pistol | 9mm | 8.25 | 19 | Self-Defense |
| Springfield | XD(M) 5.5" Competition Series | Pistol | 40 S&W | 8.20 | 16 | Recreation |
| Springfield | XD(M) 5.5" Competition Series | Pistol | 45 ACP | 8.30 | 13 | Recreation |
| Springfield | XD(M) 5.5" Competition Series | Pistol | 9mm | 8.20 | 19 | Recreation |
| Springfield | XD-E | Pistol | 45 ACP | 6.75 | 6 | Self-Defense |
| Springfield | XD-E | Pistol | 9mm | 6.75 | 8 | Self-Defense |
| Springfield | XD-S | Pistol | 40 S&W | 6.50 | 6 | Self-Defense |
| Springfield | XD-S | Pistol | 45 ACP | 6.50 | 5 | Self-Defense |
| Springfield | XD-S | Pistol | 9mm | 6.50 | 7 | Self-Defense |
| Steyr | C-A1 | Pistol | 40 S&W | 6.70 | 12 | Self-Defense |
| Steyr | C-A1 | Pistol | 9mm | 6.70 | 17 | Self-Defense |
| Steyr | L-A1 | Pistol | 40 S&W | 7.20 | 12 | Self-Defense |
| Steyr | L-A1 | Pistol | 9mm | 7.20 | 17 | Self-Defense |
| Steyr | M-A1 | Pistol | 40 S&W | 6.90 | 12 | Self-Defense |
| Steyr | M-A1 | Pistol | 9mm | 6.90 | 17 | Self-Defense |
| Steyr | S-A1 | Pistol | 40 S&W | 6.70 | 10 | Self-Defense |
| Steyr | S-A1 | Pistol | 9mm | 6.70 | 10 | Self-Defense |
| Taurus | 92 | Pistol | 9mm | 8.50 | 17 | Self-Defense |
| Taurus | 1911 | Pistol | 45 ACP | 8.50 | 8 | Self-Defense |
| Taurus | 1911 | Pistol | 9mm | 8.50 | 8 | Self-Defense |
| Taurus | 1911 (9-round magazine) | Pistol | 9mm | 8.50 | 9 | Self-Defense |
| Taurus | 1911 Commander | Pistol | 45 ACP | 7.90 | 8 | Self-Defense |
| Taurus | 1911 Officer | Pistol | 45 ACP | 7.20 | 6 | Self-Defense |
| Taurus | 22 Poly | Pistol | 22 LR | 5.00 | 8 | Recreation |
| Taurus | Curve | Pistol | 380 ACP | 5.20 | 6 | Self-Defense |
| Taurus | Millenium G2 | Pistol | 40 S&W | 6.30 | 10 | Self-Defense |
| Taurus | Millenium G2 | Pistol | 9mm | 6.30 | 12 | Self-Defense |
| Taurus | Spectrum | Pistol | 380 ACP | 5.40 | 6 | Self-Defense |
| Glock | G22 | Pistol | 40 S&W | 7.95 | 15 | Self-Defense |
| Glock | G25 | Pistol | 380 Auto | 7.36 | 15 | Self-Defense |
| Glock | G27 | Pistol | 40 S&W | 6.42 | 9 | Self-Defense |
| Glock | G32 | Pistol | 357 Sig | 7.28 | 13 | Self-Defense |
| Glock | G34 | Pistol | 9mm | 8.74 | 17 | Recreation |
| Smith & Wesson | M&P M2.0 Full | Pistol | 9mm | 4.25 | 17 | Self Defense |
| Colt | Bright Cobra | Revolver | .38 Special | 7.2" | 6 | Self-Defense |
| Colt | Classic Cobra | Revolver | .38 Special | 7.2" | 6 | Self-Defense |
| Colt | Cobra TT | Revolver | .38 Special | 7.25" | 6 | Self-Defense |
| Colt | Colt Cobra (38SPL) | Revolver | .38 Special | 7.25" | 6 | Self-Defense |
| Colt | King Cobra | Revolver | .357 Magnum | 7.25" | 6 | Self-Defense |
| Colt | Night Cobra (38SPL) | Revolver | .38 Special | 7.25" | 6 | Self-Defense |
| Heritage | Big Bore Revolver | Revolver | 45LC | 10.2" | 6 | Recreation |
| Heritage | Big Bore Revolver .357 | Revolver | .357 Mag | 9.45" | 9 | Recreation |
| Heritage | Rough Rider Revolver 9 shot Small Bore | Revolver | 22 Magnum | 10" | 9 | Recreation |
| Heritage | Rough Rider Revolver Small Bore | Revolver | 22 Magnum | 10.2" | 6 | Recreation |
| Kimber | K6s CDP | Revolver | 357 Mag | 6.62 | 6 | Self-Defense |
| Kimber | K6s DC | Revolver | 357 Mag | 6.62 | 6 | Self-Defense |
| Kimber | K6s DCR | Revolver | 357 Mag | 6.62 | 6 | Self-Defense |
| Kimber | K6s Stainless | Revolver | 357 Mag | 6.62 | 6 | Self-Defense |
| Kimber | K6s Stainless (3") | Revolver | 357 Mag | 7.62 | 6 | Self-Defense |
| Ruger | Bearcat | Revolver | 22 LR | 9.00 | 6 | Recreation |
| Ruger | Bearcat | Revolver | 22 LR | 9.00 | 6 | Recreation |
| Ruger | Blackhawk | Revolver | 30 Carb | 13.38 | 6 | Recreation |
| Ruger | Blackhawk | Revolver | 357 Mag | 10.50 | 6 | Recreation |
| Ruger | Blackhawk | Revolver | 357 Mag | 12.38 | 6 | Recreation |
| Ruger | Blackhawk | Revolver | 357 Mag | 10.50 | 6 | Recreation |
| Ruger | Blackhawk | Revolver | 357 Mag | 12.38 | 6 | Recreation |
| Ruger | Blackhawk | Revolver | 41 Rem Mag | 10.50 | 6 | Recreation |
| Ruger | Blackhawk | Revolver | 41 Rem Mag | 12.38 | 6 | Recreation |
| Ruger | Blackhawk | Revolver | 45 Colt | 10.50 | 6 | Recreation |
| Ruger | Blackhawk | Revolver | 45 Colt | 13.38 | 6 | Recreation |
| Ruger | Blackhawk | Revolver | 45 Colt | 11.38 | 6 | Recreation |
| Ruger | Blackhawk Convertible | Revolver | 357 Mag / 9mm | 10.50 | 6 | Recreation |
| Ruger | Blackhawk Convertible | Revolver | 357 Mag / 9mm | 12.38 | 6 | Recreation |
| Ruger | Blackhawk Convertible | Revolver | 45 Colt / 45 Auto | 10.50 | 6 | Recreation |
| Ruger | Blackhawk Convertible | Revolver | 45 Colt / 45 Auto | 11.38 | 6 | Recreation |
| Ruger | GP100 | Revolver | 22 LR | 11.30 | 10 | Recreation |
| Ruger | GP100 | Revolver | 357 Mag | 8.50 | 6 | Recreation |
| Ruger | GP100 | Revolver | 357 Mag | 9.50 | 6 | Recreation |
| Ruger | GP100 | Revolver | 357 Mag | 9.50 | 6 | Recreation |
| Ruger | GP100 | Revolver | 357 Mag | 11.50 | 6 | Recreation |
| Ruger | GP100 | Revolver | 357 Mag | 11.50 | 6 | Recreation |
| Ruger | GP100 | Revolver | 44 Special | 8.50 | 5 | Recreation |
| Ruger | GP100 7-Round | Revolver | 327 Fed Mag | 9.50 | 7 | Recreation |
| Ruger | GP100 7-Round | Revolver | 327 Fed Mag | 11.50 | 7 | Recreation |
| Ruger | GP100 7-Round | Revolver | 357 Mag | 8.00 | 7 | Recreation |
| Ruger | GP100 7-Round | Revolver | 357 Mag | 9.50 | 7 | Recreation |
| Ruger | GP100 7-Round | Revolver | 357 Mag | 10.38 | 7 | Recreation |
| Ruger | GP100 Match Champion | Revolver | 357 Mag | 9.50 | 6 | Recreation |
| Ruger | GP100 Match Champion | Revolver | 357 Mag | 9.50 | 6 | Recreation |
| Ruger | LCR | Revolver | 22 LR | 6.50 | 8 | Recreation |
| Ruger | LCR | Revolver | 22 WMR | 6.50 | 6 | Recreation |
| Ruger | LCR | Revolver | 327 Fed Mag | 6.50 | 6 | Recreation |
| Ruger | LCR | Revolver | 357 Mag | 6.50 | 5 | Recreation |
| Ruger | LCR | Revolver | 38 Spl +P | 6.50 | 5 | Recreation |
| Ruger | LCR | Revolver | 9mm | 6.50 | 5 | Recreation |
| Ruger | LCRx | Revolver | 22 LR | 7.50 | 8 | Recreation |
| Ruger | LCRx | Revolver | 22 WMR | 6.50 | 6 | Recreation |
| Ruger | LCRx | Revolver | 22 WMR | 7.50 | 6 | Recreation |
| Ruger | LCRx | Revolver | 327 Fed Mag | 6.50 | 6 | Recreation |
| Ruger | LCRx | Revolver | 357 Mag | 6.50 | 5 | Recreation |
| Ruger | LCRx | Revolver | 38 Spl +P | 6.50 | 5 | Recreation |
| Ruger | LCRx | Revolver | 38 Spl +P | 7.50 | 5 | Recreation |
| Ruger | LCRx | Revolver | 9mm | 6.50 | 5 | Recreation |
| Ruger | Redhawk | Revolver | 357 Mag | 9.50 | 8 | Recreation |
| Ruger | Redhawk | Revolver | 357 Mag | 11.00 | 8 | Recreation |
| Ruger | Redhawk | Revolver | 357 Mag | 8.25 | 8 | Recreation |
| Ruger | Redhawk | Revolver | 44 Rem Mag | 11.00 | 6 | Recreation |
| Ruger | Redhawk | Revolver | 44 Rem Mag | 13.00 | 6 | Recreation |
| Ruger | Redhawk | Revolver | 44 Rem Mag | 9.50 | 6 | Recreation |
| Ruger | Redhawk | Revolver | 45 Auto / 45 Colt | 9.50 | 6 | Recreation |
| Ruger | Redhawk Hunter | Revolver | 44 Rem Mag | 13.00 | 6 | Recreation |
| Ruger | Single-Nine | Revolver | 22 WMR | 12.00 | 9 | Recreation |
| Ruger | Single-Six | Revolver | 17 HMR | 12.00 | 6 | Recreation |
| Ruger | Single-Six Convertible | Revolver | 22 LR / 22 WMR | 11.00 | 6 | Recreation |
| Ruger | Single-Six Convertible | Revolver | 22 LR / 22 WMR | 12.00 | 6 | Recreation |
| Ruger | Single-Six Convertible | Revolver | 22 LR / 22 WMR | 10.25 | 6 | Recreation |
| Ruger | Single-Six Convertible | Revolver | 22 LR / 22 WMR | 11.00 | 6 | Recreation |
| Ruger | Single-Six Convertible | Revolver | 22 LR / 22 WMR | 12.00 | 6 | Recreation |
| Ruger | Single-Six Convertible | Revolver | 22 LR / 22 WMR | 15.00 | 6 | Recreation |
| Ruger | Single-Six Convertible | Revolver | 22 LR / 22 WMR | 11.00 | 6 | Recreation |
| Ruger | Single-Six Hunter | Revolver | 22 LR / 22 WMR | 13.00 | 6 | Recreation |
| Ruger | Single-Ten | Revolver | 22 LR | 11.00 | 10 | Recreation |
| Ruger | SP101 | Revolver | 22 LR | 9.12 | 8 | Recreation |
| Ruger | SP101 | Revolver | 327 Fed Mag | 9.12 | 6 | Recreation |
| Ruger | SP101 | Revolver | 327 Fed Mag | 8.00 | 6 | Recreation |
| Ruger | SP101 | Revolver | 357 Mag | 7.20 | 5 | Recreation |
| Ruger | SP101 | Revolver | 357 Mag | 8.00 | 5 | Recreation |
| Ruger | SP101 | Revolver | 357 Mag | 7.20 | 5 | Recreation |
| Ruger | SP101 | Revolver | 357 Mag | 9.12 | 5 | Recreation |
| Ruger | SP101 | Revolver | 38 Spl +P | 7.20 | 5 | Recreation |
| Ruger | SP101 | Revolver | 9mm | 7.20 | 5 | Recreation |
| Ruger | SP101 Match Champion | Revolver | 357 Mag | 9.12 | 5 | Recreation |
| Ruger | Super Blackhawk | Revolver | 44 Rem Mag | 10.50 | 6 | Recreation |
| Ruger | Super Blackhawk | Revolver | 44 Rem Mag | 10.50 | 6 | Recreation |
| Ruger | Super Blackhawk | Revolver | 44 Rem Mag | 11.38 | 6 | Recreation |
| Ruger | Super Blackhawk | Revolver | 44 Rem Mag | 11.38 | 6 | Recreation |
| Ruger | Super Blackhawk | Revolver | 44 Rem Mag | 13.38 | 6 | Recreation |
| Ruger | Super Blackhawk | Revolver | 44 Rem Mag | 16.38 | 6 | Recreation |
| Ruger | Super Blackhawk | Revolver | 44 Rem Mag | 13.38 | 6 | Recreation |
| Ruger | Super Blackhawk | Revolver | 44 Rem Mag | 16.38 | 6 | Recreation |
| Ruger | Super Blackhawk Bisley Hunter | Revolver | 44 Rem Mag | 13.12 | 6 | Recreation |
| Ruger | Super Blackhawk Hunter | Revolver | 44 Rem Mag | 13.63 | 6 | Recreation |
| Ruger | Super Redhawk | Revolver | 10mm Auto | 12.00 | 6 | Recreation |
| Ruger | Super Redhawk | Revolver | 44 Rem Mag | 13.00 | 6 | Recreation |
| Ruger | Super Redhawk | Revolver | 44 Rem Mag | 15.00 | 6 | Recreation |
| Ruger | Super Redhawk | Revolver | 454 Casull | 13.00 | 6 | Recreation |
| Ruger | Super Redhawk | Revolver | 480 Ruger | 13.00 | 6 | Recreation |
| Ruger | Super Redhawk Alaskan | Revolver | 44 Rem Mag | 7.62 | 6 | Recreation |
| Ruger | Super Redhawk Alaskan | Revolver | 454 Casull | 7.62 | 6 | Recreation |
| Ruger | Super Redhawk Alaskan | Revolver | 480 Ruger | 7.62 | 6 | Recreation |
| Ruger | Vaquero | Revolver | 357 Mag | 10.25 | 6 | Recreation |
| Ruger | Vaquero | Revolver | 357 Mag | 11.12 | 6 | Recreation |
| Ruger | Vaquero | Revolver | 357 Mag | 10.25 | 6 | Recreation |
| Ruger | Vaquero | Revolver | 357 Mag | 11.12 | 6 | Recreation |
| Ruger | Vaquero | Revolver | 357 Mag | 11.12 | 6 | Recreation |
| Ruger | Vaquero | Revolver | 357 Mag | 10.25 | 6 | Recreation |
| Ruger | Vaquero | Revolver | 45 Colt | 10.25 | 6 | Recreation |
| Ruger | Vaquero | Revolver | 45 Colt | 11.12 | 6 | Recreation |
| Ruger | Vaquero | Revolver | 45 Colt | 10.25 | 6 | Recreation |
| Ruger | Vaquero | Revolver | 45 Colt | 11.12 | 6 | Recreation |
| Ruger | Vaquero | Revolver | 45 Colt | 11.12 | 6 | Recreation |
| Ruger | Vaquero | Revolver | 45 Colt | 11.12 | 6 | Recreation |
| Smith & Wesson | M&P Bodyguard | Revolver | 38 S&W Special +P | 1.9 | 5 | Self Defense |
| Smith & Wesson | Performance Center - Hunting Revolver | Revolver | 44 Magnum/44 Special | 7.5 | 6 | Recreation |
| Smith & Wesson | Performance Center - Hunting Revolver | Revolver | 460 S&W Magnum | 14 | 5 | Recreation |
| Smith & Wesson | Performance Center - Hunting Revolver | Revolver | 500 S&W Magnum | 10.5 | 5 | Recreation |
| Smith & Wesson | Performance Center - Large Frame Revolver | Revolver | 357 Magnum/38 S&W Special +P | 5 | 8 | Recreation |
| Smith & Wesson | Performance Center - Large Frame Revolver | Revolver | 44 Magnum/44 special | 8.4 | 6 | Recreation |
| Smith & Wesson | Performance Center - Large Frame Revolver | Revolver | 45 ACP | 4 | 6 | Recreation |
| Smith & Wesson | Performance Center - Large Frame Revolver | Revolver | 9mm | 6.5 | 8 | Recreation |
| Smith & Wesson | Performance Center - Medium Frame Revolver | Revolver | 357 Magnum/38 S&W Special +P | 5 | 7 | Recreation |
| Smith & Wesson | Performance Center - Medium Frame Revolver | Revolver | 9mm | 2.5 | 7 | Recreation |
| Smith & Wesson | Performance Center Large Frame Revolver Pro Series | Revolver | 357 Magnum/38 S&W Special +P | 4 | 8 | Recreation |
| Smith & Wesson | Performance Center Medium Frame Revolver Pro Series | Revolver | 358 Magnum/38 S&W Special +P | 5 | 7 | Recreation |
| Smith & Wesson | Performance Center Medium Frame Revolver Pro Series | Revolver | 9mm | 5 | 7 | Recreation |
| Smith & Wesson | Performance Center Small Frame Revolver | Revolver | 38 S&W Special +P | 1.88 | 5 | Recreation |
| Smith & Wesson | Performance Center Small Frame Revolver Pro Series | Revolver | 357 Magnum/38 S&W Special +P | 3 | 5 | Recreation |
| Smith & Wesson | Performance Center Small Frame Revolver Pro Series | Revolver | 38 S&W Special +P | 1.88 | 5 | Recreation |
| Smith & Wesson | S&W Classics - Model 10 | Revolver | 38 S&W Special +P | 4 | 6 | Recreation |
| Smith & Wesson | S&W Classics - Model 17 | Revolver | 22LR | 6 | 6 | Recreation |
| Smith & Wesson | S&W Classics - Model 25 | Revolver | 45 Colt | 6.5 | 6 | Recreation |
| Smith & Wesson | S&W Classics - Model 27 | Revolver | 357 Magnum/38 S&W Specail +P | 6.5 | 6 | Recreation |
| Smith & Wesson | S&W Classics - Model 36 | Revolver | 38 S&W Special +P | 1.88 | 5 | Recreation |
| Smith & Wesson | S&W Classics - Model 48 | Revolver | 22 Magnum | 6 | 6 | Recreation |
| Smith & Wesson | S&W Classics -Model 29 | Revolver | 44 Magnum/44 Special | 6.5 | 6 | Recreation |
| Smith & Wesson | S&W Classics- Model 57 | Revolver | 41 Magnum | 6 | 6 | Recreation |
| Smith & Wesson | SW Governor | Revolver | 410 Shotshell 2-1/2'/45 ACP/45 Colt | 2.75 | 6 | Self Defense |
| Smith & Wesson | SW K-frame Revolver | Revolver | 22LR | 6 | 10 | Self Defense |
| Smith & Wesson | SW K-frame Revolver | Revolver | 357 Magnum/38 S&W Special +P | 4.25 | 6 | Self Defense |
| Smith & Wesson | SW K-frame Revolver | Revolver | 38 S&W Special +P | 4.13 | 6 | Self Defense |
| Smith & Wesson | SW Large Frame Revolver | Revolver | 44 Magnum | 6.5 | 6 | Recreation |
| Smith & Wesson | SW Large Frame Revolver | Revolver | 45 ACP | 4.13 | 6 | Recreation |
| Smith & Wesson | SW L-frame Revolver | Revolver | 357 Magnum/38 S&W Special +P | 7 | 7 | Self Defense |
| Smith & Wesson | SW L-frame Revolver | Revolver | 44 Magnum | 4.25 | 5 | Self Defense |
| Smith & Wesson | SW Small Frame Revolver | Revolver | 22 Magnum | 1.88 | 7 | Self Defense |
| Smith & Wesson | SW Small Frame Revolver | Revolver | 22LR | 3 | 8 | Self Defense |
| Smith & Wesson | SW Small Frame Revolver | Revolver | 357 Magnum/38 S&W Special +P | 3 | 5 | Self Defense |
| Smith & Wesson | SW Small Frame Revolver | Revolver | 38 S&W Special +P | 1.88 | 5 | Self Defense |
| Smith & Wesson | SW X-Large Frame Revolver | Revolver | 460 S&W Magnum | 8.38 | 5 | Recreation |
| Smith & Wesson | SW X-Large Frame Revolver | Revolver | 500 S&W Magnum | 8.38 | 5 | Recreation |
| Taurus | 44 | Revolver | 44 Mag | 13.75 | 6 | Recreation |
| Taurus | 65 | Revolver | 357 Mag / 38 Spl | 10.50 | 6 | Self-Defense |
| Taurus | 66 | Revolver | 357 Mag / 38 Spl | 12.25 | 7 | Self-Defense |
| Taurus | 82 | Revolver | 38 Spl | 9.25 | 6 | Self-Defense |
| Taurus | 380 | Revolver | 380 Auto | 5.95 | 5 | Self-Defense |
| Taurus | 605 | Revolver | 357 Mag / 38 Spl | 6.50 | 5 | Self-Defense |
| Taurus | 608 | Revolver | 357 Mag / 38 Spl | 11.68 | 8 | Self-Defense |
| Taurus | 617 | Revolver | 357 Mag / 38 Spl | 6.63 | 7 | Self-Defense |
| Taurus | 692 | Revolver | 9mm / 357 Mag / 38 Spl | 11.64 | 7 | Self-Defense |
| Taurus | 856 | Revolver | 38 Spl | 6.55 | 6 | Self-Defense |
| Taurus | 905 | Revolver | 9mm | 6.50 | 5 | Self-Defense |
| Taurus | 605 Protector | Revolver | 357 Mag / 38 Spl | 6.70 | 5 | Self-Defense |
| Taurus | Judge | Revolver | 45 Colt | 12.50 | 5 | Self-Defense |
| Taurus | Judge Magnum | Revolver | 45 Colt | 12.50 | 5 | Self-Defense |
| Taurus | Judge Public Defender | Revolver | 45 Colt | 7.65 | 5 | Self-Defense |
| Taurus | Raging Bull 444 | Revolver | 44 Mag | 14.00 | 6 | Recreation |
| Taurus | Raging Bull 444 Multi | Revolver | 44 Mag | 9.80 | 6 | Recreation |
| Taurus | Raging Bull 454 | Revolver | 454 Casull | 14.00 | 5 | Recreation |
| Taurus | Raging Hunter | Revolver | 44 Mag | 15.75 | 6 | Recreation |
| Taurus | Raging Judge 513 | Revolver | 45 Colt | 14.10 | 6 | Self-Defense |
| Taurus | Tracker 17 | Revolver | 17 HMR | 10.75 | 7 | Recreation |
| Taurus | Tracker 44 | Revolver | 44 Mag | 9.00 | 5 | Recreation |
| Taurus | Tracker 627 | Revolver | 357 Mag / 38 Spl | 10.75 | 7 | Recreation |
| Taurus | Tracker 992 | Revolver | 22 LR | 11.20 | 9 | Recreation |
| Alexander | .17 HMR Series (Complete and Tactical) | Rifle | 17 HMR | 37.00 | 10 | Tactical |
| Alexander | .50 Beowulf Entry Series | Rifle | 50 Beowulf | 36.00 | 7 | Tactical |
| Alexander | .50 Beowulf Hunter Series | Rifle | 50 Beowulf | 38.00 | 7 | Recreation |
| Alexander | .50 Beowulf Tactical Series | Rifle | 50 Beowulf | 36.00 | 7 | Tactical |
| Alexander | 300 AAC Series (Complete and Tactical) | Rifle | 300 AAC | 37.00 | 30 | Tactical |
| Alexander | 5.56 NATO Incursion Series | Rifle | 5.56 NATO | 26.00 | 30 | Tactical |
| Alexander | 5.56 NATO Tactical Series | Rifle | 5.56 NATO | 36.00 | 30 | Tactical |
| Alexander | 6.5 Grendel Advanced Weapon System | Rifle | 6.5 Grendel | 38.00 | 10 | Tactical |
| Alexander | 6.5 Grendel Entry Series | Rifle | 6.5 Grendel | 38.00 | 10 | Tactical |
| Alexander | 6.5 Grendel Hunter Series | Rifle | 6.5 Grendel | 37.00 | 10 | Recreation |
| Alexander | 6.5 Grendel Incursion Series | Rifle | 6.5 Grendel | 37.00 | 10 | Tactical |
| Alexander | 6.5 Grendel Lite Series | Rifle | 6.5 Grendel | 37.00 | 10 | Tactical |
| Alexander | 6.5 Grendel Overwatch Series | Rifle | 6.5 Grendel | 42.50 | 10 | Tactical |
| Alexander | 6.5 Grendel Tactical Series | Rifle | 6.5 Grendel | 36.00 | 10 | Tactical |
| Alexander | Ulfberht .338 Lapua Magnum Rifle | Rifle | 338 Lapua Magnum | 44.00 | 10 | Tactical |
| Anderson | AM-10 EXT Hunter | Rifle | .308 Win | 20" | 20 | Tactical |
| Anderson | AM-10 EXT Hunter | Rifle | .308 Win | 16" | 20 | Tactical |
| Anderson | AM-10 EXT Hunter | Rifle | .308 Win | 18" | 20 | Tactical |
| Anderson | AM-10 EXT Sniper | Rifle | .308 Win | 24" | 20 | Tactical |
| Anderson | AM-15 EXT LE Front Sight Base | Rifle | 5.56 NATO | 36 1/8" | 30 | Tactical |
| Anderson | AM-15 EXT Sniper | Rifle | 5.56 NATO | 24" | 30 | Tactical |
| Anderson | AM-15 EXT Tac | Rifle | 5.56 NATO | 36 1/8" | 30 | Tactical |
| Anderson | AM-15 Keymod | Rifle | .300 AAC Blackout | 36 1/8" | 30 | Tactical |
| Anderson | AM-15 Keymod | Rifle | 5.56 NATO | 37 1/8" | 30 | Tactical |
| Anderson | AM-15 Optic Ready | Rifle | 5.56 NATO | 36 1/8" | 10 | Tactical |
| Anderson | AM-15 Optic Ready | Rifle | 6.5 Grendel | 36 1/8" | 30 | Tactical |
| Anderson | AM-9 Complete Rifle Assy | Rifle | 9MM | 36 1/8" | 17 | Tactical |
| Anderson | Complete Rifle Assy 243 | Rifle | .243 Win | 20" | 20 | Tactical |
| Anderson | Complete Rifle Assy AM-9 | Rifle | 9MM | 40" | 17 | Tactical |
| Anderson | Complete Rifle Assy Creedmoor | Rifle | 6.5 Creedmoor | 20" | 20 | Tactical |
| Anderson | Complete Rifle Assy, AM-15 BR | Rifle | 5.56 NATO | 36 1/8" | 30 | Tactical |
| Anderson | Complete Rifle Assy, AM-15 Bushmaster | Rifle | .45 Bushmaster | 30" | 5 | Tactical |
| Anderson | Complete Rifle Assy, AM-9 M-LOK | Rifle | 9MM | 40" | 17 | Tactical |
| Armalite | 3GN 13 (AR10) | Rifle | 7.62x51mm / 308 Win | 41.25 | 25 | Tactical |
| Armalite | 3GN 13 (M15) | Rifle | 5.56x45mm / .223 | 40.50 | 30 | Tactical |
| Armalite | 3GN 18 (AR10) | Rifle | 7.62x51mm / 308 Win | 41.25 | 25 | Tactical |
| Armalite | 3GN 18 (M15) | Rifle | 5.56x45mm / .223 | 40.50 | 30 | Tactical |
| Armalite | AR30 | Rifle | 300 Win | 48.10 | 5 | Recreation |
| Armalite | AR30A1 | Rifle | 300 Win | 48.10 | 5 | Recreation |
| Armalite | AR31 | Rifle | 7.62x51mm / 308 Win | 47.40 | 10 | Recreation |
| Armalite | AR50 | Rifle | 50 BMG | 58.50 | 1 | Recreation |
| Armalite | DEF 10 (AR10) | Rifle | 7.62x51mm / 308 Win | 38.30 | 20 | Tactical |
| Armalite | DEF 15 (M15) | Rifle | 5.56x45mm / .223 | 35.30 | 30 | Tactical |
| Armalite | DEF 15F (M15) | Rifle | 5.56x45mm / .223 | 35.30 | 30 | Tactical |
| Armalite | LTC 16 (M15) | Rifle | 5.56x45mm / .223 | 35.25 | 30 | Tactical |
| Armalite | M15 T (M15) | Rifle | .223 WYLDE | 38.40 | 10 | Recreation |
| Armalite | M15 TBN (M15) | Rifle | .223 WYLDE | 38.50 | 10 | Recreation |
| Armalite | SASS MKII (AR10) | Rifle | 260 REM / 308 Win / 7.62x51mm | 41.30 | 20 | Tactical |
| Armalite | SuperSASS (AR10) | Rifle | 7.62x51mm / 308 Win | 42.10 | 20 | Tactical |
| Armalite | TAC 14 (AR10) | Rifle | 7.62x51mm / 308 Win | 37.30 | 25 | Tactical |
| Armalite | TAC 14 (M15) | Rifle | 5.56x45mm / .223 | 35.30 | 30 | Tactical |
| Armalite | TAC 16 (AR10) | Rifle | 7.62x51mm / 308 Win | 40.00 | 25 | Tactical |
| Armalite | TAC 16 (M15) | Rifle | 5.56x45mm / .223 | 36.60 | 30 | Tactical |
| Armalite | TAC 18 (AR10) | Rifle | 7.62x51mm / 308 Win | 43.25 | 25 | Tactical |
| Armalite | TAC 18 (M15) | Rifle | 5.56x45mm / .223 | 39.90 | 30 | Tactical |
| Armalite | TAC 20 (AR10) | Rifle | 7.62x51mm / 308 Win | 43.25 | 25 | Tactical |
| Armalite | TBNF (AR10) | Rifle | 7.62x51mm / 308 Win | 39.60 | 10 | Tactical |
| Barrett | 82A1 | Rifle | 50 BMG / 416 Barrett | 57.00 | 10 | Tactical |
| Barrett | M107A1 | Rifle | 50 BMG | 57.00 | 10 | Tactical |
| Barrett | Model 95 | Rifle | 50 BMG | 45.00 | 5 | Recreation |
| Barrett | Model 99 | Rifle | 50 BMG / 416 Barrett | 50.00 | 1 | Recreation |
| Barrett | MRAD | Rifle | Multiple | 49.40 | 10 | Tactical |
| Barrett | REC7 Carbine | Rifle | 5.56 NATO / 6.8 SPC | 33.25 | 30 | Tactical |
| Barrett | REC7 DI Carbine | Rifle | 5.56 NATO / 300 BLK / 6.8 SPC | 36.00 | 30 | Tactical |
| Barrett | REC7 DI DMR | Rifle | 5.56 NATO | 38.00 | 20 | Tactical |
| Barrett | REC7 DI SBR | Rifle | 5.56 NATO / 300 BLK | 30.25 | 30 | Tactical |
| Barrett | REC7 DMR | Rifle | 5.56 NATO | 38.30 | 20 | Tactical |
| Barrett | REC7 Flyweight | Rifle | 5.56 NATO | 34.50 | 20 | Tactical |
| Barrett | REC7 SBR | Rifle | 5.56 NATO | 25.90 | 30 | Tactical |
| Beretta | ARX 100 | Rifle | 223 Rem | 26.50 | 29 | Tactical |
| Beretta | ARX 160 | Rifle | 22 LR | 24.57 | 5 | Tactical |
| Beretta | ARX 160 with pistol stock | Rifle | 22 LR | 18.90 | 5 | Tactical |
| Browning | AB3 Composite Stalker | Rifle | 243 Win | 22 | 6 | Recreation |
| Browning | AB3 Composite Stalker | Rifle | 270 Win | 22 | 5 | Recreation |
| Browning | AB3 Composite Stalker | Rifle | 270 WSM | 23 | 4 | Recreation |
| Browning | AB3 Composite Stalker | Rifle | 300 Win Mag | 26 | 4 | Recreation |
| Browning | AB3 Composite Stalker | Rifle | 300 WSM | 23 | 4 | Recreation |
| Browning | AB3 Composite Stalker | Rifle | 30-06 Sprg | 22 | 5 | Recreation |
| Browning | AB3 Composite Stalker | Rifle | 308 Win | 22 | 6 | Recreation |
| Browning | AB3 Composite Stalker | Rifle | 6.5 Creedmoor | 22 | 6 | Recreation |
| Browning | AB3 Composite Stalker | Rifle | 7mm Rem Mag | 26 | 4 | Recreation |
| Browning | AB3 Composite Stalker | Rifle | 7mm-08 Rem | 22 | 6 | Recreation |
| Browning | AB3 Hunter | Rifle | 243 Win | 22 | 6 | Recreation |
| Browning | AB3 Hunter | Rifle | 270 Win | 22 | 5 | Recreation |
| Browning | AB3 Hunter | Rifle | 270 WSM | 22 | 4 | Recreation |
| Browning | AB3 Hunter | Rifle | 300 Win Mag | 26 | 4 | Recreation |
| Browning | AB3 Hunter | Rifle | 300 WSM | 22 | 4 | Recreation |
| Browning | AB3 Hunter | Rifle | 30-06 Sprg | 22 | 5 | Recreation |
| Browning | AB3 Hunter | Rifle | 308 Win | 22 | 6 | Recreation |
| Browning | AB3 Hunter | Rifle | 6.5 Creedmor | 22 | 6 | Recreation |
| Browning | AB3 Hunter | Rifle | 7mm Rem Mag | 26 | 4 | Recreation |
| Browning | AB3 Hunter | Rifle | 7mm-08 Rem | 22 | 6 | Recreation |
| Browning | AB3 Micro Stalker | Rifle | 243 Win | 20 | 6 | Recreation |
| Browning | AB3 Micro Stalker | Rifle | 308 Win | 20 | 6 | Recreation |
| Browning | AB3 Micro Stalker | Rifle | 6.5 Creedmoor | 20 | 6 | Recreation |
| Browning | AB3 Micro Stalker | Rifle | 7mm-08 Rem | 20 | 6 | Recreation |
| Browning | BAR Mark II Safari | Rifle | 25-06 Rem | 24 | 5 | Recreation |
| Browning | BAR Mark II Safari | Rifle | 270 Win | 22 | 5 | Recreation |
| Browning | BAR Mark II Safari | Rifle | 300 Win Mag | 24 | 4 | Recreation |
| Browning | BAR Mark II Safari | Rifle | 30-06 Sprg | 22 | 5 | Recreation |
| Browning | BAR Mark II Safari | Rifle | 308 Win | 22 | 5 | Recreation |
| Browning | BAR Mark II Safari w boss | Rifle | 270 Win | 22 | 5 | Recreation |
| Browning | BAR Mark II Safari w boss | Rifle | 300 Win Mag | 24 | 4 | Recreation |
| Browning | BAR Mark II Safari w boss | Rifle | 30-06 Sprg | 22 | 5 | Recreation |
| Browning | BAR Mark II Safari w boss | Rifle | 338 Win Mag | 24 | 4 | Recreation |
| Browning | BAR MK3 | Rifle | 243 Win | 22 | 5 | Recreation |
| Browning | BAR MK3 | Rifle | 270 Win | 22 | 5 | Recreation |
| Browning | BAR MK3 | Rifle | 270 WSM | 23 | 4 | Recreation |
| Browning | BAR MK3 | Rifle | 300 Win Mag | 24 | 4 | Recreation |
| Browning | BAR MK3 | Rifle | 300 WSM | 23 | 4 | Recreation |
| Browning | BAR MK3 | Rifle | 30-06 Sprg | 22 | 5 | Recreation |
| Browning | BAR MK3 | Rifle | 308 Win | 22 | 5 | Recreation |
| Browning | BAR MK3 | Rifle | 7mm Rem Mag | 24 | 4 | Recreation |
| Browning | BAR MK3 | Rifle | 7mm-08 Rem | 22 | 5 | Recreation |
| Browning | BAR MK3 DBM | Rifle | 308 Win | 18 | 11 | Recreation |
| Browning | BAR MK3 Stalker | Rifle | 243 Win | 22 | 5 | Recreation |
| Browning | BAR MK3 Stalker | Rifle | 270 Win | 22 | 5 | Recreation |
| Browning | BAR MK3 Stalker | Rifle | 270 WSM | 23 | 4 | Recreation |
| Browning | BAR MK3 Stalker | Rifle | 300 Win Mag | 24 | 4 | Recreation |
| Browning | BAR MK3 Stalker | Rifle | 300 WSM | 23 | 4 | Recreation |
| Browning | BAR MK3 Stalker | Rifle | 30-06 Sprg | 22 | 5 | Recreation |
| Browning | BAR MK3 Stalker | Rifle | 308 Win | 22 | 5 | Recreation |
| Browning | BAR MK3 Stalker | Rifle | 7mm Rem Mag | 24 | 4 | Recreation |
| Browning | BAR MK3 Stalker | Rifle | 7mm-08 Rem | 22 | 5 | Recreation |
| Browning | BAR MK3, MOBUC | Rifle | 243 Win | 22 | 5 | Recreation |
| Browning | BAR MK3, MOBUC | Rifle | 270 Win | 22 | 5 | Recreation |
| Browning | BAR MK3, MOBUC | Rifle | 270 WSM | 23 | 4 | Recreation |
| Browning | BAR MK3, MOBUC | Rifle | 300 Win Mag | 24 | 4 | Recreation |
| Browning | BAR MK3, MOBUC | Rifle | 300 WSM | 23 | 4 | Recreation |
| Browning | BAR MK3, MOBUC | Rifle | 30-06 Sprg | 22 | 5 | Recreation |
| Browning | BAR MK3, MOBUC | Rifle | 308 Win | 22 | 5 | Recreation |
| Browning | BAR MK3, MOBUC | Rifle | 7mm Rem Mag | 24 | 4 | Recreation |
| Browning | BAR MK3, MOBUC | Rifle | 7mm-08 Rem | 22 | 5 | Recreation |
| Browning | BL-22 Grade I | Rifle | 22 S,L & LR | 20 | 16 | Recreation |
| Browning | BL-22 Grade I Micro Midas | Rifle | 22 S,L & LR | 16-1/4 | 12 | Recreation |
| Browning | BL-22 Grade I, FLD | Rifle | 22 S,L & LR | 20 | 16 | Recreation |
| Browning | BL-22 Grade II | Rifle | 22 S,L & LR | 20 | 16 | Recreation |
| Browning | BL-22 Grade II Oct, FLD | Rifle | 22 S,L & LR | 24 | 16 | Recreation |
| Browning | BL-22 Grade II, FLD | Rifle | 22 S,L & LR | 20 | 16 | Recreation |
| Browning | BLR Lightweight '81 | Rifle | 22-250 Rem | 20 | 5 | Recreation |
| Browning | BLR Lightweight '81 | Rifle | 223 Rem | 20 | 5 | Recreation |
| Browning | BLR Lightweight '81 | Rifle | 243 Win | 20 | 5 | Recreation |
| Browning | BLR Lightweight '81 | Rifle | 270 Win | 22 | 5 | Recreation |
| Browning | BLR Lightweight '81 | Rifle | 30-06 Sprg | 22 | 5 | Recreation |
| Browning | BLR Lightweight '81 | Rifle | 308 Win | 20 | 5 | Recreation |
| Browning | BLR Lightweight '81 | Rifle | 358 Win | 20 | 5 | Recreation |
| Browning | BLR Lightweight '81 | Rifle | 7mm-08 Rem | 20 | 5 | Recreation |
| Browning | BLR Lightweight '81 Stainless takedown | Rifle | 22-250 Rem | 20 | 5 | Recreation |
| Browning | BLR Lightweight '81 Stainless takedown | Rifle | 223 Rem | 20 | 5 | Recreation |
| Browning | BLR Lightweight '81 Stainless takedown | Rifle | 243 Win | 20 | 5 | Recreation |
| Browning | BLR Lightweight '81 Stainless takedown | Rifle | 270 Win | 22 | 5 | Recreation |
| Browning | BLR Lightweight '81 Stainless takedown | Rifle | 300 Win Mag | 24 | 4 | Recreation |
| Browning | BLR Lightweight '81 Stainless takedown | Rifle | 300 WSM | 22 | 4 | Recreation |
| Browning | BLR Lightweight '81 Stainless takedown | Rifle | 30-06 Sprg | 22 | 5 | Recreation |
| Browning | BLR Lightweight '81 Stainless takedown | Rifle | 308 Win | 20 | 5 | Recreation |
| Browning | BLR Lightweight '81 Stainless takedown | Rifle | 358 Win | 20 | 5 | Recreation |
| Browning | BLR Lightweight '81 Stainless takedown | Rifle | 450 Marlin | 20 | 4 | Recreation |
| Browning | BLR Lightweight '81 Stainless takedown | Rifle | 7mm Rem Mag | 24 | 4 | Recreation |
| Browning | BLR Lightweight '81 Stainless takedown | Rifle | 7mm-08 Rem | 20 | 5 | Recreation |
| Browning | BLR Lightweight Stainless w pistol grip | Rifle | 22-250 Rem | 20 | 5 | Recreation |
| Browning | BLR Lightweight Stainless w pistol grip | Rifle | 223 Rem | 20 | 5 | Recreation |
| Browning | BLR Lightweight Stainless w pistol grip | Rifle | 243 Win | 20 | 5 | Recreation |
| Browning | BLR Lightweight Stainless w pistol grip | Rifle | 270 Win | 22 | 5 | Recreation |
| Browning | BLR Lightweight Stainless w pistol grip | Rifle | 270 WSM | 22 | 4 | Recreation |
| Browning | BLR Lightweight Stainless w pistol grip | Rifle | 300 Win Mag | 24 | 4 | Recreation |
| Browning | BLR Lightweight Stainless w pistol grip | Rifle | 300 WSM | 22 | 4 | Recreation |
| Browning | BLR Lightweight Stainless w pistol grip | Rifle | 30-06 Sprg | 22 | 5 | Recreation |
| Browning | BLR Lightweight Stainless w pistol grip | Rifle | 308 Win | 20 | 5 | Recreation |
| Browning | BLR Lightweight Stainless w pistol grip | Rifle | 358 Win | 20 | 5 | Recreation |
| Browning | BLR Lightweight Stainless w pistol grip | Rifle | 450 Marlin | 20 | 4 | Recreation |
| Browning | BLR Lightweight Stainless w pistol grip | Rifle | 7mm Rem Mag | 24 | 4 | Recreation |
| Browning | BLR Lightweight Stainless w pistol grip | Rifle | 7mm-08 Rem | 20 | 5 | Recreation |
| Browning | BLR LIghtweight with Pistol Grip | Rifle | 22-250 Rem | 20 | 5 | Recreation |
| Browning | BLR LIghtweight with Pistol Grip | Rifle | 223 Rem | 20 | 5 | Recreation |
| Browning | BLR LIghtweight with Pistol Grip | Rifle | 243 Win | 20 | 5 | Recreation |
| Browning | BLR LIghtweight with Pistol Grip | Rifle | 270 Win | 22 | 5 | Recreation |
| Browning | BLR LIghtweight with Pistol Grip | Rifle | 270 WSM | 22 | 4 | Recreation |
| Browning | BLR LIghtweight with Pistol Grip | Rifle | 300 Win Mag | 24 | 4 | Recreation |
| Browning | BLR LIghtweight with Pistol Grip | Rifle | 300 WSM | 22 | 4 | Recreation |
| Browning | BLR LIghtweight with Pistol Grip | Rifle | 30-06 Sprg | 22 | 5 | Recreation |
| Browning | BLR LIghtweight with Pistol Grip | Rifle | 308 Win | 20 | 5 | Recreation |
| Browning | BLR LIghtweight with Pistol Grip | Rifle | 358 Win | 20 | 5 | Recreation |
| Browning | BLR LIghtweight with Pistol Grip | Rifle | 450 Marlin | 20 | 4 | Recreation |
| Browning | BLR LIghtweight with Pistol Grip | Rifle | 7mm Rem Mag | 24 | 4 | Recreation |
| Browning | BLR LIghtweight with Pistol Grip | Rifle | 7mm-08 Rem | 20 | 5 | Recreation |
| Browning | Buck Mark Sporter | Rifle | 22LR | 18 | 11 | Recreation |
| Browning | Buck Mark Target | Rifle | 22LR | 18 | 11 | Recreation |
| Browning | Buck Mark Target Gray Laminate, FLD | Rifle | 22LR | 18 | 11 | Recreation |
| Browning | SA-22 Grade I | Rifle | 22LR | 19-3/8 | 11 | Recreation |
| Browning | SA-22 Grade VI Blued | Rifle | 22LR | 19-3/8 | 11 | Recreation |
| Browning | SA-22 Grade VI Grayed | Rifle | 22LR | 19-3/8 | 11 | Recreation |
| Browning | T-Bolt Composite Sporter | Rifle | 17 HMR | 22 | 11 | Recreation |
| Browning | T-Bolt Composite Sporter | Rifle | 22 LR | 22 | 11 | Recreation |
| Browning | T-Bolt Composite Sporter | Rifle | 22 WMR | 22 | 11 | Recreation |
| Browning | T-Bolt Composite Target/Varmint | Rifle | 17 HMR | 22 | 11 | Recreation |
| Browning | T-Bolt Composite Target/Varmint | Rifle | 22 LR | 22 | 11 | Recreation |
| Browning | T-Bolt Composite Target/Varmint | Rifle | 22 WMR | 22 | 11 | Recreation |
| Browning | T-Bolt Gray Laminated Target/Varmint Stainless | Rifle | 17 HMR | 22 | 11 | Recreation |
| Browning | T-Bolt Gray Laminated Target/Varmint Stainless | Rifle | 22 WMR | 22 | 11 | Recreation |
| Browning | T-Bolt Gray Laminated Target/Varmint Stainless | Rifle | 22LR | 22 | 11 | Recreation |
| Browning | T-Bolt Sporter | Rifle | 17 HMR | 22 | 11 | Recreation |
| Browning | T-Bolt Sporter | Rifle | 22 LR | 22 | 11 | Recreation |
| Browning | T-Bolt Sporter | Rifle | 22 WMR | 22 | 11 | Recreation |
| Browning | T-Bolt Sporter, left-hand | Rifle | 17 HMR | 22 | 11 | Recreation |
| Browning | T-Bolt Sporter, left-hand | Rifle | 22 LR | 22 | 11 | Recreation |
| Browning | T-Bolt Sporter, left-hand | Rifle | 22 WMR | 22 | 11 | Recreation |
| Browning | T-Bolt Target/Varmint | Rifle | 17 HMR | 22 | 11 | Recreation |
| Browning | T-Bolt Target/Varmint | Rifle | 22 LR | 22 | 11 | Recreation |
| Browning | T-Bolt Target/Varmint | Rifle | 22 WMR | 22 | 11 | Recreation |
| Browning | T-Bolt Target/Varmint, left-hand | Rifle | 17 HMR | 22 | 11 | Recreation |
| Browning | T-Bolt Target/Varmint, left-hand | Rifle | 22 LR | 22 | 11 | Recreation |
| Browning | T-Bolt Target/Varmint, left-hand | Rifle | 22 WMR | 22 | 11 | Recreation |
| Browning | X-Bolt Composite Stalker | Rifle | 22-250 Rem | 22 | 5 | Recreation |
| Browning | X-Bolt Composite Stalker | Rifle | 223 Rem | 22 | 6 | Recreation |
| Browning | X-Bolt Composite Stalker | Rifle | 243 Win | 22 | 5 | Recreation |
| Browning | X-Bolt Composite Stalker | Rifle | 25-06 Rem | 24 | 5 | Recreation |
| Browning | X-Bolt Composite Stalker | Rifle | 270 Win | 22 | 5 | Recreation |
| Browning | X-Bolt Composite Stalker | Rifle | 270 WSM | 23 | 4 | Recreation |
| Browning | X-Bolt Composite Stalker | Rifle | 280 Rem | 22 | 5 | Recreation |
| Browning | X-Bolt Composite Stalker | Rifle | 300 Win Mag | 26 | 4 | Recreation |
| Browning | X-Bolt Composite Stalker | Rifle | 300 WSM | 23 | 4 | Recreation |
| Browning | X-Bolt Composite Stalker | Rifle | 30-06 Sprg | 22 | 5 | Recreation |
| Browning | X-Bolt Composite Stalker | Rifle | 308 Win | 22 | 5 | Recreation |
| Browning | X-Bolt Composite Stalker | Rifle | 338 Win Mag | 26 | 4 | Recreation |
| Browning | X-Bolt Composite Stalker | Rifle | 6.5 Creedmoor | 22 | 5 | Recreation |
| Browning | X-Bolt Composite Stalker | Rifle | 6mm Creedmoor | 22 | 5 | Recreation |
| Browning | X-Bolt Composite Stalker | Rifle | 7mm Rem Mag | 26 | 4 | Recreation |
| Browning | X-Bolt Composite Stalker | Rifle | 7mm-08 Rem | 22 | 5 | Recreation |
| Browning | X-Bolt Eclipse Hunter | Rifle | 243 Win | 24 | 5 | Recreation |
| Browning | X-Bolt Eclipse Hunter | Rifle | 25-06 Rem | 24 | 5 | Recreation |
| Browning | X-Bolt Eclipse Hunter | Rifle | 270 Win | 24 | 5 | Recreation |
| Browning | X-Bolt Eclipse Hunter | Rifle | 270 WSM | 24 | 4 | Recreation |
| Browning | X-Bolt Eclipse Hunter | Rifle | 300 Win Mag | 26 | 4 | Recreation |
| Browning | X-Bolt Eclipse Hunter | Rifle | 300 WSM | 24 | 4 | Recreation |
| Browning | X-Bolt Eclipse Hunter | Rifle | 30-06 Sprg | 24 | 5 | Recreation |
| Browning | X-Bolt Eclipse Hunter | Rifle | 308 Win | 24 | 5 | Recreation |
| Browning | X-Bolt Eclipse Hunter | Rifle | 6.5 Creedmoor | 24 | 5 | Recreation |
| Browning | X-Bolt Eclipse Hunter | Rifle | 6mm Creedmoor | 24 | 5 | Recreation |
| Browning | X-Bolt Eclipse Hunter | Rifle | 7mm Rem Mag | 26 | 4 | Recreation |
| Browning | X-Bolt Eclipse Hunter | Rifle | 7mm-08 Rem | 24 | 5 | Recreation |
| Browning | X-Bolt Eclipse Target | Rifle | 308 Win | 26 | 5 | Recreation |
| Browning | X-Bolt Eclipse Target | Rifle | 6.5 Creedmoor | 26 | 5 | Recreation |
| Browning | X-Bolt Eclipse Target | Rifle | 6mm Creedmoor | 26 | 5 | Recreation |
| Browning | X-Bolt Eclipse Varmint | Rifle | 204 Rgr | 26 | 6 | Recreation |
| Browning | X-Bolt Eclipse Varmint | Rifle | 22-250 Rem | 26 | 5 | Recreation |
| Browning | X-Bolt Eclipse Varmint | Rifle | 223 Rem | 26 | 6 | Recreation |
| Browning | X-Bolt Hell's Canyon Long Range | Rifle | 26 Nosler | 26 | 4 | Recreation |
| Browning | X-Bolt Hell's Canyon Long Range | Rifle | 270 WSM | 26 | 4 | Recreation |
| Browning | X-Bolt Hell's Canyon Long Range | Rifle | 28 Nosler | 26 | 4 | Recreation |
| Browning | X-Bolt Hell's Canyon Long Range | Rifle | 300 Win Mag | 26 | 4 | Recreation |
| Browning | X-Bolt Hell's Canyon Long Range | Rifle | 300 WSM | 26 | 4 | Recreation |
| Browning | X-Bolt Hell's Canyon Long Range | Rifle | 6.5 Creedmoor | 26 | 5 | Recreation |
| Browning | X-Bolt Hell's Canyon Long Range | Rifle | 6mm Creedmoor | 26 | 5 | Recreation |
| Browning | X-Bolt Hell's Canyon Long Range | Rifle | 7mm Rem Mag | 26 | 4 | Recreation |
| Browning | X-Bolt Hell's Canyon Speed | Rifle | 243 Win | 22 | 5 | Recreation |
| Browning | X-Bolt Hell's Canyon Speed | Rifle | 26 Nosler | 26 | 4 | Recreation |
| Browning | X-Bolt Hell's Canyon Speed | Rifle | 270 Win | 22 | 5 | Recreation |
| Browning | X-Bolt Hell's Canyon Speed | Rifle | 270 WSM | 23 | 4 | Recreation |
| Browning | X-Bolt Hell's Canyon Speed | Rifle | 28 Nosler | 26 | 4 | Recreation |
| Browning | X-Bolt Hell's Canyon Speed | Rifle | 300 Win Mag | 26 | 4 | Recreation |
| Browning | X-Bolt Hell's Canyon Speed | Rifle | 300 WSM | 23 | 4 | Recreation |
| Browning | X-Bolt Hell's Canyon Speed | Rifle | 30-06 Sprig | 22 | 5 | Recreation |
| Browning | X-Bolt Hell's Canyon Speed | Rifle | 308 Win | 22 | 5 | Recreation |
| Browning | X-Bolt Hell's Canyon Speed | Rifle | 6.5 Creedmoor | 22 | 5 | Recreation |
| Browning | X-Bolt Hell's Canyon Speed | Rifle | 6mm Creedmoor | 22 | 5 | Recreation |
| Browning | X-Bolt Hell's Canyon Speed | Rifle | 7mm Rem Mag | 26 | 4 | Recreation |
| Browning | X-Bolt Hell's Canyon Speed | Rifle | 7mm-08 Rem | 22 | 5 | Recreation |
| Browning | X-Bolt Hell's Canyon Speed Long Range McMillan | Rifle | 26 Nosler | 26 | 3 | Recreation |
| Browning | X-Bolt Hell's Canyon Speed Long Range McMillan | Rifle | 28 Nosler | 26 | 3 | Recreation |
| Browning | X-Bolt Hell's Canyon Speed Long Range McMillan | Rifle | 300 Win Mag | 26 | 3 | Recreation |
| Browning | X-Bolt Hell's Canyon Speed Long Range McMillan | Rifle | 300 WSM | 26 | 3 | Recreation |
| Browning | X-Bolt Hell's Canyon Speed Long Range McMillan | Rifle | 6.5 Creedmoor | 26 | 4 | Recreation |
| Browning | X-Bolt Hell's Canyon Speed Long Range McMillan | Rifle | 6mm Creedmoor | 26 | 4 | Recreation |
| Browning | X-Bolt Hell's Canyon Speed Long Range McMillan | Rifle | 7mm Rem Mag | 26 | 3 | Recreation |
| Browning | X-Bolt Hunter | Rifle | 22-250 Rem | 22 | 5 | Recreation |
| Browning | X-Bolt Hunter | Rifle | 223 Rem | 22 | 6 | Recreation |
| Browning | X-Bolt Hunter | Rifle | 243 Win | 22 | 5 | Recreation |
| Browning | X-Bolt Hunter | Rifle | 25-06 Rem | 24 | 5 | Recreation |
| Browning | X-Bolt Hunter | Rifle | 270 Win | 22 | 5 | Recreation |
| Browning | X-Bolt Hunter | Rifle | 270 WSM | 23 | 4 | Recreation |
| Browning | X-Bolt Hunter | Rifle | 280 Rem | 22 | 5 | Recreation |
| Browning | X-Bolt Hunter | Rifle | 300 Win Mag | 26 | 4 | Recreation |
| Browning | X-Bolt Hunter | Rifle | 300 WSM | 23 | 4 | Recreation |
| Browning | X-Bolt Hunter | Rifle | 30-06 Sprg | 22 | 5 | Recreation |
| Browning | X-Bolt Hunter | Rifle | 308 Win | 22 | 5 | Recreation |
| Browning | X-Bolt Hunter | Rifle | 338 Win Mag | 26 | 4 | Recreation |
| Browning | X-Bolt Hunter | Rifle | 375 H&H Mag | 24 | 4 | Recreation |
| Browning | X-Bolt Hunter | Rifle | 6.5 Creedmoor | 22 | 5 | Recreation |
| Browning | X-Bolt Hunter | Rifle | 6mm Creedmoor | 22 | 5 | Recreation |
| Browning | X-Bolt Hunter | Rifle | 7mm Rem Mag | 26 | 4 | Recreation |
| Browning | X-Bolt Hunter | Rifle | 7mm-08 Rem | 22 | 5 | Recreation |
| Browning | X-Bolt Hunter Left-hand | Rifle | 22-250 Rem | 22 | 5 | Recreation |
| Browning | X-Bolt Hunter Left-hand | Rifle | 223 Rem | 22 | 6 | Recreation |
| Browning | X-Bolt Hunter Left-hand | Rifle | 243 Win | 22 | 5 | Recreation |
| Browning | X-Bolt Hunter Left-hand | Rifle | 25-06 Rem | 24 | 5 | Recreation |
| Browning | X-Bolt Hunter Left-hand | Rifle | 270 Win | 22 | 5 | Recreation |
| Browning | X-Bolt Hunter Left-hand | Rifle | 270 WSM | 23 | 4 | Recreation |
| Browning | X-Bolt Hunter Left-hand | Rifle | 300 Win Mag | 26 | 4 | Recreation |
| Browning | X-Bolt Hunter Left-hand | Rifle | 300 WSM | 23 | 4 | Recreation |
| Browning | X-Bolt Hunter Left-hand | Rifle | 30-06 Sprg | 22 | 5 | Recreation |
| Browning | X-Bolt Hunter Left-hand | Rifle | 308 Win | 22 | 5 | Recreation |
| Browning | X-Bolt Hunter Left-hand | Rifle | 6.5 Creedmoor | 22 | 5 | Recreation |
| Browning | X-Bolt Hunter Left-hand | Rifle | 7mm Rem Mag | 26 | 4 | Recreation |
| Browning | X-Bolt Hunter Left-hand | Rifle | 7mm-08 Rem | 22 | 5 | Recreation |
| Browning | X-Bolt Med, Left Hand | Rifle | 243 Win | 22 | 5 | Recreation |
| Browning | X-Bolt Med, Left Hand | Rifle | 270 Win | 22 | 5 | Recreation |
| Browning | X-Bolt Med, Left Hand | Rifle | 270 WSM | 23 | 4 | Recreation |
| Browning | X-Bolt Med, Left Hand | Rifle | 300 Win Mag | 26 | 4 | Recreation |
| Browning | X-Bolt Med, Left Hand | Rifle | 300 WSM | 23 | 4 | Recreation |
| Browning | X-Bolt Med, Left Hand | Rifle | 30-06 Sprg | 22 | 5 | Recreation |
| Browning | X-Bolt Med, Left Hand | Rifle | 308 Win | 22 | 5 | Recreation |
| Browning | X-Bolt Med, Left Hand | Rifle | 6.5 Creedmoor | 22 | 5 | Recreation |
| Browning | X-Bolt Med, Left Hand | Rifle | 7mm Rem Mag | 26 | 4 | Recreation |
| Browning | X-Bolt Medallion | Rifle | 22-250 Rem | 22 | 5 | Recreation |
| Browning | X-Bolt Medallion | Rifle | 223 Rem | 22 | 6 | Recreation |
| Browning | X-Bolt Medallion | Rifle | 243 Win | 22 | 5 | Recreation |
| Browning | X-Bolt Medallion | Rifle | 25-06 Rem | 24 | 5 | Recreation |
| Browning | X-Bolt Medallion | Rifle | 25-06 Rem | 24 | 5 | Recreation |
| Browning | X-Bolt Medallion | Rifle | 270 Win | 22 | 5 | Recreation |
| Browning | X-Bolt Medallion | Rifle | 270 WSM | 23 | 4 | Recreation |
| Browning | X-Bolt Medallion | Rifle | 270 WSM | 23 | 4 | Recreation |
| Browning | X-Bolt Medallion | Rifle | 280 Rem | 22 | 5 | Recreation |
| Browning | X-Bolt Medallion | Rifle | 300 Win Mag | 26 | 4 | Recreation |
| Browning | X-Bolt Medallion | Rifle | 300 WSM | 23 | 4 | Recreation |
| Browning | X-Bolt Medallion | Rifle | 300 WSM | 23 | 4 | Recreation |
| Browning | X-Bolt Medallion | Rifle | 30-06 Sprg | 22 | 5 | Recreation |
| Browning | X-Bolt Medallion | Rifle | 308 Win | 22 | 5 | Recreation |
| Browning | X-Bolt Medallion | Rifle | 338 Win Mag | 26 | 4 | Recreation |
| Browning | X-Bolt Medallion | Rifle | 375 H&H Mag | 24 | 4 | Recreation |
| Browning | X-Bolt Medallion | Rifle | 6.5mm Creedmoor | 22 | 5 | Recreation |
| Browning | X-Bolt Medallion | Rifle | 6mm Creedmoor | 22 | 5 | Recreation |
| Browning | X-Bolt Medallion | Rifle | 7mm Rem Mag | 26 | 4 | Recreation |
| Browning | X-Bolt Micro Composite | Rifle | 243 Win | 20 | 5 | Recreation |
| Browning | X-Bolt Micro Composite | Rifle | 308 Win | 20 | 5 | Recreation |
| Browning | X-Bolt Micro Composite | Rifle | 6.5 Creedmoor | 20 | 5 | Recreation |
| Browning | X-Bolt Micro Composite | Rifle | 6mm Creedmoor | 20 | 5 | Recreation |
| Browning | X-Bolt Micro Composite | Rifle | 7mm-08 Rem | 20 | 5 | Recreation |
| Browning | X-Bolt Micro Midas | Rifle | 22-250 Rem | 20 | 5 | Recreation |
| Browning | X-Bolt Micro Midas | Rifle | 243 Win | 20 | 5 | Recreation |
| Browning | X-Bolt Micro Midas | Rifle | 308 Win | 20 | 5 | Recreation |
| Browning | X-Bolt Micro Midas | Rifle | 6.5mm Creedmoor | 20 | 5 | Recreation |
| Browning | X-Bolt Micro Midas | Rifle | 6mm Creedmoor | 20 | 5 | Recreation |
| Browning | X-Bolt Micro Midas | Rifle | 7mm-08 Re, | 20 | 5 | Recreation |
| Browning | X-Bolt Micro Midas, Left Hand | Rifle | 243 Win | 20 | 5 | Recreation |
| Browning | X-Bolt Micro Midas, Left Hand | Rifle | 308 Win | 20 | 5 | Recreation |
| Browning | X-Bolt Micro Midas, Left Hand | Rifle | 6.5 Creedmoor | 20 | 5 | Recreation |
| Browning | X-Bolt Micro Midas, Left Hand | Rifle | 7mm-08 Rem | 20 | 5 | Recreation |
| Browning | X-Bolt pro | Rifle | 26 Nosler | 26 | 3 | Recreation |
| Browning | X-Bolt pro | Rifle | 270 Win | 22 | 5 | Recreation |
| Browning | X-Bolt pro | Rifle | 28 Nosler | 26 | 4 | Recreation |
| Browning | X-Bolt pro | Rifle | 300 Win Mag | 26 | 4 | Recreation |
| Browning | X-Bolt pro | Rifle | 300 WSM | 23 | 4 | Recreation |
| Browning | X-Bolt pro | Rifle | 30-06 Sprg | 22 | 5 | Recreation |
| Browning | X-Bolt pro | Rifle | 308 Win | 22 | 5 | Recreation |
| Browning | X-Bolt pro | Rifle | 6.5 Creedmoor | 22 | 5 | Recreation |
| Browning | X-Bolt pro | Rifle | 6mm Creedmoor | 22 | 5 | Recreation |
| Browning | X-Bolt pro | Rifle | 7mm Rem Mag | 26 | 4 | Recreation |
| Browning | X-Bolt pro long range | Rifle | 26 Nosler | 26 | 4 | Recreation |
| Browning | X-Bolt pro long range | Rifle | 270 WSM | 26 | 4 | Recreation |
| Browning | X-Bolt pro long range | Rifle | 28 Nosler | 26 | 4 | Recreation |
| Browning | X-Bolt pro long range | Rifle | 300 Win Mag | 26 | 4 | Recreation |
| Browning | X-Bolt pro long range | Rifle | 300 WSM | 26 | 4 | Recreation |
| Browning | X-Bolt pro long range | Rifle | 6.5 Creedmoor | 26 | 5 | Recreation |
| Browning | X-Bolt pro long range | Rifle | 6mm Creedmoor | 26 | 5 | Recreation |
| Browning | X-Bolt pro long range | Rifle | 7mm Rem Mag | 26 | 4 | Recreation |
| Browning | X-Bolt RMEF | Rifle | 300 Win Mag | 26 | 4 | Recreation |
| Browning | X-Bolt Stainless Stalker | Rifle | 22-250 Rem | 22 | 5 | Recreation |
| Browning | X-Bolt Stainless Stalker | Rifle | 223 Rem | 22 | 6 | Recreation |
| Browning | X-Bolt Stainless Stalker | Rifle | 243 Win | 22 | 5 | Recreation |
| Browning | X-Bolt Stainless Stalker | Rifle | 25-06 Rem | 24 | 5 | Recreation |
| Browning | X-Bolt Stainless Stalker | Rifle | 270 Win | 22 | 5 | Recreation |
| Browning | X-Bolt Stainless Stalker | Rifle | 270 WSM | 23 | 4 | Recreation |
| Browning | X-Bolt Stainless Stalker | Rifle | 280 Rem | 22 | 5 | Recreation |
| Browning | X-Bolt Stainless Stalker | Rifle | 300 Win Mag | 26 | 4 | Recreation |
| Browning | X-Bolt Stainless Stalker | Rifle | 300 WSM | 23 | 4 | Recreation |
| Browning | X-Bolt Stainless Stalker | Rifle | 30-06 Sprg | 22 | 5 | Recreation |
| Browning | X-Bolt Stainless Stalker | Rifle | 308 Win | 22 | 5 | Recreation |
| Browning | X-Bolt Stainless Stalker | Rifle | 338 Win Mag | 26 | 4 | Recreation |
| Browning | X-Bolt Stainless Stalker | Rifle | 375 H&H Mag | 24 | 4 | Recreation |
| Browning | X-Bolt Stainless Stalker | Rifle | 6.5 Creedmoor | 22 | 5 | Recreation |
| Browning | X-Bolt Stainless Stalker | Rifle | 6mm Creedmoor | 22 | 5 | Recreation |
| Browning | X-Bolt Stainless Stalker | Rifle | 7mm Rem. Mag | 26 | 4 | Recreation |
| Browning | X-Bolt Stainless Stalker | Rifle | 7mm-08 Rem | 22 | 5 | Recreation |
| Browning | X-Bolt White Gold Medallion | Rifle | 22-250 Rem | 22 | 5 | Recreation |
| Browning | X-Bolt White Gold Medallion | Rifle | 223 Rem | 22 | 6 | Recreation |
| Browning | X-Bolt White Gold Medallion | Rifle | 243 Win | 22 | 5 | Recreation |
| Browning | X-Bolt White Gold Medallion | Rifle | 25-06 REm | 24 | 5 | Recreation |
| Browning | X-Bolt White Gold Medallion | Rifle | 270 WIn | 22 | 5 | Recreation |
| Browning | X-Bolt White Gold Medallion | Rifle | 270 WSM | 23 | 4 | Recreation |
| Browning | X-Bolt White Gold Medallion | Rifle | 280 Rem | 22 | 5 | Recreation |
| Browning | X-Bolt White Gold Medallion | Rifle | 300 Win Mag | 26 | 4 | Recreation |
| Browning | X-Bolt White Gold Medallion | Rifle | 300 WSM | 23 | 4 | Recreation |
| Browning | X-Bolt White Gold Medallion | Rifle | 30-06 Sprg | 22 | 5 | Recreation |
| Browning | X-Bolt White Gold Medallion | Rifle | 308 Win | 22 | 5 | Recreation |
| Browning | X-Bolt White Gold Medallion | Rifle | 338 Win Mag | 26 | 4 | Recreation |
| Browning | X-Bolt White Gold Medallion | Rifle | 6.5mm Creedmoor | 22 | 5 | Recreation |
| Browning | X-Bolt White Gold Medallion | Rifle | 6mm Creedmoor | 22 | 5 | Recreation |
| Browning | X-Bolt White Gold Medallion | Rifle | 7mm Rem Mag | 26 | 4 | Recreation |
| Browning | X-Bolt White Gold Medallion | Rifle | 7mm WSM | 23 | 4 | Recreation |
| Browning | X-Bolt White Gold Medallion | Rifle | 7mm-08 Rem | 22 | 5 | Recreation |
| Bushmaster | 10.5" ACR SBR | Rifle | 5.56 NATO | 27.00 | 30 | Tactical |
| Bushmaster | 16" 450 Bushmaster SD Carbine | Rifle | 450 Bushmaster | 36.50 | 5 | Recreation |
| Bushmaster | 16" Bushmaster 450 Carbine | Rifle | 450 Bushmaster | 35.50 | 5 | Recreation |
| Bushmaster | 16.5" ACR | Rifle | 5.56 NATO | 36.00 | 30 | Tactical |
| Bushmaster | 20" 450 Bushmaster SD Rifle | Rifle | 450 Bushmaster | 40.50 | 5 | Recreation |
| Bushmaster | 20" Bushmaster 450 Rifle | Rifle | 450 Bushmaster | 39.50 | 5 | Recreation |
| Bushmaster | 20" Predator Compliant | Rifle | 5.56 NATO | 38.25 | 10 | Recreation |
| Bushmaster | 24" Varmint Compliant | Rifle | 5.56 NATO | 42.25 | 10 | Recreation |
| Bushmaster | ACR DMR | Rifle | 5.56 NATO | 39.00 | 20 | Tactical |
| Bushmaster | BA 50 | Rifle | 50 BMG | 58.00 | 10 | Recreation |
| Bushmaster | Minimalist SD | Rifle | 5.56 NATO / 300 BLK | 35.00 | 30 | Tactical |
| Bushmaster | Minimalist SD (California compliant) | Rifle | 5.56 NATO / 300 BLK | 35.00 | 10 | Tactical |
| Bushmaster | MOE Series | Rifle | 5.56 NATO | 32.50 | 30 | Tactical |
| Bushmaster | XM-15 Series (16" 300 BLK ORC) | Rifle | 300 BLK | 35.00 | 30 | Tactical |
| Bushmaster | XM-15 Series (16" carbine) | Rifle | 5.56 NATO | 35.00 | 30 | Tactical |
| Bushmaster | XM-15 Series (16" optics) | Rifle | 5.56 NATO | 35.00 | 30 | Tactical |
| Bushmaster | XM-15 Series (20" target rifle) | Rifle | 5.56 NATO | 39.50 | 30 | Tactical |
| Bushmaster | XM-15 Series (XM10 ORC Gen 1 308) | Rifle | 7.62 NATO | 33.50 | 30 | Tactical |
| Century Arms | AK63DS Rifle | Rifle | 7.62x39mm | 35.75 | 30 | Tactical |
| Century Arms | C308 Rifle | Rifle | 308 Win | 40.20 | 20 | Tactical |
| Century Arms | C39v2 Rifle | Rifle | 7.62x39mm | 38.00 | 30 | Tactical |
| Century Arms | N-PAP DF Rifle | Rifle | 7.62x39mm | 34.25 | 30 | Tactical |
| Century Arms | N-PAP Rifle | Rifle | 7.62x39mm | 36.25 | 30 | Tactical |
| Century Arms | RAS-47 Rifle | Rifle | 7.62x39mm | 37.25 | 30 | Tactical |
| Century Arms | RH-10 Rifle | Rifle | 7.62x39mm | 37.25 | 30 | Tactical |
| Century Arms | WASR-10 Rifle | Rifle | 7.62x39mm | 34.25 | 30 | Tactical |
| Christensen Arms | BA Tactical | Rifle | Multiple | 41.50 | 5 | Tactical |
| Christensen Arms | CA-10 DMR | Rifle | Multiple |  | 20 | Tactical |
| Christensen Arms | CA-10 G2 | Rifle | 308 Win / 6.5 Creedmoor | 35.50 | 20 | Tactical |
| Christensen Arms | CA-15 G2 | Rifle | 223 Wylde |  | 5 | Tactical |
| Christensen Arms | Classic | Rifle | Multiple |  | 4 | Recreation |
| Christensen Arms | ELR | Rifle | Multiple |  | 4 | Recreation |
| Christensen Arms | Mesa | Rifle | Multiple |  | 4 | Recreation |
| Christensen Arms | Mesa Long Range | Rifle | Multiple |  | 4 | Recreation |
| Christensen Arms | Modern Precision Rifle | Rifle | Multiple | 46.63 | 10 | Tactical |
| Christensen Arms | Ridgeline | Rifle | Multiple |  | 4 | Recreation |
| Christensen Arms | Summit TI | Rifle | Multiple |  | 4 | Recreation |
| Christensen Arms | TFM (Tactical Force Multiplier) | Rifle | Multiple | 46.50 | 5 | Tactical |
| Colt | AR15A4 Rifle | Rifle | .223 Remington/5.56 NATO | 39.5" | 30 | Tactical |
| Colt | Colt AR-15 Semi-Auto 9MM | Rifle | 9MM | 35" | 32 | Tactical |
| Colt | Colt M4 Carbine | Rifle | 5.56 MM | 33" | 30 | Tactical |
| Colt | Colt M4 Monolithic | Rifle | .556 NATO | 35.5" | 30 | Tactical |
| Colt | Combat Unit Carbine | Rifle | 5.56 NATO | 36.5" | 30 | Tactical |
| Colt | Enhanced Patrol Rifle | Rifle | .556 NATO | 35.5" | 30 | Tactical |
| Colt | Lightweight Carbine | Rifle | .223 Remington/ 5.56 NATO | 35" | 30 | Tactical |
| Colt | M16A1 Retro Reissue | Rifle | 5.56MM | 38.8" | 20 | Tactical |
| Colt | M4 Carbine Magpul SL Black | Rifle | .223 Remington/ 5.56 NATO | 35.5" | 30 | Tactical |
| Colt | M4 Carbine OEM1 | Rifle | .556 NATO | 32" | 30 | Tactical |
| Colt | M4 Carbine OEM2 | Rifle | .556 NATO | 35.5" | 30 | Tactical |
| Colt | M4A1 SOCOM | Rifle | .223 Remington/ 5.56 NATO | 35.5" | 30 | Tactical |
| Colt | Modular Carbine | Rifle | .223 Remington/ 5.56 NATO | 36.75" | 20 | Tactical |
| Colt | XM177E2 Retro Carbine | Rifle | 5.56MM | 34" | 20 | Tactical |
| Daniel Defense | AMBUSH | Rifle | 5.56 NATO / 300 BLK / 308 Win / 6.8mm SPC | 39.00 | 5 | Recreation |
| Daniel Defense | DD5 | Rifle | 308 Win | 39.00 | 20 | Tactical |
| Daniel Defense | DDM4 (not including AMBUSH) | Rifle | 5.56 NATO / 300 BLK | 37.88 | 32 | Tactical |
| Daniel Defense | M4A1 | Rifle | 5.56 NATO | 34.75 | 32 | Tactical |
| Daniel Defense | MK12 | Rifle | 5.56 NATO | 37.88 | 32 | Tactical |
| Daniel Defense | MK18 | Rifle | 5.56 NATO | 30.25 | 32 | Tactical |
| Desert Tech | HTI | Rifle | Multiple | 45.75 | 5 | Tactical |
| Desert Tech | MDR | Rifle | 223 Wylde / 308 Win / 300 BLK | 26.20 | 30 | Tactical |
| Desert Tech | SRS-A1 | Rifle | Multiple | 38.50 | 6 | Tactical |
| DRD Tactical | APTUS | Rifle | .556 / 300 BLK | 27.50 | 30 | Tactical |
| DRD Tactical | CDR-15 | Rifle | .556 / 300 BLK | 32.75 | 30 | Tactical |
| DRD Tactical | KIVAARI | Rifle | .338 Lapua | 48.00 | 10 | Tactical |
| DRD Tactical | M762 | Rifle | 7.62 NATO / 6.5 Creedmoor | 39.75 | 20 | Tactical |
| DRD Tactical | Paratus | Rifle | 7.62 NATO / 6.5 Creedmoor | 33.00 | 20 | Tactical |
| Eagle Arms | Eagle-15 | Rifle | .223 WYLDE | 35.50 | 30 | Tactical |
| Eagle Arms | Eagle-15 MFT | Rifle | .223 WYLDE | 35.90 | 30 | Tactical |
| Eagle Arms | Eagle-15 ORC | Rifle | .223 WYLDE | 35.50 | 30 | Tactical |
| F&D Defense | FD 260 | Rifle | 260 Rem |  | 20 | Tactical |
| F&D Defense | FD 308 | Rifle | 308 Win |  | 20 | Tactical |
| F&D Defense | FD 338 | Rifle | 338 Lapua Mag |  | 10 | Tactical |
| F&D Defense | FD 6.5 Creedmoor | Rifle | 6.5 Creedmoor |  | 20 | Tactical |
| F&D Defense | XAR | Rifle | 5.56 NATO / 223 Rem |  | 20 | Tactical |
| FN America | FN BALLISTA | Rifle | .338 Lapua | 50.75 | 8 | Recreation |
| FN America | FN M249S | Rifle | 5.56x45mm | 41.00 | 30 | Tactical |
| FN America | FN M249S PARA | Rifle | 5.56x45mm | 37.00 | 30 | Tactical |
| FN America | FN PS90 | Rifle | 5.7x28mm | 26.23 | 50 | Tactical |
| FN America | FN SCAR 16S | Rifle | 5.56x45mm | 37.50 | 30 | Tactical |
| FN America | FN SCAR 17S | Rifle | 7.62x51mm | 38.50 | 20 | Tactical |
| FN America | FN SCAR 20S | Rifle | 7.62x51mm | 42.50 | 10 | Tactical |
| FN America | FN15 DMR2 | Rifle | 5.56x45mm | 38.00 | 30 | Tactical |
| FN America | FN15 M16 | Rifle | 5.56x45mm | 39.70 | 30 | Tactical |
| FN America | FN15 M4 | Rifle | 5.56x45mm | 34.00 | 30 | Tactical |
| FN America | FN15 Patrol | Rifle | 5.56x45mm | 35.20 | 30 | Tactical |
| FN America | FN15 State Compliant | Rifle | 5.56 NATO | 38.75 | 10 | Tactical |
| FN America | FN15 Tactical .300 BLK II | Rifle | 7.62x35mm / 300 BLK | 37.25 | 30 | Tactical |
| FN America | FN15 Tactical Carbine | Rifle | 5.56x45mm | 37.25 | 30 | Tactical |
| FN America | FN15 Tactical II | Rifle | 5.56x45mm | 37.00 | 30 | Tactical |
| Heckler & Koch | HK416 22 LR Rifle | Rifle | 22 LR | 35.25 | 30 | Recreation |
| Heckler & Koch | MR556A1 | Rifle | 5.56mm NATO / 223 Rem | 37.68 | 30 | Tactical |
| Heckler & Koch | MR762A1 | Rifle | 7.62mm NATO / 308 Win | 39.10 | 20 | Tactical |
| Henry USA | Big Boy Carbine Color Case | Rifle | .44 Magnum/.44 Spl, .357 Magnum/.38 Spl, .45 Colt | 34.1" | 7 | Recreation |
| Henry USA | Big Boy Color Case | Rifle | .44 Magnum/.44 Spl, .357 Magnum/.38 Spl, .45 Colt | 37.5" | 10 | Recreation |
| Henry USA | Long Ranger | Rifle | .223 Rem/5.56 NATO | 40.5" | 5 | Recreation |
| Henry USA | Long Ranger | Rifle | 6.5 Creedmoor | 42.5 | 4 | Recreation |
| Henry USA | Long Ranger | Rifle | Multiple | 40.5 | 4 | Recreation |
| Henry USA | Single Shot Rifle | Rifle | Multiple | 37.5 | 1 | Recreation |
| Hi-Point Firearms | 1095TS | Rifle | 10MM | 32" | 10 | Recreation |
| Hi-Point Firearms | 3895TS | Rifle | 380ACP | 31" | 10 | Recreation |
| Hi-Point Firearms | 4095TS | Rifle | 40 S and W | 32" | 10 | Recreation |
| Hi-Point Firearms | 4595TS | Rifle | 45ACP | 32" | 9 | Recreation |
| Hi-Point Firearms | 995TS | Rifle | 9MM | 31" | 10 | Recreation |
| Hi-Point Firearms | California Compliant 40SW, 10MM | Rifle | 40SW, 10MM | 32" | 10 | Recreation |
| Hi-Point Firearms | California Compliant 45 AUTO | Rifle | 45 AUTO | 32" | 9 | Recreation |
| Hi-Point Firearms | California Compliant 9MM | Rifle | 9MM | 31" | 10 | Recreation |
| Hi-Point Firearms | Hunter Series 40S and W | Rifle | 40 S and W | 32" | 10 | Recreation |
| Hi-Point Firearms | Hunter Series 45ACP | Rifle | 45ACP | 32" | 9 | Recreation |
| Hi-Point Firearms | Hunter Series 9MM | Rifle | 9MM | 31" | 10 | Recreation |
| Hi-Point Firearms | OD or FDE | Rifle | 45ACP | 32" | 9 | Recreation |
| Hi-Point Firearms | OD or FDE | Rifle | 9MM | 31" | 10 | Recreation |
| JP Enterprises | CTR-02 | Rifle | Multiple |  | 30 | Tactical |
| JP Enterprises | GMR-13 | Rifle | 9mm |  | 10 | Tactical |
| JP Enterprises | GMR-15 | Rifle | 9mm |  | 10 | Tactical |
| JP Enterprises | JP-15 | Rifle | Multiple | 38.00 | 30 | Tactical |
| JP Enterprises | LRP-07 | Rifle | 308 Win / 260 Rem / 6.5 Creedmoor / 6.0 Creedmoor |  | 10 | Tactical |
| JP Enterprises | PSC-11 | Rifle | .223 Wylde / 5.56 NATO / 300 BLK / 6.5 Grendel / .224 Valkyrie | 40.40 | 30 | Tactical |
| JP Enterprises | PSC-12 | Rifle | 308 Win / 260 Rem / 6.5 Creedmoor / 6.0 Creedmoor |  | 30 | Tactical |
| JP Enterprises | SCR-11 | Rifle | Multiple | 40.75 | 30 | Recreation |
| Kel-Tec | CMR-30 | Rifle | 22WMR | 29.90 | 30 | Recreation |
| Kel-Tec | RDB - C | Rifle | 5.56mm NATO / 223 Rem | 30.40 | 10 | Tactical |
| Kel-Tec | RDB - S | Rifle | 5.56mm NATO / 223 Rem | 26.10 | 20 | Tactical |
| Kel-Tec | RDB 17" | Rifle | 5.56mm NATO / 223 Rem | 27.30 | 20 | Tactical |
| Kel-Tec | RFB 18" | Rifle | 7.62mm NATO / 308 Win | 26.00 | 20 | Tactical |
| Kel-Tec | RFB 24" | Rifle | 7.62mm NATO / 308 Win | 35.90 | 20 | Recreation |
| Kel-Tec | SU-16 Series | Rifle | 5.56mm NATO / 223 Rem | 37.40 | 10 | Tactical |
| Kel-Tec | SU-22 Series | Rifle | 22 LR | 34.00 | 26 | Recreation |
| Kel-Tec | SUB-2000 | Rifle | 9mm / 40 S&W | 30.50 | 30 | Tactical |
| Kimber | Adirondack | Rifle | Multiple | 37.25 | 4 | Recreation |
| Kimber | Advanced Tactical SOC | Rifle | 308 Win / 6.5 Creedmoor | 43.00 | 5 | Tactical |
| Kimber | Advanced Tactical SRC | Rifle | 308 Win | 37.00 | 5 | Tactical |
| Kimber | Caprivi | Rifle | Multiple | 44.50 | 4 | Recreation |
| Kimber | Classic Select Grade | Rifle | Multiple | 46.50 | 6 | Recreation |
| Kimber | Hunter | Rifle | Multiple | 43.75 | 3 | Recreation |
| Kimber | LongMaster Classic | Rifle | 223 Rem / 308 Win | 43.25 | 6 | Recreation |
| Kimber | LongMaster VT | Rifle | 22-250 Rem | 45.25 | 5 | Recreation |
| Kimber | LPT | Rifle | 223 Rem / 308 Win | 41.25 | 6 | Recreation |
| Kimber | Montana | Rifle | Multiple | 46.50 | 5 | Recreation |
| Kimber | Mountain Ascent | Rifle | Multiple | 46.50 | 4 | Recreation |
| Kimber | Open Country | Rifle | 6.5 Creedmoor / 308 Win | 43.25 | 4 | Recreation |
| Kimber | Patrol Tactical | Rifle | 308 Win / 300 Win Mag | 45.25 | 5 | Tactical |
| Kimber | Pro Varmint | Rifle | 204 Ruger / 223 Rem / 22-250 Rem | 43.25 | 6 | Recreation |
| Kimber | Subalpine | Rifle | Multiple | 46.50 | 4 | Recreation |
| Kimber | SuperAmerica | Rifle | 308 Win | 41.25 | 5 | Recreation |
| Kimber | SVT | Rifle | 223 Rem | 37.50 | 6 | Recreation |
| Kimber | Talkeetna | Rifle | 357 H&H Mag | 44.50 | 4 | Recreation |
| Kimber | Varmint | Rifle | 204 Ruger / 22-250 Rem | 45.25 | 6 | Recreation |
| Marlin | 444 Marlin | Rifle | 45-70 GOVT | 40.5 | 4 | Recreation |
| Marlin | Model 1894 | Rifle | 44 Remington Magnum/44 S&W spl, 45 colt | 38.5 | 10 | Recreation |
| Marlin | Model 1894C | Rifle | 38 SPL/357 Mag | 38.5 | 10 | Recreation |
| Marlin | Model 1894CB | Rifle | 38 SPL/357 Mag, 45 Colt, 44 Remingotn Magnum | 38.5 | 10 | Recreation |
| Marlin | Model 1894CSBL | Rifle | 38 SPL/357 Mag | 35 | 8 | Recreation |
| Marlin | Model 1894CST | Rifle | 38 SPL/357 Mag | 35 | 8 | Recreation |
| Marlin | Model 1894SBL | Rifle | 44 Remington Magnum/44 S&W Spl | 35 | 8 | Recreation |
| Marlin | Model 1895 | Rifle | 45-70 GOVT | 40.5 | 4 | Recreation |
| Marlin | Model 1895 CBA | Rifle | 45-70 GOVT | 37 | 6 | Recreation |
| Marlin | Model 1895CB | Rifle | 45-70 GOVT | 44.5 | 9 | Recreation |
| Marlin | Model 1895G | Rifle | 45-70 GOVT | 37 | 4 | Recreation |
| Marlin | Model 1895GBL | Rifle | 45-70 GOVT | 37 | 6 | Recreation |
| Marlin | Model 1895SBL | Rifle | 45-70 GOVT | 37 | 6 | Recreation |
| Marlin | Model 1895TSBL (Trapper) | Rifle | 45-70 GOVT | 35 | 6 | Recreation |
| Marlin | Model 336BL | Rifle | 30-30 WIN | 37 | 6 | Recreation |
| Marlin | Model 336C | Rifle | 30-30 WIN | 38.5 | 6 | Recreation |
| Marlin | Model 336SS | Rifle | 30-30 WIN | 38.5 | 6 | Recreation |
| Marlin | Model 336TDL | Rifle | 30-30 WIN | 38.5 | 6 | Recreation |
| Marlin | Model 336W | Rifle | 30-30 WIN | 38.5 | 6 | Recreation |
| Marlin | Model 336XLR | Rifle | 30-30 WIN | 42.5 | 5 | Recreation |
| Marlin | Model 336Y | Rifle | 30-30 WIN | 33.25 | 5 | Recreation |
| Marlin | Model 60 | Rifle | 22 Long Rifle | 37.5 | 14 | Recreation |
| Marlin | Model 60 SN | Rifle | 22 Long Rifle | 37.5 | 14 | Recreation |
| Marlin | Model 60 SS | Rifle | 22 Long Rifle | 37.5 | 14 | Recreation |
| Marlin | Model 60C | Rifle | 22 Long Rifle | 37.5 | 14 | Recreation |
| Marlin | Model 60SB | Rifle | 22 Long Rifle | 37.5 | 14 | Recreation |
| Marlin | Model 795 | Rifle | 22 Long Rifle | 37 | 10 | Recreation |
| Marlin | Model 7OPSS (Take-down) | Rifle | 22 Long Rifle | 35.25 | 7 | Recreation |
| Marlin | Model XT-22 | Rifle | 22 LR | 41 | 7 | Recreation |
| Marlin | Model XT-22M | Rifle | 22 WMR | 41 | 4 | Recreation |
| Marlin | Model XT-22M | Rifle | 22 WMR | 41 | 7 | Recreation |
| Marlin | Model XT-22MTSL | Rifle | 22 WMR | 41 | 12 | Recreation |
| Marlin | Model XT-22R | Rifle | 22 LR | 41 | 7 | Recreation |
| Marlin | Model XT-22R0 | Rifle | 22 LR | 41 | 7 | Recreation |
| Marlin | Model XT-22RZ | Rifle | 22 LR | 41 | 7 | Recreation |
| Marlin | Model XT-22TR | Rifle | 22 L | 41 | 19 | Recreation |
| Marlin | Model XT-22TR | Rifle | 22 LR | 41 | 17 | Recreation |
| Marlin | Model XT-22TR | Rifle | 22 S | 41 | 25 | Recreation |
| Marlin | Model XT-22TSR | Rifle | 22 L | 41 | 19 | Recreation |
| Marlin | Model XT-22TSR | Rifle | 22 LR | 41 | 17 | Recreation |
| Marlin | Model XT-22TSR | Rifle | 22 S | 41 | 25 | Recreation |
| Marlin | Model-XT17R | Rifle | 17 HMR | 41 | 4 | Recreation |
| Marlin | Model-XT17R | Rifle | 17 HMR | 41 | 7 | Recreation |
| Marlin | Model-XT17V | Rifle | 17 HMR | 41 | 4 | Recreation |
| Marlin | Model-XT17V | Rifle | 17 HMR | 41 | 7 | Recreation |
| Marlin | Model-XT17VR | Rifle | 17 HMR | 41 | 4 | Recreation |
| Marlin | Model-XT17VR | Rifle | 17 HMR | 41 | 7 | Recreation |
| Marlin | Model-XT17VSL | Rifle | 17 HMR | 41 | 4 | Recreation |
| Marlin | Model-XT17VSL | Rifle | 17 HMR | 41 | 7 | Recreation |
| Marlin | Model-XT22MR | Rifle | 22 WMR | 41 | 4 | Recreation |
| Marlin | Model-XT22MR | Rifle | 22 WMR | 41 | 7 | Recreation |
| Marlin | Model-XT22MSVR | Rifle | 22 WMR | 41 | 4 | Recreation |
| Marlin | Model-XT22MSVR | Rifle | 22 WMR | 41 | 7 | Recreation |
| Marlin | Model-XT22MTR | Rifle | 22 WMR | 41 | 12 | Recreation |
| Marlin | Model-XT22VR | Rifle | 22 LR | 41 | 7 | Recreation |
| Mossberg | 464 Centerfire Lever-Action | Rifle | 30-30 Win | 38.50 | 7 | Recreation |
| Mossberg | 464 Rimfire Lever-Action | Rifle | 22 LR | 35.75 | 14 | Recreation |
| Mossberg | 464 SPX Centerfire Lever-Action | Rifle | 30-30 Win | 34.00 | 6 | Recreation |
| Mossberg | 464 SPX Rimfire Lever-Action | Rifle | 22 LR | 35.50 | 14 | Recreation |
| Mossberg | Blaze-47 10-Round | Rifle | 22 LR | 35.75 | 11 | Recreation |
| Mossberg | Blaze-47 25-Round | Rifle | 22 LR | 35.75 | 26 | Recreation |
| Mossberg | International 702 Plinkster (10-round magazine) | Rifle | 22 LR | 37.00 | 11 | Recreation |
| Mossberg | International 702 Plinkster (25-round magazine) | Rifle | 22 LR | 37.00 | 26 | Recreation |
| Mossberg | International 715T Flat Top (10-round magazine) | Rifle | 22 LR | 33.00 | 11 | Recreation |
| Mossberg | International 715T Flat Top (25-round magazine) | Rifle | 22 LR | 33.00 | 26 | Recreation |
| Mossberg | International 802 Plinkster | Rifle | 22 LR | 37.00 | 11 | Recreation |
| Mossberg | International 817 - 17 HMR | Rifle | 17 HMR | 40.00 | 6 | Recreation |
| Mossberg | MMR Carbine - CA Compliant | Rifle | 5.56mm NATO / 223 Rem | 35.75 | 11 | Recreation |
| Mossberg | MMR Carbine (10-round magazine) | Rifle | 5.56mm NATO / 223 Rem | 36.50 | 11 | Recreation |
| Mossberg | MMR Carbine (30-round magazine) | Rifle | 5.56mm NATO / 223 Rem | 35.75 | 31 | Recreation |
| Mossberg | MMR Pro | Rifle | 5.56mm NATO / 223 Rem | 35.75 | 31 | Recreation |
| Mossberg | MMR Tactical | Rifle | 5.56mm NATO / 223 Rem | 33.00 | 31 | Tactical |
| Mossberg | Mossberg Patriot | Rifle | Multiple | 42.75 | 5 | Recreation |
| Mossberg | Mossberg Patriot Night Train | Rifle | 300 Win | 42.75 | 4 | Tactical |
| Mossberg | Mossberg Patriot Revere | Rifle | Multiple | 44.75 | 5 | Recreation |
| Mossberg | MVP Flex | Rifle | 5.56mm NATO / 223 Rem | 38.50 | 11 | Tactical |
| Mossberg | MVP Flex | Rifle | 7.62mm NATO / 308 Win | 37.75 | 11 | Tactical |
| Mossberg | MVP LC | Rifle | Multiple | 37.75 | 11 | Tactical |
| Mossberg | MVP LR | Rifle | Multiple | 41.50 | 11 | Tactical |
| Mossberg | MVP LR-T Tactical | Rifle | 5.56mm NATO / 223 Rem | 35.75 | 11 | Tactical |
| Mossberg | MVP LR-T Tactical | Rifle | 7.62mm NATO / 308 Win | 35.75 | 11 | Tactical |
| Mossberg | MVP Patrol | Rifle | 5.56mm NATO / 223 Rem | 37.00 | 11 | Tactical |
| Mossberg | MVP Patrol | Rifle | 7.62mm NATO / 308 Win | 37.50 | 11 | Tactical |
| Mossberg | MVP Precision | Rifle | 6.5 Creedmoor | 43.25 | 11 | Tactical |
| Mossberg | MVP Precision | Rifle | 7.62mm NATO / 308 Win | 39.25 | 11 | Tactical |
| Mossberg | MVP Predator | Rifle | 5.56mm NATO / 223 Rem | 37.50 | 11 | Recreation |
| Mossberg | MVP Predator | Rifle | 6.5 Creedmoor | 40.50 | 11 | Recreation |
| Mossberg | MVP Predator | Rifle | 7.62mm NATO / 308 Win | 38.50 | 11 | Recreation |
| Mossberg | MVP Scout | Rifle | 7.62mm NATO / 308 Win | 37.50 | 11 | Recreation |
| Mossberg | MVP Varmint | Rifle | Multiple | 44.00 | 11 | Recreation |
| Noreen Firearms | Bad News | Rifle | 338 Lapua |  | 10 | Tactical |
| Noreen Firearms | BBN223 | Rifle | 5.56 NATO |  | 30 | Tactical |
| Noreen Firearms | BN308 | Rifle | 308 Win |  | 20 | Recreation |
| Noreen Firearms | BN36 | Rifle | Multiple |  | 20 | Tactical |
| Noreen Firearms | BN408 | Rifle | 408 Chey Tac |  | 10 | Tactical |
| Noreen Firearms | ULR | Rifle | Multiple | 56.00 | 1 | Recreation |
| Palmetto State Armory | KS-47 Rifle | Rifle | 7.62x39mm |  | 30 | Tactical |
| Palmetto State Armory | PA-10 | Rifle | 308 Win / 6.5 Creedmoor | 40.00 | 20 | Tactical |
| Palmetto State Armory | PA-15 Rifle | Rifle | 5.56 NATO / .224 Valkyrie | 40.00 | 30 | Tactical |
| Palmetto State Armory | PA-9 Rifle | Rifle | 9mm | 32.00 | 31 | Tactical |
| Palmetto State Armory | PSAK-47 | Rifle | 7.62x39mm | 34.20 | 30 | Tactical |
| Remington | Model 552 | Rifle | 22S, 22L, 22 LR | 40.00 | 15 | Recreation |
| Remington | Model 572 | Rifle | 22S, 22L, 22 LR | 40.00 | 15 | Recreation |
| Remington | Model 597 | Rifle | 22 LR | 40.00 | 10 | Recreation |
| Remington | Model 700 ADL | Rifle | Multiple | 46.50 | 5 | Recreation |
| Remington | Model 700 ADL SS | Rifle | Multiple | 44.50 | 4 | Recreation |
| Remington | Model 700 AWR | Rifle | Multiple | 44.50 | 4 | Recreation |
| Remington | Model 700 BDL | Rifle | Multiple | 44.50 | 4 | Recreation |
| Remington | Model 700 CDL | Rifle | Multiple | 46.50 | 4 | Recreation |
| Remington | Model 700 CDL SF | Rifle | Multiple | 46.50 | 4 | Recreation |
| Remington | Model 700 Long Range | Rifle | Multiple | 47.00 | 4 | Recreation |
| Remington | Model 700 Magpul | Rifle | Multiple | 43.50 | 5 | Recreation |
| Remington | Model 700 Magpul Enhanced | Rifle | 308 Win / 6.5 Creedmoor | 39.50 | 10 | Recreation |
| Remington | Model 700 Mountain SS | Rifle | Multiple | 42.50 | 4 | Recreation |
| Remington | Model 700 PCR | Rifle | 260 Rem / 6.5 Creedmoor / 308 Win | 44.00 | 5 | Recreation |
| Remington | Model 700 Sendero SF II | Rifle | Multiple | 45.75 | 3 | Recreation |
| Remington | Model 700 SPS (Hunting) | Rifle | Multiple | 46.50 | 4 | Recreation |
| Remington | Model 700 SPS Camo | Rifle | Multiple | 44.50 | 4 | Recreation |
| Remington | Model 700 SPS Stainless (Hunting) | Rifle | Multiple | 46.50 | 5 | Recreation |
| Remington | Model 700 SPS Tactical | Rifle | Multiple | 41.75 | 5 | Recreation |
| Remington | Model 700 SPS Threaded Barrel | Rifle | Multiple | 40.50 | 5 | Recreation |
| Remington | Model 700 SPS Varmint | Rifle | Multiple | 46.50 | 5 | Recreation |
| Remington | Model 700 Stainless 5R | Rifle | 308 Win / 223 Rem / 300 Win Mag | 45.75 | 5 | Recreation |
| Remington | Model 700 Stainless 5R Gen 2 | Rifle | Multiple | 43.75 | 4 | Recreation |
| Remington | Model 700 Tactical Chassis | Rifle | 308 Win / 300 Win Mag / 338 Lapua | 50.00 | 5 | Recreation |
| Remington | Model 700 Ultimate Muzzleloader | Rifle | 50 Caliber | 47.00 | 1 | Recreation |
| Remington | Model 700 Varmint Laminated Stock (VLS) | Rifle | Multiple | 45.75 | 5 | Recreation |
| Remington | Model 700 Varmint SF | Rifle | Multiple | 45.75 | 5 | Recreation |
| Remington | Model 700 Varmint Target Rifle (VTR) | Rifle | Multiple | 41.70 | 5 | Recreation |
| Remington | Model 700 XCR Compact Tactical | Rifle | 308 Win | 39.75 | 4 | Recreation |
| Remington | Model 700 XCR Tactical Long Range | Rifle | 308 Win / 300 Win Mag / 338 Lapua Mag | 45.75 | 5 | Recreation |
| Remington | Model 7600 | Rifle | Multiple | 42.70 | 4 | Recreation |
| Remington | Model 783 | Rifle | Multiple | 44.50 | 5 | Recreation |
| Remington | Model 783 Heavy Barrel Threaded (HBT) | Rifle | Multiple | 36.12 | 5 | Recreation |
| Remington | Model Seven | Rifle | Multiple | 39.25 | 5 | Recreation |
| Ruger | 10/22 Carbine | Rifle | 22 LR | 37.00 | 10 | Recreation |
| Ruger | 10/22 Carbine | Rifle | 22 LR | 37.00 | 10 | Recreation |
| Ruger | 10/22 Carbine | Rifle | 22 LR | 37.00 | 10 | Recreation |
| Ruger | 10/22 Carbine | Rifle | 22 LR | 37.00 | 10 | Recreation |
| Ruger | 10/22 Sporter | Rifle | 22 LR | 37.00 | 10 | Recreation |
| Ruger | 10/22 Tactical | Rifle | 22 LR | 36.25 | 10 | Recreation |
| Ruger | 10/22 Takedown | Rifle | 22 LR | 37.00 | 10 | Recreation |
| Ruger | 10/22 Takedown | Rifle | 22 LR | 36.75 | 10 | Recreation |
| Ruger | 10/22 Takedown | Rifle | 22 LR | 34.62 | 10 | Recreation |
| Ruger | 10/22 Takedown Lite | Rifle | 22 LR | 34.62 | 10 | Recreation |
| Ruger | 10/22 Target Lite | Rifle | 22 LR | 35.25 | 10 | Recreation |
| Ruger | AR-556 | Rifle | 5.56 NATO / 223 Rem | 35.50 | 30 | Tactical |
| Ruger | AR-556 | Rifle | 5.56 NATO / 223 Rem | 35.50 | 30 | Tactical |
| Ruger | AR-556 MPR | Rifle | 5.56 NATO / 223 Rem | 38.25 | 30 | Tactical |
| Ruger | AR-556 State Compliant | Rifle | 5.56 NATO / 223 Rem | 34.40 | 10 | Tactical |
| Ruger | AR-556 State Compliant | Rifle | 5.56 NATO / 223 Rem | 35.50 | 10 | Tactical |
| Ruger | AR-556 State Compliant | Rifle | 5.56 NATO / 223 Rem | 32.85 | 10 | Tactical |
| Ruger | AR-556 with Monsterman Grip | Rifle | 5.56 NATO / 223 Rem | 32.85 | 10 | Tactical |
| Ruger | Hawkeye | Rifle | 204 Ruger | 44.00 | 5 | Recreation |
| Ruger | Hawkeye | Rifle | 223 Rem | 42.00 | 5 | Recreation |
| Ruger | Hawkeye | Rifle | 243 Win | 42.00 | 4 | Recreation |
| Ruger | Hawkeye | Rifle | 270 Win | 42.75 | 4 | Recreation |
| Ruger | Hawkeye | Rifle | 300 Win Mag | 44.75 | 3 | Recreation |
| Ruger | Hawkeye | Rifle | 30-06 Sprg | 42.75 | 4 | Recreation |
| Ruger | Hawkeye | Rifle | 308 Win | 42.00 | 4 | Recreation |
| Ruger | Hawkeye | Rifle | 7mm Rem Mag | 44.75 | 3 | Recreation |
| Ruger | Hawkeye | Rifle | 7mm-08 Rem | 42.00 | 4 | Recreation |
| Ruger | Hawkeye African | Rifle | 300 Win Mag | 44.88 | 3 | Recreation |
| Ruger | Hawkeye African | Rifle | 338 Win Mag | 44.88 | 3 | Recreation |
| Ruger | Hawkeye African | Rifle | 375 Ruger | 44.88 | 3 | Recreation |
| Ruger | Hawkeye African | Rifle | 375 Ruger | 44.88 | 3 | Recreation |
| Ruger | Hawkeye African | Rifle | 416 Ruger | 44.88 | 3 | Recreation |
| Ruger | Hawkeye FTW Hunter | Rifle | 300 Win Mag | 44.75 | 3 | Recreation |
| Ruger | Hawkeye FTW Hunter | Rifle | 300 Win Mag | 44.75 | 3 | Recreation |
| Ruger | Hawkeye FTW Hunter | Rifle | 308 Win | 42.75 | 4 | Recreation |
| Ruger | Hawkeye FTW Hunter | Rifle | 6.5 Creedmoor | 44.75 | 4 | Recreation |
| Ruger | Hawkeye FTW Hunter | Rifle | 6.5 Creedmoor | 44.75 | 4 | Recreation |
| Ruger | Hawkeye FTW Predator | Rifle | 308 Win | 42.75 | 4 | Recreation |
| Ruger | Hawkeye FTW Predator | Rifle | 6.5 Creedmoor | 44.75 | 4 | Recreation |
| Ruger | Hawkeye Laminate Compact | Rifle | 243 Win | 35.50 | 4 | Recreation |
| Ruger | Hawkeye Laminate Compact | Rifle | 308 Win | 35.50 | 4 | Recreation |
| Ruger | Hawkeye Laminate Compact | Rifle | 7mm-08 Rem | 35.50 | 4 | Recreation |
| Ruger | Hawkeye Long-Range Target | Rifle | 300 Win Mag | 47.00 | 5 | Recreation |
| Ruger | Hawkeye Predator | Rifle | 204 Ruger | 44.00 | 5 | Recreation |
| Ruger | Hawkeye Predator | Rifle | 22-250 Rem | 44.00 | 4 | Recreation |
| Ruger | Hawkeye Predator | Rifle | 223 Rem | 42.00 | 5 | Recreation |
| Ruger | Hawkeye Predator | Rifle | 308 Win | 42.00 | 4 | Recreation |
| Ruger | Hawkeye Predator | Rifle | 6.5 Creedmoor | 44.00 | 4 | Recreation |
| Ruger | Hawkeye Varmint Target | Rifle | 204 Ruger | 46.00 | 5 | Recreation |
| Ruger | Hawkeye Varmint Target | Rifle | 22-250 Rem | 46.00 | 4 | Recreation |
| Ruger | Hawkeye Varmint Target | Rifle | 223 Rem | 46.00 | 5 | Recreation |
| Ruger | Hawkeye Varmint Target | Rifle | 308 Win | 46.00 | 4 | Recreation |
| Ruger | Hawkeye Varmint Target | Rifle | 6.5 Creedmoor | 48.00 | 4 | Recreation |
| Ruger | Mini Thirty | Rifle | 7.62x39 | 37.50 | 20 | Recreation |
| Ruger | Mini Thirty | Rifle | 7.62x39 | 37.50 | 5 | Recreation |
| Ruger | Mini Thirty | Rifle | 7.62x39 | 37.50 | 5 | Recreation |
| Ruger | Mini Thirty Tactical | Rifle | 7.62x39 | 36.75 | 20 | Tactical |
| Ruger | Mini Thirty Tactical | Rifle | 7.62x39 | 36.75 | 20 | Tactical |
| Ruger | Mini-14 | Rifle | 5.56 NATO / 223 Rem | 38.00 | 20 | Recreation |
| Ruger | Mini-14 | Rifle | 5.56 NATO / 223 Rem | 38.00 | 5 | Recreation |
| Ruger | Mini-14 | Rifle | 5.56 NATO / 223 Rem | 38.00 | 5 | Recreation |
| Ruger | Mini-14 | Rifle | 5.56 NATO / 223 Rem | 37.50 | 5 | Recreation |
| Ruger | Mini-14 | Rifle | 5.56 NATO / 223 Rem | 37.50 | 20 | Recreation |
| Ruger | Mini-14 | Rifle | 5.56 NATO / 223 Rem | 37.50 | 5 | Recreation |
| Ruger | Mini-14 Tactical | Rifle | 300 BLK | 36.25 | 20 | Tactical |
| Ruger | Mini-14 Tactical | Rifle | 5.56 NATO / 223 Rem | 36.75 | 20 | Tactical |
| Ruger | Mini-14 Tactical | Rifle | 5.56 NATO / 223 Rem | 36.75 | 5 | Tactical |
| Ruger | Mini-14 Tactical | Rifle | 5.56 NATO / 223 Rem | 36.75 | 20 | Tactical |
| Ruger | Mini-14 Tactical | Rifle | 5.56 NATO / 223 Rem | 37.75 | 20 | Tactical |
| Ruger | No. 1 | Rifle | 450 Bushmaster | 36.50 | 1 | Recreation |
| Ruger | No. 1 | Rifle | 450 Martin | 36.50 | 1 | Recreation |
| Ruger | PC Carbine | Rifle | 9mm | 34.37 | 17 | Recreation |
| Ruger | PC Carbine | Rifle | 9mm | 34.37 | 10 | Recreation |
| Ruger | PC Carbine | Rifle | 9mm | 34.37 | 10 | Recreation |
| Ruger | Ruger 77/17 | Rifle | 17 Hornet | 43.25 | 6 | Recreation |
| Ruger | Ruger 77/17 | Rifle | 17 WSM | 43.25 | 6 | Recreation |
| Ruger | Ruger 77/22 | Rifle | 22 Hornet | 39.25 | 6 | Recreation |
| Ruger | Ruger 77/22 | Rifle | 22 Hornet | 43.25 | 6 | Recreation |
| Ruger | Ruger 77/357 | Rifle | 357 Mag | 38.50 | 5 | Recreation |
| Ruger | Ruger 77/44 with American Walnut Stock | Rifle | 44 Rem Mag | 38.50 | 4 | Recreation |
| Ruger | Ruger 77/44 with Black Synthetic Stock | Rifle | 44 Rem Mag | 38.50 | 4 | Recreation |
| Ruger | Ruger 77/44 with Black Synthetic Stock | Rifle | 44 Rem Mag | 38.50 | 4 | Recreation |
| Ruger | Ruger 77/44 with Camo Stock | Rifle | 44 Rem Mag | 38.50 | 4 | Recreation |
| Ruger | Ruger American Rifle | Rifle | 22-250 Rem | 42.00 | 4 | Recreation |
| Ruger | Ruger American Rifle | Rifle | 223 Rem | 42.00 | 5 | Recreation |
| Ruger | Ruger American Rifle | Rifle | 243 Win | 42.00 | 4 | Recreation |
| Ruger | Ruger American Rifle | Rifle | 243 Win | 42.00 | 4 | Recreation |
| Ruger | Ruger American Rifle | Rifle | 270 Win | 42.50 | 4 | Recreation |
| Ruger | Ruger American Rifle | Rifle | 270 Win | 42.50 | 4 | Recreation |
| Ruger | Ruger American Rifle | Rifle | 30-06 Sprg | 42.50 | 4 | Recreation |
| Ruger | Ruger American Rifle | Rifle | 30-06 Sprg | 42.50 | 4 | Recreation |
| Ruger | Ruger American Rifle | Rifle | 308 Win | 42.00 | 4 | Recreation |
| Ruger | Ruger American Rifle | Rifle | 308 Win | 42.00 | 4 | Recreation |
| Ruger | Ruger American Rifle | Rifle | 7mm-08 Rem | 42.00 | 4 | Recreation |
| Ruger | Ruger American Rifle | Rifle | 7mm-08 Rem | 42.00 | 4 | Recreation |
| Ruger | Ruger American Rifle Compact | Rifle | 243 Win | 36.75 | 4 | Recreation |
| Ruger | Ruger American Rifle Compact | Rifle | 308 Win | 36.75 | 4 | Recreation |
| Ruger | Ruger American Rifle Compact | Rifle | 7mm-08 Rem | 36.75 | 4 | Recreation |
| Ruger | Ruger American Rifle Magnum | Rifle | 300 Win Mag | 44.50 | 3 | Recreation |
| Ruger | Ruger American Rifle Predator | Rifle | 204 Ruger | 42.00 | 10 | Recreation |
| Ruger | Ruger American Rifle Predator | Rifle | 22-250 Rem | 42.00 | 4 | Recreation |
| Ruger | Ruger American Rifle Predator | Rifle | 223 Rem | 42.00 | 10 | Recreation |
| Ruger | Ruger American Rifle Predator | Rifle | 243 Win | 42.00 | 3 | Recreation |
| Ruger | Ruger American Rifle Predator | Rifle | 308 Win | 38.00 | 3 | Recreation |
| Ruger | Ruger American Rifle Predator | Rifle | 308 Win | 38.00 | 4 | Recreation |
| Ruger | Ruger American Rifle Predator | Rifle | 6.5 Creedmoor | 42.00 | 3 | Recreation |
| Ruger | Ruger American Rifle Predator | Rifle | 6.5 Creedmoor | 42.00 | 4 | Recreation |
| Ruger | Ruger American Rifle Predator | Rifle | 6.5 Creedmoor | 42.00 | 4 | Recreation |
| Ruger | Ruger American Rifle Predator | Rifle | 6.5 Grendel | 42.00 | 10 | Recreation |
| Ruger | Ruger American Rifle Predator | Rifle | 6mm Creedmoor | 42.00 | 3 | Recreation |
| Ruger | Ruger American Rifle Predator with Riflescope | Rifle | 204 Ruger | 42.00 | 10 | Recreation |
| Ruger | Ruger American Rifle Predator with Riflescope | Rifle | 223 Rem | 42.00 | 10 | Recreation |
| Ruger | Ruger American Rifle Predator with Riflescope | Rifle | 308 Win | 38.00 | 3 | Recreation |
| Ruger | Ruger American Rifle Predator with Riflescope | Rifle | 6.5 Creedmoor | 42.00 | 3 | Recreation |
| Ruger | Ruger American Rifle Predator with Riflescope | Rifle | 6.5 Creedmoor | 42.00 | 4 | Recreation |
| Ruger | Ruger American Rifle Ranch | Rifle | 300 BLK | 36.00 | 10 | Recreation |
| Ruger | Ruger American Rifle Ranch | Rifle | 300 BLK | 36.00 | 5 | Recreation |
| Ruger | Ruger American Rifle Ranch | Rifle | 450 Bushmaster | 36.00 | 3 | Recreation |
| Ruger | Ruger American Rifle Ranch | Rifle | 5.56 NATO / 223 Rem | 36.00 | 10 | Recreation |
| Ruger | Ruger American Rifle Ranch | Rifle | 5.56 NATO / 223 Rem | 36.00 | 5 | Recreation |
| Ruger | Ruger American Rifle Ranch | Rifle | 7.62x39 | 36.00 | 5 | Recreation |
| Ruger | Ruger American Rifle with Camo Stock | Rifle | 243 Win | 42.00 | 3 | Recreation |
| Ruger | Ruger American Rifle with Camo Stock | Rifle | 300 Win Mag | 44.00 | 3 | Recreation |
| Ruger | Ruger American Rifle with Camo Stock | Rifle | 30-06 Sprg | 42.00 | 4 | Recreation |
| Ruger | Ruger American Rifle with Camo Stock | Rifle | 308 Win | 42.00 | 3 | Recreation |
| Ruger | Ruger American Rifle with Camo Stock | Rifle | 450 Bushmaster | 42.00 | 3 | Recreation |
| Ruger | Ruger American Rifle with Camo Stock | Rifle | 6.5 Creedmoor | 42.00 | 3 | Recreation |
| Ruger | Ruger American Rifle with Camo Stock | Rifle | 7mm-08 Rem | 42.00 | 3 | Recreation |
| Ruger | Ruger American Rifle with Riflescope | Rifle | 223 Rem | 42.00 | 5 | Recreation |
| Ruger | Ruger American Rifle with Riflescope | Rifle | 243 Win | 42.00 | 4 | Recreation |
| Ruger | Ruger American Rifle with Riflescope | Rifle | 270 Win | 42.50 | 4 | Recreation |
| Ruger | Ruger American Rifle with Riflescope | Rifle | 30-06 Sprg | 42.50 | 4 | Recreation |
| Ruger | Ruger American Rifle with Riflescope | Rifle | 308 Win | 42.00 | 4 | Recreation |
| Ruger | Ruger American Rimfire | Rifle | 17 HMR | 41.00 | 9 | Recreation |
| Ruger | Ruger American Rimfire | Rifle | 17 HMR | 37.00 | 9 | Recreation |
| Ruger | Ruger American Rimfire | Rifle | 22 LR | 41.00 | 10 | Recreation |
| Ruger | Ruger American Rimfire | Rifle | 22 LR | 37.00 | 10 | Recreation |
| Ruger | Ruger American Rimfire | Rifle | 22 WMR | 41.00 | 9 | Recreation |
| Ruger | Ruger American Rimfire | Rifle | 22 WMR | 37.00 | 9 | Recreation |
| Ruger | Ruger American Rimfire Compact | Rifle | 17 HMR | 35.75 | 9 | Recreation |
| Ruger | Ruger American Rimfire Compact | Rifle | 22 LR | 35.75 | 10 | Recreation |
| Ruger | Ruger American Rimfire Compact | Rifle | 22 LR | 35.75 | 10 | Recreation |
| Ruger | Ruger American Rimfire Compact | Rifle | 22 WMR | 35.75 | 9 | Recreation |
| Ruger | Ruger American Rimfire Stainless | Rifle | 17 HMR | 37.00 | 9 | Recreation |
| Ruger | Ruger American Rimfire Stainless | Rifle | 22 LR | 37.00 | 10 | Recreation |
| Ruger | Ruger American Rimfire Stainless | Rifle | 22 WMR | 37.00 | 9 | Recreation |
| Ruger | Ruger American Rimfire Target | Rifle | 17 HMR | 37.00 | 9 | Recreation |
| Ruger | Ruger American Rimfire Target | Rifle | 22 LR | 37.00 | 10 | Recreation |
| Ruger | Ruger American Rimfire Target | Rifle | 22 WMR | 37.00 | 9 | Recreation |
| Ruger | Ruger American Rimfire with Thumbhole Stock | Rifle | 22 LR | 37.00 | 10 | Recreation |
| Ruger | Ruger American Rimfire with Wood Stock | Rifle | 22 LR | 41.00 | 10 | Recreation |
| Ruger | Ruger Guide Gun | Rifle | 300 Win Mag | 42.50 | 3 | Recreation |
| Ruger | Ruger Guide Gun | Rifle | 30-06 Sprg | 42.50 | 4 | Recreation |
| Ruger | Ruger Guide Gun | Rifle | 338 Win Mag | 42.50 | 3 | Recreation |
| Ruger | Ruger Guide Gun | Rifle | 375 Ruger | 42.50 | 3 | Recreation |
| Ruger | Ruger Guide Gun | Rifle | 375 Ruger | 42.50 | 3 | Recreation |
| Ruger | Ruger Guide Gun | Rifle | 416 Ruger | 42.50 | 3 | Recreation |
| Ruger | Ruger Precision Rifle | Rifle | 308 Win | 42.75 | 10 | Recreation |
| Ruger | Ruger Precision Rifle | Rifle | 6.5 Creedmoor | 46.75 | 10 | Recreation |
| Ruger | Ruger Precision Rifle | Rifle | 6mm Creedmoor | 46.75 | 10 | Recreation |
| Ruger | Ruger Precision Rimfire | Rifle | 22 LR | 38.63 | 15 | Recreation |
| Ruger | Ruger Precision Rimfire | Rifle | 22 LR | 38.63 | 10 | Recreation |
| Ruger | Ruger Scout Rifle | Rifle | 308 Win | 38.50 | 10 | Recreation |
| Ruger | Ruger Scout Rifle | Rifle | 308 Win | 41.00 | 10 | Recreation |
| Ruger | Ruger Scout Rifle | Rifle | 308 Win | 41.00 | 10 | Recreation |
| Ruger | Ruger Scout Rifle | Rifle | 308 Win | 38.50 | 10 | Recreation |
| Ruger | Ruger Scout Rifle | Rifle | 308 Win | 38.50 | 10 | Recreation |
| Ruger | Ruger Scout Rifle | Rifle | 450 Bushmaster | 38.50 | 4 | Recreation |
| Ruger | Ruger Scout Rifle | Rifle | 5.56 NATO / 223 Rem | 38.50 | 10 | Recreation |
| Ruger | Ruger Scout Rifle | Rifle | 5.56 NATO / 223 Rem | 38.50 | 10 | Recreation |
| Ruger | SR-556 Takedown | Rifle | 5.56 NATO / 223 Rem | 36.00 | 30 | Tactical |
| Ruger | SR-762 | Rifle | 308 Win / 7.62 NATO | 38.00 | 20 | Tactical |
| Savage | 64 | Rifle | 22LR | 40 | 10 | Recreation |
| Savage | 10 BA Stealth | Rifle | Multiple | Adjustable | 10 | Tactical |
| Savage | 10/110 FCP HS PRECISION | Rifle | Multiple | 44-44.9 | 4 | Tactical |
| Savage | 10/110 FCP HS PRECISION | Rifle | Multiple | 46.9 | 5 | Tactical |
| Savage | 10/110 TROPHY HUNTER XP | Rifle | Mutilple | 41.5-42.5 | 4 | Recreation |
| Savage | 11 TROPHY HUNTER XP | Rifle | Mutilple | 42.6-43.5 | 4 | Recreation |
| Savage | 11 TROPHY HUNTER XP | Rifle | Mutilple | 44.6" | 2 | Recreation |
| Savage | 11 TROPHY HUNTER XP | Rifle | Mutilple | 45.5" | 4 | Recreation |
| Savage | 11 TROPHY PREDATOR HUNTER XP | Rifle | Multiple | 42.6" | 4 | Recreation |
| Savage | 11/111 BTH | Rifle | Multiple | 42.5-43.4 | 4 | Recreation |
| Savage | 11/111 Lady Hunter | Rifle | Multiple | 39.5-40.75 | 4 | Recreation |
| Savage | 11/111 Lightweight Hunter | Rifle | Multiple | 40.25-41.5 | 4 | Recreation |
| Savage | 110 BA Stealth | Rifle | Multiple | Adjustable | 5 | Tactical |
| Savage | 110 BA Stealth Evolution | Rifle | Multiple | Adjustable | 10 | Tactical |
| Savage | 110 Bear Hunter | Rifle | Multiple | 42.5-44.13 | 2-4 | Recreation |
| Savage | 110 Brush Hunter | Rifle | 338 WIN. MAG, 375 RUGER | 40 - 41" | 3 | Recreation |
| Savage | 110 Hog Hunter | Rifle | 223 REM, 308 WIN, 338 FEDERAL | 39 - 40" | 4 | Recreation |
| Savage | 110 Hunter | Rifle | Multiple | 41-44.5 | 3-4 | Recreation |
| Savage | 110 Lightweight Storm | Rifle | Multiple | 39.25 - 40.25" | 4 | Recreation |
| Savage | 110 Long Range Hunter | Rifle | 338 Lapua | 48.5-49.5 | 5 | Recreation |
| Savage | 110 Long Range Hunter | Rifle | Multiple | 45.5-46.25 | 2-4 | Recreation |
| Savage | 110 Long Range Hunter | Rifle | Multple | 45.5-46.25 | 3 | Recreation |
| Savage | 110 Predator | Rifle | Multiple | 41-44 | 4 | Recreation |
| Savage | 110 Scout | Rifle | 308 WIN, 223 REM., 228 FEDERAL, 450 BUSHMASTER | 37.5 - 38.5" | 10 | Tactical |
| Savage | 110 Storm | Rifle | Multiple | 41-44.5 | 2-4 | Recreation |
| Savage | 110 Tactical/ Tactical Desert | Rifle | 308 Win, 6/6.5 CREEDMOOR | 43.25-48.25 | 10 | Tactical |
| Savage | 110 TROPHY HUNTER XP | Rifle | 7MM REM MAG, 300 WIN MAG | 44.5" | 3 | Recreation |
| Savage | 110 Varmint | Rifle | 223 REM, 22-250 REM, 204 RUGER | 45-46 | 4 | Recreation |
| Savage | 110 Wolverine | Rifle | 450 BUSHMASTER | 37.5 - 38.5" | 4 | Recreation |
| Savage | 112 MAGNUM TARGET | Rifle | 338 LAPUA | 49.8" | 1 | Recreation |
| Savage | 12 BENCHREST | Rifle | 6 NORMA BR, 6.5 X 284 NORMA, 308 WIN. | 49" | 1 | Recreation |
| Savage | 12 BTCSS | Rifle | 223 REM, 204 RUGER, 22-250 REM | 46.25" | 4 | Recreation |
| Savage | 12 BVSS | Rifle | 223 REM., 22-250 REM., 308 WIN. | 46.5" | 4 | Recreation |
| Savage | 12 F CLASS | Rifle | 6 NORMA BR, 6.5 X 284 NORMA | 50" | 1 | Recreation |
| Savage | 12 F/TR | Rifle | 223 REM., 308 WIN. | 50" | 1 | Recreation |
| Savage | 12 LONG RANGE PRECISION | Rifle | 260 REM., 243 WIN., 6.5 CREEDMOOR | 46" | 4 | Recreation |
| Savage | 12 LONG RANGE PRECISION VARMINTER | Rifle | 223 REM (7” TWIST), 223 REM (9” TWIST), 204 RUGER, 22-250 (9” TWIST), 22-250 (12” TWIST), 6 NORMA BR | 46.25" | 1 | Recreation |
| Savage | 12 PALMA® | Rifle | 308 WIN | 49.75" | 4 | Recreation |
| Savage | 12 VARMINT LOW PROFILE | Rifle | 223 REM (7” TWIST), 223 REM (9” TWIST), 204 RUGER, 22-250 (9” TWIST), 22-250 (12” TWIST), 243 WIN, 308 WIN, 300 WSM | 46.25" | 2/4 | Recreation |
| Savage | 14/114 American Classic | Rifle | 7MM REM. MAG, 300 WIN. MAG | 44.75 | 3 | Recreation |
| Savage | 14/114 American Classic | Rifle | Multiple | 42-42.75 | 4 | Recreation |
| Savage | 16/116 TROPHY HUNTER XP | Rifle | 270 WSM, 300 WSM | 44.6" | 2 | Recreation |
| Savage | 16/116 TROPHY HUNTER XP | Rifle | 7MM REM MAG, 300 WIN MAG, 338 WIN MAG | 45.5" | 3 | Recreation |
| Savage | 16/116 TROPHY HUNTER XP | Rifle | Multiple | 42.6-43.5" | 4 | Recreation |
| Savage | 25 LIGHTWEIGHT VARMINTER / T | Rifle | 17 HORNET, 22 HORNET, 222 REM., 223 REM., 204 RUGER | 43.75" | 4 | Recreation |
| Savage | 25 WALKING VARMINTER | Rifle | 17 HORNET, 22 HORNET, 222 REM., 223 REM., 204 RUGER | 42.4" | 4 | Recreation |
| Savage | 93 BRJ | Rifle | 22 WMR | 40" | 5 | Recreation |
| Savage | 93 BSEV | Rifle | 22 WMR | 40" | 5 | Recreation |
| Savage | 93 BTVS | Rifle | 22 WMR | 40" | 5 | Recreation |
| Savage | 93 F/FV | Rifle | 22 WMR | 39.5-39.75" | 5 | Recreation |
| Savage | 93 FV-SR | Rifle | 22 WMR | 35.25" | 5 | Recreation |
| Savage | 93 FV-SR LANDRY | Rifle | 22 WMR | 35.25" | 5 | Recreation |
| Savage | 93 FVSS | Rifle | 22 WMR | 39.5-39.75 | 5 | Recreation |
| Savage | 93 FVSS XP | Rifle | 22 WMR | 39.75" | 5 | Recreation |
| Savage | 93 FXP | Rifle | 22 WMR | 39.5" | 5 | Recreation |
| Savage | 93 G | Rifle | 22 WMR | 39.5" | 5 | Recreation |
| Savage | 93 XP CAMO | Rifle | 22 WMR | 39.5" | 5 | Recreation |
| Savage | 93R17 (F, FV, FV-SR, FVSS, GV, BVSS, BTV, BTVS, BRJ, XP, TRR-SR) | Rifle | 17 HMR | 35.35-40" | 5 | Recreation |
| Savage | A17 | Rifle | 17 HMR, 22 WMR | 41.5" | 10 | Recreation |
| Savage | A22 | Rifle | 22 LR |  | 10 | Recreation |
| Savage | Axis (Compact, SR, XP) | Rifle | 223 REM., 22-250 REM., 243 WIN., 7MM-08 REM., 308 WIN., 25-06 REM., 270 WIN., 30-06 SPRG, 6.5 CREEDMOOR | 40-42.6" | 4 | Recreation |
| Savage | Axis II (XP, Compact) | Rifle | Multiple | 40-43.875" | 4 | Recreation |
| Savage | B.MAG | Rifle | 17 WSM | 40.5 | 8 | Recreation |
| Savage | B17(F, G) | Rifle | 17 HMR, 22 WMR, 22 LR | 39" | 10 | Recreation |
| Savage | BSS (FV, FVSS, F, Magnum FV-SR) | Rifle | 17 HMR, 22 WMR, 22 LR | 35.25-39" | 10 | Recreation |
| Savage | ENGAGE HUNTER XP | Rifle | Multiple | 42.6 - 45.6" | 2-4 | Recreation |
| Savage | Mark I FVT | Rifle | 22 SHORT, 22 LONG, 22LR | 39.5" | 1 | Recreation |
| Savage | MARK I G | Rifle | 22 SHORT, 22 LONG, 22LR | 39.5" | 1 | Recreation |
| Savage | MARK I GY COMPACT | Rifle | 22 SHORT, 22 LONG, 22LR | 37" | 1 | Recreation |
| Savage | MARK II (F, Camo, G) | Rifle | 22LR | 39.5" | 10 | Recreation |
| Savage | MARK II (TR/TRR-SR) | Rifle | 22LR | 40" | 5 | Recreation |
| Savage | MARK II BSEV | Rifle | 22LR | 40" | 5 | Recreation |
| Savage | MARK II BTV/BTVS | Rifle | 22LR | 40" | 5 | Recreation |
| Savage | MARK II BV | Rifle | 22LR | 39.75" | 5 | Recreation |
| Savage | MARK II FV/FVT | Rifle | 22LR, 17 HM2 | 39.75" | 5 | Recreation |
| Savage | MARK II FV-SR LANDRY | Rifle | 22LR | 35.25" | 5 | Recreation |
| Savage | MARK II FVXP | Rifle | 22LR | 39.5" | 5 | Recreation |
| Savage | MARK II FXP | Rifle | 22LR | 39.5" | 10 | Recreation |
| Savage | MSR 10 Hunter | Rifle | Multiple | 35-41 | 20 | Tactical |
| Savage | MSR 10 Long Range | Rifle | Multiple | 40.63-45.13 | 10 | Tactical |
| Savage | MSR 15 Long Recon | Rifle | 223 REM/ 5.56 MM | 33.5 - 36.75" | 30 | Tactical |
| Savage | MSR 15 Patrol | Rifle | 223 REM/ 5.56 MM | 32.5 - 35.75" | 30 | Tactical |
| Savage | MSR 15 RECON LRP | Rifle | 224 VALKYRIE, 22 NOSLER, 6.8 SPC, 223 REM | 35.25 - 38.5 | 25 | Tactical |
| Savage | MSR 15 Valkyrie | Rifle | 224 VALKYRIE | 35.5 - 39.0 | 25 | Tactical |
| Savage | Rascal | Rifle | 22 SHORT, 22 LONG, 22LR | 31.5" | 1 | Recreation |
| Sig Sauer | SIG MCX Rattler PSB | Rifle | 300 BLK | 19.30 | 30 | Tactical |
| Sig Sauer | SIG MCX Rattler SBR | Rifle | 300 BLK | 23.50 | 30 | Tactical |
| Sig Sauer | SIG MCX Virtus Patrol 16" | Rifle | 5.56 NATO / 300 BLK | 35.50 | 30 | Tactical |
| Sig Sauer | SIG MCX Virtus PSB 11" | Rifle | 5.56 NATO | 31.00 | 30 | Tactical |
| Sig Sauer | SIG MCX Virtus PSB 9" | Rifle | 300 BLK | 28.50 | 30 | Tactical |
| Sig Sauer | SIG MCX Virtus SBR 11" | Rifle | 5.56 NATO | 31.00 | 30 | Tactical |
| Sig Sauer | SIG MCX Virtus SBR 9" | Rifle | 300 BLK | 28.50 | 30 | Tactical |
| Sig Sauer | SIG MPX Carbine | Rifle | 9mm | 33.00 | 30 | Tactical |
| Sig Sauer | SIG MPX K PSB | Rifle | 9mm | 22.50 | 30 | Tactical |
| Sig Sauer | SIG MPX PSB | Rifle | 9mm | 25.75 | 30 | Tactical |
| Sig Sauer | SIG516 Patrol | Rifle | 5.56 NATO | 36.50 | 30 | Tactical |
| Sig Sauer | SIG716G2 DMR | Rifle | 7.62 NATO | 37.00 | 30 | Tactical |
| Sig Sauer | SIG716G2 Patrol | Rifle | 7.62 NATO | 37.00 | 30 | Tactical |
| Sig Sauer | SIGM400 Elite | Rifle | 5.56 NATO | 36.50 | 30 | Tactical |
| Sig Sauer | SIGM400 Elite PSB | Rifle | 5.56 NATO | 30.75 | 30 | Tactical |
| Sig Sauer | SIGM400 Elite Titanium +R | Rifle | 5.56 NATO | 36.50 | 30 | Tactical |
| Sig Sauer | SIGM400 Vanish | Rifle | 5.56 NATO | 36.50 | 30 | Tactical |
| Smith & Wesson | M&P 10 | Rifle | 308 WIN/7.62mm x 51 Nato | 18 | 20 PMAG | Tactical |
| Smith & Wesson | M&P 15 | Rifle | 300 Whisper/300 AAC Blackout | 16 | 30 PMAG | Tactical |
| Smith & Wesson | M&P 15 | Rifle | 5.56mm NATO/223 REM | 16 | 30 PMAG | Tactical |
| Smith & Wesson | M&P 15 Sport II | Rifle | 5.56mm NATO/223 REM | 16 | 30 PMAG | Tactical |
| Smith & Wesson | M&P 15-22 Sport | Rifle | 22LR | 16.5 | 25 | Tactical |
| Smith & Wesson | Performance Center Rifles | Rifle | 22LR | 18 | 10 | Tactical |
| Smith & Wesson | Performance Center Rifles | Rifle | 5.56mm NATO/223REM | 18 | 30 | Tactical |
| Smith & Wesson | Performance Center Rifles | Rifle | 6.5 Creedmoor | 18 | 10 | Tactical |
| Smith & Wesson | Performance Center T/C LRR Rifles | Rifle | 243 win | 26 | 10 | Tactical |
| Smith & Wesson | Performance Center T/C LRR Rifles | Rifle | 308 WIN | 20 | 10 | Tactical |
| Smith & Wesson | Performance Center T/C LRR Rifles | Rifle | 6.5 Creedmoor | 24 | 10 | Tactical |
| Smith & Wesson | Performance Center T/CR 22 Rifles | Rifle | 22LR | 20 | 10 | Recreation |
| Springfield | Loaded M1A Models | Rifle | 7.62 NATO / 6.5 Creedmoor | 46.25 | 10 | Recreation |
| Springfield | National Match M1A | Rifle | 7.62 NATO | 44.33 | 10 | Recreation |
| Springfield | SAINT AR-15 | Rifle | 5.56 NATO | 36.25 | 30 | Tactical |
| Springfield | SAINT Edge | Rifle | 5.56 NATO | 35.75 | 30 | Tactical |
| Springfield | SAINT Edge SBR | Rifle | 5.56 NATO | 30.75 | 30 | Tactical |
| Springfield | SAINT SBR | Rifle | 5.56 NATO | 30.75 | 30 | Tactical |
| Springfield | Scout Squad M1A | Rifle | 7.62 NATO | 40.33 | 10 | Recreation |
| Springfield | SOCOM 16 M1A | Rifle | 7.62 NATO | 38.50 | 10 | Recreation |
| Springfield | Standard M1A | Rifle | 7.62 NATO | 44.33 | 10 | Recreation |
| Springfield | Super Match M1A | Rifle | 7.62 NATO | 44.33 | 10 | Recreation |
| Steyr | AUG A3 M1 | Rifle | 223 REM / 5.56 NATO | 28.15 | 42 | Tactical |
| Steyr | CL II | Rifle | Multiple | 46.90 | 4 | Recreation |
| Steyr | CL II SX | Rifle | Multiple | 46.30 | 4 | Recreation |
| Steyr | HS .50 (single shot) | Rifle | 50 BMG | 57.30 | 1 | Recreation |
| Steyr | HS .50-M1 | Rifle | 50 BMG | 59.40 | 5 | Recreation |
| Steyr | PRO THB | Rifle | 308 Win / 6.5 Creedmoor | 45.20 | 4 | Recreation |
| Steyr | Scout | Rifle | Multiple | 42.50 | 5 | Recreation |
| Steyr | SM12 | Rifle | Multiple | 46.90 | 4 | Recreation |
| Steyr | SM12 SX | Rifle | Multiple | 46.30 | 4 | Recreation |
| Steyr | SSG 04 (includes SSG 04 A1) | Rifle | 243 Win / 308 Win | 46.30 | 10 | Recreation |
| Steyr | SSG 04 (includes SSG 04 A1) | Rifle | 300 Win Mag | 46.30 | 8 | Recreation |
| Steyr | SSG 08 | Rifle | 243 Win / 308 Win | 47.00 | 10 | Recreation |
| Steyr | SSG 08 | Rifle | 300 Win Mag | 47.00 | 8 | Recreation |
| Steyr | SSG 08 | Rifle | 338 Lapua Mag | 49.00 | 6 | Recreation |
| Steyr | SSG 08 A1 | Rifle | 308 Win | 46.10 | 10 | Recreation |
| Steyr | SSG 08 A1 | Rifle | 338 Lapua Mag | 49.10 | 6 | Recreation |
| Steyr | Zephyr II | Rifle | 17 HMR / 22 LR / 22 WMR | 39.20 | 5 | Recreation |
| Unique ARs | AR-15 | Rifle | 223 Wylde |  | 30 | Tactical |
| Winchester | Model 1866 Short Rifle | Rifle | 38 Spcl | 20 | 11 | Recreation |
| Winchester | Model 1866 Short Rifle | Rifle | 44-40 Win | 20 | 10 | Recreation |
| Winchester | Model 1866 Short Rifle | Rifle | 45 Colt | 20 | 10 | Recreation |
| Winchester | Model 1873 Carbine | Rifle | 357 Mag/38 Spl | 20 | 11 | Recreation |
| Winchester | Model 1873 Carbine | Rifle | 44-40 Win | 20 | 10 | Recreation |
| Winchester | Model 1873 Carbine | Rifle | 45 Colt | 20 | 10 | Recreation |
| Winchester | Model 1873 Short Rifle | Rifle | 357 Mag/38 Spl | 20 | 11 | Recreation |
| Winchester | Model 1873 Short Rifle | Rifle | 44-40 Win | 20 | 10 | Recreation |
| Winchester | Model 1873 Short Rifle | Rifle | 45 Colt | 20 | 10 | Recreation |
| Winchester | Model 1873 Short Rifle Color Case Hardened | Rifle | 357 Mag/38 Spl | 20 | 11 | Recreation |
| Winchester | Model 1873 Short Rifle Color Case Hardened | Rifle | 44-40 Win | 20 | 10 | Recreation |
| Winchester | Model 1873 Short Rifle Color Case Hardened | Rifle | 45 Colt | 20 | 10 | Recreation |
| Winchester | Model 1873 Sporter Octagon Color Case Hardened | Rifle | 357 Mag/38 Spl | 24 | 14 | Recreation |
| Winchester | Model 1873 Sporter Octagon Color Case Hardened | Rifle | 44-40 Win | 24 | 13 | Recreation |
| Winchester | Model 1873 Sporter Octagon Color Case Hardened | Rifle | 45 Colt | 24 | 13 | Recreation |
| Winchester | Model 1873 Sporter Octagon Pistol Grip | Rifle | 357 Mag/38 Spl | 24 | 14 | Recreation |
| Winchester | Model 1873 Sporter Octagon Pistol Grip | Rifle | 44-40 Win | 24 | 13 | Recreation |
| Winchester | Model 1873 Sporter Octagon Pistol Grip | Rifle | 45 Colt | 24 | 13 | Recreation |
| Winchester | Model 1873 Sporter Octagon Pistol Grip Color Case Hardened | Rifle | 357 Mag/38 Spl | 24 | 14 | Recreation |
| Winchester | Model 1873 Sporter Octagon Pistol Grip Color Case Hardened | Rifle | 44-40 Win | 24 | 13 | Recreation |
| Winchester | Model 1873 Sporter Octagon Pistol Grip Color Case Hardened | Rifle | 45 Colt | 24 | 13 | Recreation |
| Winchester | Model 1885 Low Wall Hunter Rimfire | Rifle | 17 HMR | 24 | N/A (?1) | Recreation |
| Winchester | Model 1885 Low Wall Hunter Rimfire | Rifle | 17 WSM | 24 | N/A (?1) | Recreation |
| Winchester | Model 1885 Low Wall Hunter Rimfire | Rifle | 22 LR | 24 | N/A (?1) | Recreation |
| Winchester | Model 1885 Low Wall Hunter Rimfire | Rifle | 22 WMR | 24 | N/A (?1) | Recreation |
| Winchester | Model 1886 Deluxe Rifle | Rifle | 45-70 Govt | 24 | 8 | Recreation |
| Winchester | Model 1886 Short Rifle | Rifle | 45-70 Govt | 24 | 8 | Recreation |
| Winchester | Model 1892 Carbine | Rifle | 357 Mag | 20 | 10 | Recreation |
| Winchester | Model 1892 Carbine | Rifle | 44 Rem Mag | 20 | 10 | Recreation |
| Winchester | Model 1892 Carbine | Rifle | 44-40 | 20 | 10 | Recreation |
| Winchester | Model 1892 Carbine | Rifle | 45 Colt | 20 | 10 | Recreation |
| Winchester | Model 1892 Short Rifle | Rifle | 357 Mag | 20 | 10 | Recreation |
| Winchester | Model 1892 Short Rifle | Rifle | 44 Rem Mag | 20 | 10 | Recreation |
| Winchester | Model 1892 Short Rifle | Rifle | 45 Colt | 20 | 10 | Recreation |
| Winchester | Model 70 Alaskan | Rifle | 300 Win Mag | 25 | 3 | Recreation |
| Winchester | Model 70 Alaskan | Rifle | 30-06 Sprg | 25 | 5 | Recreation |
| Winchester | Model 70 Alaskan | Rifle | 338 Win Mag | 25 | 3 | Recreation |
| Winchester | Model 70 Alaskan | Rifle | 375 H&H Mag | 25 | 3 | Recreation |
| Winchester | Model 70 Coyote Light Suppressor Ready | Rifle | 22-250 Rem | 24 | 5 | Recreation |
| Winchester | Model 70 Coyote Light Suppressor Ready | Rifle | 243 Win | 24 | 5 | Recreation |
| Winchester | Model 70 Coyote Light Suppressor Ready | Rifle | 270 WSM | 24 | 3 | Recreation |
| Winchester | Model 70 Coyote Light Suppressor Ready | Rifle | 300 WSM | 24 | 3 | Recreation |
| Winchester | Model 70 Coyote Light Suppressor Ready | Rifle | 308 Win | 24 | 5 | Recreation |
| Winchester | Model 70 Coyote Light Suppressor Ready | Rifle | 325 WSM | 24 | 3 | Recreation |
| Winchester | Model 70 Extreme Weather SS | Rifle | 243 Win | 22 | 5 | Recreation |
| Winchester | Model 70 Extreme Weather SS | Rifle | 25-06 Rem | 22 | 5 | Recreation |
| Winchester | Model 70 Extreme Weather SS | Rifle | 264 Win Mag | 26 | 3 | Recreation |
| Winchester | Model 70 Extreme Weather SS | Rifle | 270 Win | 22 | 5 | Recreation |
| Winchester | Model 70 Extreme Weather SS | Rifle | 270 WSM | 24 | 3 | Recreation |
| Winchester | Model 70 Extreme Weather SS | Rifle | 300 Win Mag | 26 | 3 | Recreation |
| Winchester | Model 70 Extreme Weather SS | Rifle | 300 WSM | 24 | 3 | Recreation |
| Winchester | Model 70 Extreme Weather SS | Rifle | 30-06 Sprg | 22 | 5 | Recreation |
| Winchester | Model 70 Extreme Weather SS | Rifle | 308 Win | 22 | 5 | Recreation |
| Winchester | Model 70 Extreme Weather SS | Rifle | 325 WSM | 24 | 3 | Recreation |
| Winchester | Model 70 Extreme Weather SS | Rifle | 338 Win Mag | 26 | 3 | Recreation |
| Winchester | Model 70 Extreme Weather SS | Rifle | 7mm Rem Mag | 26 | 3 | Recreation |
| Winchester | Model 70 Extreme Weather SS | Rifle | 7mm-08 Rem | 22 | 5 | Recreation |
| Winchester | Model 70 Featherweight | Rifle | 22-250 Rem | 22 | 5 | Recreation |
| Winchester | Model 70 Featherweight | Rifle | 243 Win | 22 | 5 | Recreation |
| Winchester | Model 70 Featherweight | Rifle | 25-06 Rem | 22 | 5 | Recreation |
| Winchester | Model 70 Featherweight | Rifle | 264 Win Mag | 24 | 3 | Recreation |
| Winchester | Model 70 Featherweight | Rifle | 270 Win | 22 | 5 | Recreation |
| Winchester | Model 70 Featherweight | Rifle | 270 WSM | 24 | 3 | Recreation |
| Winchester | Model 70 Featherweight | Rifle | 300 Win Mag | 24 | 3 | Recreation |
| Winchester | Model 70 Featherweight | Rifle | 300 WSM | 24 | 3 | Recreation |
| Winchester | Model 70 Featherweight | Rifle | 30-06 Sprg | 22 | 5 | Recreation |
| Winchester | Model 70 Featherweight | Rifle | 308 Win | 22 | 5 | Recreation |
| Winchester | Model 70 Featherweight | Rifle | 325 WSM | 24 | 3 | Recreation |
| Winchester | Model 70 Featherweight | Rifle | 7mm-08 Rem | 22 | 5 | Recreation |
| Winchester | Model 70 Featherweight Compact | Rifle | 22-250 Rem | 20 | 5 | Recreation |
| Winchester | Model 70 Featherweight Compact | Rifle | 243 Win | 20 | 5 | Recreation |
| Winchester | Model 70 Featherweight Compact | Rifle | 308 Win | 20 | 5 | Recreation |
| Winchester | Model 70 Featherweight Compact | Rifle | 7mm-08 Rem | 20 | 5 | Recreation |
| Winchester | Model 70 Safari Express | Rifle | 375 H&H Mag | 24 | 3 | Recreation |
| Winchester | Model 70 Safari Express | Rifle | 416 Rem Mag | 24 | 3 | Recreation |
| Winchester | Model 70 Safari Express | Rifle | 458 Win Mag | 24 | 3 | Recreation |
| Winchester | Model 70 Sporter | Rifle | 25-06 Rem | 24 | 5 | Recreation |
| Winchester | Model 70 Sporter | Rifle | 264 Win Mag | 26 | 3 | Recreation |
| Winchester | Model 70 Sporter | Rifle | 270 Win | 24 | 5 | Recreation |
| Winchester | Model 70 Sporter | Rifle | 270 WSM | 24 | 3 | Recreation |
| Winchester | Model 70 Sporter | Rifle | 300 Win Mag | 26 | 3 | Recreation |
| Winchester | Model 70 Sporter | Rifle | 300 WSM | 24 | 3 | Recreation |
| Winchester | Model 70 Sporter | Rifle | 30-06 Sprg | 24 | 5 | Recreation |
| Winchester | Model 70 Sporter | Rifle | 325 WSM | 24 | 3 | Recreation |
| Winchester | Model 70 Sporter | Rifle | 338 Win Mag | 26 | 3 | Recreation |
| Winchester | Model 70 Sporter | Rifle | 7mm Rem Mag | 26 | 3 | Recreation |
| Winchester | Model 70 Super Grade | Rifle | 270 Win | 24 | 5 | Recreation |
| Winchester | Model 70 Super Grade | Rifle | 270 WSM | 24 | 3 | Recreation |
| Winchester | Model 70 Super Grade | Rifle | 300 Win Mag | 26 | 3 | Recreation |
| Winchester | Model 70 Super Grade | Rifle | 300 WSM | 24 | 3 | Recreation |
| Winchester | Model 70 Super Grade | Rifle | 30-06 Sprg | 24 | 5 | Recreation |
| Winchester | Model 70 Super Grade | Rifle | 338 Win Mag | 26 | 3 | Recreation |
| Winchester | Model 70 Super Grade | Rifle | 7mm Rem Mag | 26 | 3 | Recreation |
| Winchester | Model 94 Carbine | Rifle | 30-30 Win | 20 | 7 | Recreation |
| Winchester | Model 94 Carbine | Rifle | 38-55 Win | 20 | 7 | Recreation |
| Winchester | Model 94 Short Rifle | Rifle | 30-30 Win | 20 | 7 | Recreation |
| Winchester | Model 94 Short Rifle | Rifle | 38-55 Win | 20 | 7 | Recreation |
| Winchester | Model 94 Short Rifle | Rifle | 450 Marlin | 20 | 7 | Recreation |
| Winchester | Model 94 Sporter | Rifle | 30-30 Win | 24 | 8 | Recreation |
| Winchester | Model 94 Sporter | Rifle | 38-55 Win | 24 | 8 | Recreation |
| Winchester | Model 94 Trails End Takedown | Rifle | 30-30 Win | 20 | 6 | Recreation |
| Winchester | Model 94 Trails End Takedown | Rifle | 38-55 Win | 20 | 6 | Recreation |
| Winchester | Model 94 Trails End Takedown | Rifle | 450 Marline | 20 | 6 | Recreation |
| Winchester | XPC | Rifle | 243 Win | 24 | 10 | Recreation |
| Winchester | XPC | Rifle | 308 Win | 20 | 10 | Recreation |
| Winchester | XPC | Rifle | 6.5 Creedmoor | 24 | 10 | Recreation |
| Winchester | XPR | Rifle | 243 Win | 22 | 3 | Recreation |
| Winchester | XPR | Rifle | 270 Win | 24 | 3 | Recreation |
| Winchester | XPR | Rifle | 270 WSM | 24 | 3 | Recreation |
| Winchester | XPR | Rifle | 300 Win Mag | 26 | 3 | Recreation |
| Winchester | XPR | Rifle | 300 WSM | 24 | 3 | Recreation |
| Winchester | XPR | Rifle | 30-06 Sprg | 24 | 3 | Recreation |
| Winchester | XPR | Rifle | 308 Win | 22 | 3 | Recreation |
| Winchester | XPR | Rifle | 325 WSM | 24 | 3 | Recreation |
| Winchester | XPR | Rifle | 338 Win Mag | 26 | 3 | Recreation |
| Winchester | XPR | Rifle | 6.5 Creedmoor | 22 | 3 | Recreation |
| Winchester | XPR | Rifle | 7mm Rem Mag | 26 | 3 | Recreation |
| Winchester | XPR | Rifle | 7mm-08 Rem | 22 | 3 | Recreation |
| Winchester | XPR Compact | Rifle | 243 Win | 20 | 3 | Recreation |
| Winchester | XPR Compact | Rifle | 270 WSM | 22 | 3 | Recreation |
| Winchester | XPR Compact | Rifle | 300 WSM | 22 | 3 | Recreation |
| Winchester | XPR Compact | Rifle | 308 Win | 20 | 3 | Recreation |
| Winchester | XPR Compact | Rifle | 325 WSM | 22 | 3 | Recreation |
| Winchester | XPR Compact | Rifle | 6.5 Creedmoor | 20 | 3 | Recreation |
| Winchester | XPR Compact | Rifle | 7mm-08 Rem | 20 | 3 | Recreation |
| Winchester | XPR Hunter Break-Up Country | Rifle | 243 Win | 22 | 3 | Recreation |
| Winchester | XPR Hunter Break-Up Country | Rifle | 270 Win | 24 | 3 | Recreation |
| Winchester | XPR Hunter Break-Up Country | Rifle | 270 WSM | 24 | 3 | Recreation |
| Winchester | XPR Hunter Break-Up Country | Rifle | 300 Win Mag | 26 | 3 | Recreation |
| Winchester | XPR Hunter Break-Up Country | Rifle | 300 WSM | 24 | 3 | Recreation |
| Winchester | XPR Hunter Break-Up Country | Rifle | 30-06 Sprg | 24 | 3 | Recreation |
| Winchester | XPR Hunter Break-Up Country | Rifle | 308 Win | 22 | 3 | Recreation |
| Winchester | XPR Hunter Break-Up Country | Rifle | 325 WSM | 24 | 3 | Recreation |
| Winchester | XPR Hunter Break-Up Country | Rifle | 338 Win Mag | 26 | 3 | Recreation |
| Winchester | XPR Hunter Break-Up Country | Rifle | 6.5 Creedmoor | 22 | 3 | Recreation |
| Winchester | XPR Hunter Break-Up Country | Rifle | 7mm Rem Mag | 26 | 3 | Recreation |
| Winchester | XPR Hunter Break-Up Country | Rifle | 7mm-08 Rem | 22 | 3 | Recreation |
| Winchester | XPR Hunter Compact Break-Up Country | Rifle | 243 Win | 20 | 3 | Recreation |
| Winchester | XPR Hunter Compact Break-Up Country | Rifle | 270 WSM | 22 | 3 | Recreation |
| Winchester | XPR Hunter Compact Break-Up Country | Rifle | 300 WSM | 22 | 3 | Recreation |
| Winchester | XPR Hunter Compact Break-Up Country | Rifle | 308 Win | 20 | 3 | Recreation |
| Winchester | XPR Hunter Compact Break-Up Country | Rifle | 325 WSM | 22 | 3 | Recreation |
| Winchester | XPR Hunter Compact Break-Up Country | Rifle | 6.5 Creedmoor | 20 | 3 | Recreation |
| Winchester | XPR Hunter Compact Break-Up Country | Rifle | 7mm-08 Rem | 20 | 3 | Recreation |
| Winchester | XPR Sporter | Rifle | 243 Win | 22 | 3 | Recreation |
| Winchester | XPR Sporter | Rifle | 270 Win | 24 | 3 | Recreation |
| Winchester | XPR Sporter | Rifle | 270 WSM | 24 | 3 | Recreation |
| Winchester | XPR Sporter | Rifle | 300 Win | 26 | 3 | Recreation |
| Winchester | XPR Sporter | Rifle | 300 WSM | 24 | 3 | Recreation |
| Winchester | XPR Sporter | Rifle | 30-06 Sprg | 24 | 3 | Recreation |
| Winchester | XPR Sporter | Rifle | 308 Win | 22 | 3 | Recreation |
| Winchester | XPR Sporter | Rifle | 325 WSM | 24 | 3 | Recreation |
| Winchester | XPR Sporter | Rifle | 338 Win Mag | 26 | 3 | Recreation |
| Winchester | XPR Sporter | Rifle | 6.5 Creedmoor | 22 | 3 | Recreation |
| Winchester | XPR Sporter | Rifle | 7mm Rem Mag | 26 | 3 | Recreation |
| Winchester | XPR Sporter | Rifle | 7mm-08 Rem | 22 | 3 | Recreation |
| Beretta | 690 | Shotgun | 3.00 | 49.80 | 2 | Recreation |
| Beretta | 692 | Shotgun | 3.00 | 49.80 | 2 | Recreation |
| Beretta | 695 | Shotgun | 3.00 | 50.00 | 2 | Recreation |
| Beretta | 1301 Competition | Shotgun | 3.00 | 37.80 | 5 | Recreation |
| Beretta | 1301 Tactical | Shotgun | 3.00 | 37.80 | 4 | Tactical |
| Beretta | 486 Parallelo | Shotgun | 3.00 | 45.25 | 2 | Recreation |
| Beretta | 686 Silver Pigeon I | Shotgun | 3.00 | 48.00 | 2 | Recreation |
| Beretta | 686 Ultralight | Shotgun | 2.75 | 46.00 | 2 | Recreation |
| Beretta | 687 EELL Diamond Pigeon | Shotgun | 3.00 | 47.50 | 2 | Recreation |
| Beretta | 691 (including Vittoria) | Shotgun | 3.00 | 45.50 | 2 | Recreation |
| Beretta | 693 (including Vittoria) | Shotgun | 3.00 | 48.00 | 2 | Recreation |
| Beretta | A300 Outlander | Shotgun | 3.00 | 48.50 | 3 | Recreation |
| Beretta | A350 Xtrema | Shotgun | 3.50 | 48.50 | 5 | Recreation |
| Beretta | A400 Lite | Shotgun | 3.00 | 49.25 | 4 | Recreation |
| Beretta | A400 Ultralite | Shotgun | 3.00 | 48.50 | 2 | Recreation |
| Beretta | A400 Upland | Shotgun | 3.00 | 51.00 | 3 | Recreation |
| Beretta | A400 Xcel | Shotgun | 3.00 | 50.50 | 3 | Recreation |
| Beretta | A400 Xplor Action | Shotgun | 3.00 | 48.50 | 4 | Recreation |
| Beretta | A400 Xtreme Plus | Shotgun | 3.50 | 49.00 | 4 | Recreation |
| Beretta | DT11 | Shotgun | 3.00 | 47.75 | 2 | Recreation |
| Browning | 725 Citori feather | shotgun | 3 | 28 | 2 | Recreation |
| Browning | 725 Citori feather | shotgun | 3 | 28 | 2 | Recreation |
| Browning | 725 Citori field | shotgun | 3 | 28 | 2 | Recreation |
| Browning | 725 Citori field | shotgun | 3 | 28 | 2 | Recreation |
| Browning | 725 Citori field | shotgun | 3 | 28 | 2 | Recreation |
| Browning | 725 Citori field | shotgun | 2-3/4 | 28 | 2 | Recreation |
| Browning | 725 Citori Field Grade VI-12g | shotgun | 3 | 28 | 2 | Recreation |
| Browning | 725 Citori Field Grade VI-20g | shotgun | 3 | 28 | 2 | Recreation |
| Browning | 725 Citori high rib sporting | shotgun | 3 | 32 | 2 | Recreation |
| Browning | 725 Citori high rib with adj comb | shotgun | 3 | 32 | 2 | Recreation |
| Browning | 725 Citori Pro Sporting w Pro fit adj comb | shotgun | 2-3/4 | 32 | 2 | Recreation |
| Browning | 725 Citori Pro Sporting w pro fit adj comb | shotgun | 2-3/4 | 32 | 2 | Recreation |
| Browning | 725 Citori Pro Trap with pro fit adj comb | shotgun | 2-3/4 | 32 | 2 | Recreation |
| Browning | 725 Citori Sporting | shotgun | 3 | 32 | 2 | Recreation |
| Browning | 725 Citori Sporting | shotgun | 3 | 32 | 2 | Recreation |
| Browning | 725 Citori Sporting | shotgun | 3 | 32 | 2 | Recreation |
| Browning | 725 Citori Sporting | shotgun | 2-3/4 | 32 | 2 | Recreation |
| Browning | 725 Citori Sporting Golden Clays | shotgun | 2-3/4 | 32 | 2 | Recreation |
| Browning | 725 Citori Sporting with adj comb | shotgun | 3 | 32 | 2 | Recreation |
| Browning | 725 Citori trap | shotgun | 2-3/4 | 32 | 2 | Recreation |
| Browning | 725 Citori Trap Golden Clays | shotgun | 2-3/4 | 32 | 2 | Recreation |
| Browning | 725 Citori trap with adj comb | shotgun | 2-3/4 | 32 | 2 | Recreation |
| Browning | A5 High Grade Hunter | shotgun | 3 | 28 | 1 | Recreation |
| Browning | A5 Hunter | shotgun | 3 | 30 | 1 | Recreation |
| Browning | A5 Hunter | shotgun | 3-1/2 | 30 | 1 | Recreation |
| Browning | A5 MOBUC | shotgun | 3 | 30 | 1 | Recreation |
| Browning | A5 MOBUC | shotgun | 3-1/2 | 30 | 1 | Recreation |
| Browning | A5 MOSGB | shotgun | 3 | 30 | 1 | Recreation |
| Browning | A5 MOSGB | shotgun | 3-1/2 | 30 | 1 | Recreation |
| Browning | A5 Stalker | shotgun | 3 | 30 | 1 | Recreation |
| Browning | A5 Stalker | shotgun | 3-1/2 | 30 | 1 | Recreation |
| Browning | A5 Sweet Sixteen | shotgun | 2-3/4 | 28 | 1 | Recreation |
| Browning | A5 Ultimate | shotgun | 3 | 28 | 1 | Recreation |
| Browning | A5 Wicked Wing MOSGB | shotgun | 3 | 30 | 1 | Recreation |
| Browning | A5 Wicked Wing MOSGB | shotgun | 3-1/2 | 30 | 1 | Recreation |
| Browning | BPS 10 Gauge MOBUC | shotgun | 3-1/2 | 28 | 5 | Recreation |
| Browning | BPS 10 Gauge MOSGB | shotgun | 3-1/2 | 28 | 5 | Recreation |
| Browning | BPS 10 Gauge NWTF BPS | shotgun | 3-1/2 | 24 | 5 | Recreation |
| Browning | BPS 10 Gauge Stalker | shotgun | 3-1/2 | 28 | 5 | Recreation |
| Browning | BPS Field | shotgun | 3 | 28 | 4 | Recreation |
| Browning | BPS Field | shotgun | 3 | 28 | 4 | Recreation |
| Browning | BPS Field | shotgun | 3 | 26 | 4 | Recreation |
| Browning | BPS Field | shotgun | 2-3/4 | 28 | 5 | Recreation |
| Browning | BPS Field | shotgun | 2-3/4 | 28 | 5 | Recreation |
| Browning | BPS Gold Light 10 Gauge Mobuc | shotgun | 3-1/2 | 28 | 5 | Recreation |
| Browning | BPS Micro Midas | shotgun | 3 | 26 | 4 | Recreation |
| Browning | BPS Micro Midas | shotgun | 3 | 26 | 4 | Recreation |
| Browning | BPS Micro Trap | shotgun | 2-3/4 | 28 | 5 | Recreation |
| Browning | BPS MOBUC | shotgun | 3 | 28 | 4 | Recreation |
| Browning | BPS MOBUC | shotgun | 3-1/2 | 28 | 4 | Recreation |
| Browning | BPS MOSGB | shotgun | 3 | 28 | 4 | Recreation |
| Browning | BPS MOSGB | shotgun | 3-1/2 | 28 | 4 | Recreation |
| Browning | BPS MOSGB | shotgun | 3-1/2 | 28 | 5 | Recreation |
| Browning | BPS NWTF, MOBUC | shotgun | 3 | 24 | 4 | Recreation |
| Browning | BPS NWTF, MOBUC | shotgun | 3-1/2 | 24 | 4 | Recreation |
| Browning | BPS NWTF/MOBUC | shotgun | 3-1/2 | 24 | 5 | Recreation |
| Browning | BPS Slug Hunter | shotgun | 3 | 22 | 1 | Recreation |
| Browning | BPS Slug Hunter | shotgun | 3 | 22 | 1 | Recreation |
| Browning | BPS Slug Mobuc 12 GA | shotgun | 3 | 22 | 1 | Recreation |
| Browning | BPS Slug Mobuc 20 GA | shotgun | 3 | 22 | 1 | Recreation |
| Browning | BPS Stalker | shotgun | 3 | 28 | 4 | Recreation |
| Browning | BPS Stalker | shotgun | 3-1/2 | 28 | 4 | Recreation |
| Browning | BPS Trap | shotgun | 2-3/4 | 30 | 5 | Recreation |
| Browning | BT 99 | shotgun | 2-3/4 | 34 | 1 | Recreation |
| Browning | BT 99 Golden Clays | shotgun | 2-3/4 | 34 | 1 | Recreation |
| Browning | BT 99 micro | shotgun | 2-3/4 | 32 | 1 | Recreation |
| Browning | BT 99 micro midas | shotgun | 2-3/4 | 30 | 1 | Recreation |
| Browning | BT 99 micro w adj buttplate an comb | shotgun | 2-3/4 | 32 | 1 | Recreation |
| Browning | BT 99 Plus | shotgun | 2-3/4 | 34 | 1 | Recreation |
| Browning | BT 99 with adj buttplate & comb | shotgun | 2-3/4 | 34 | 1 | Recreation |
| Browning | Citori CXS | shotgun | 3 | 32 | 2 | Recreation |
| Browning | Citori CXS | shotgun | 3 | 32 | 2 | Recreation |
| Browning | Citori CXS with adj comb | shotgun | 3 | 32 | 2 | Recreation |
| Browning | Citori CXT | shotgun | 3 | 32 | 2 | Recreation |
| Browning | Citori CXT with adj comb | shotgun | 3 | 32 | 2 | Recreation |
| Browning | Cynergy Classic Trap unsingle combo with adj comb | shotgun | 2-3/4 | 34 | 2 | Recreation |
| Browning | Cynergy Composite Ultimate turkey, mobuc | shotgun | 3-1/2 | 26 | 2 | Recreation |
| Browning | Cynergy CX | shotgun | 3 | 32 | 2 | Recreation |
| Browning | Cynergy CX Compposite | shotgun | 3 | 32 | 2 | Recreation |
| Browning | Cynergy CX with adj comb | shotgun | 3 | 32 | 2 | Recreation |
| Browning | Cynergy Field | shotgun | 3 | 28 | 2 | Recreation |
| Browning | Cynergy Field | shotgun | 3 | 28 | 2 | Recreation |
| Browning | Cynergy Micro Midas | shotgun | 3 | 26 | 2 | Recreation |
| Browning | Maxus All-purpose, MOBUC | shotgun | 3-1/2 | 26 | 1 | Recreation |
| Browning | Maxus Hunter | shotgun | 3 | 30 | 1 | Recreation |
| Browning | Maxus Hunter | shotgun | 3-1/2 | 30 | 1 | Recreation |
| Browning | Maxus MOSGB | shotgun | 3 | 28 | 1 | Recreation |
| Browning | Maxus MOSGB | shotgun | 3-1/2 | 28 | 1 | Recreation |
| Browning | Maxus Sporting | shotgun | 3 | 30 | 1 | Recreation |
| Browning | Maxus Sporting Carbon Fiber | shotgun | 3 | 30 | 1 | Recreation |
| Browning | Maxus Sporting Golden Clays | shotgun | 3 | 30 | 1 | Recreation |
| Browning | Maxus Stalker | shotgun | 3 | 28 | 1 | Recreation |
| Browning | Maxus Stalker | shotgun | 3-1/2 | 28 | 1 | Recreation |
| Browning | Maxus Ultimate | shotgun | 3 | 30 | 1 | Recreation |
| Browning | Maxus Wicked Wing, MOSGB | shotgun | 3 | 28 | 1 | Recreation |
| Browning | Maxus Wicked Wing, MOSGB | shotgun | 3-1/2 | 28 | 1 | Recreation |
| Browning | Silver Black Lightning | shotgun | 3 | 28 | 1 | Recreation |
| Browning | Silver Field | shotgun | 3 | 28 | 1 | Recreation |
| Browning | Silver Field | shotgun | 3 | 28 | 1 | Recreation |
| Browning | Silver Field Composite | shotgun | 3 | 28 | 1 | Recreation |
| Browning | Silver Field Composite | shotgun | 3-1/2 | 28 | 1 | Recreation |
| Browning | Silver Field Micro Midas | shotgun | 3 | 26 | 1 | Recreation |
| Browning | Silver Field Micro Midas | shotgun | 3 | 26 | 1 | Recreation |
| Browning | Silver Field, MOSGB | shotgun | 3-1/2 | 28 | 1 | Recreation |
| Browning | Silver Slug Matte | shotgun | 3 | 22 | 1 | Recreation |
| Browning | Silver Slug Mobuc | shotgun | 3 | 22 | 1 | Recreation |
| Browning | Silver Slug Mobuc | shotgun | 3 | 22 | 1 | Recreation |
| Century Arms | JW-2000 Shotgun | Shotgun | 3.00 | 37.00 | 2 | Recreation |
| Century Arms | PW87 Shotgun | Shotgun | 3.00 | 37.75 | 5 | Recreation |
| FN America | FN P-12 | Shotgun | 3.00 | 39.00 | 5 | Tactical |
| FN America | FN SLP | Shotgun | 3.00 | 39.00 | 6 | Tactical |
| FN America | FN SLP Competition | Shotgun | 3.00 | 45.00 | 8 | Recreation |
| FN America | FN SLP MKI | Shotgun | 3.00 | 43.00 | 8 | Tactical |
| FN America | FN SLP MKI Tactical | Shotgun | 3.00 | 43.00 | 8 | Tactical |
| FN America | FN SLP Tactical | Shotgun | 3.00 | 39.00 | 6 | Tactical |
| Henry USA | Single Shot Shotgun | Shotgun | 12 | 43.5 | 1 | Recreation |
| Henry USA | Single Shot Shotgun | Shotgun | 20 | 41.5 | 1 | Recreation |
| Henry USA | Single Shot Shotgun | Shotgun | .410 Bore | 41.5 | 1 | Recreation |
| Kel-Tec | KSG | Shotgun | 3.00 | 26.10 | 12 | Tactical |
| Kel-Tec | KSG Tactical | Shotgun | 3.00 | 21.50 | 8 | Tactical |
| Kel-Tec | KSG-25 | Shotgun | 3.00 | 38.00 | 20 | Tactical |
| Kel-Tec | KSG-NR | Shotgun | 3.00 | 26.10 | 8 | Tactical |
| Mossberg | 500 | Shotgun |  | 48.50 | 6 | Recreation |
| Mossberg | 590 | Shotgun |  | 41.00 | 9 | Recreation |
| Mossberg | 500 Tactical - 6 Shot - with Pistol Grip | Shotgun |  | 28.12 | 6 | Tactical |
| Mossberg | 500 Tactical - 8 Shot - with Pistol Grip | Shotgun |  | 30.25 | 8 | Tactical |
| Mossberg | 500 Tactical - Chainsaw - with Pistol Grip | Shotgun |  | 28.12 | 6 | Tactical |
| Mossberg | 500 Tactical - JIC | Shotgun |  | 28.12 | 6 | Tactical |
| Mossberg | 500 Tactical - JIC Flex - with Pistol Grip | Shotgun |  | 28.12 | 6 | Tactical |
| Mossberg | 535 ATS | Shotgun |  | 48.25 | 6 | Recreation |
| Mossberg | 590 A1 | Shotgun |  | 41.00 | 9 | Self-Defense |
| Mossberg | 590 A1 Class III | Shotgun |  | 33.75 | 6 | Self-Defense |
| Mossberg | 590 Nightstick | Shotgun |  | 26.37 | 6 | Self-Defense |
| Mossberg | 590 Shockwave | Shotgun |  | 26.37 | 6 | Self-Defense |
| Mossberg | 590 Shockwave SPX | Shotgun |  | 26.37 | 6 | Self-Defense |
| Mossberg | 590 Tactical with Pistol Grip | Shotgun |  | 32.50 | 9 | Tactical |
| Mossberg | 590M Mag Fed | Shotgun |  | 39.50 | 11 | Tactical |
| Mossberg | 590M Shockwave Mag-Fed | Shotgun |  | 27.50 | 11 | Self-Defense |
| Mossberg | 835 Ulti-Mag | Shotgun |  | 48.75 | 6 | Recreation |
| Mossberg | 930 Hunting (all other) | Shotgun |  | 48.50 | 5 | Recreation |
| Mossberg | 930 JM Pro-Series | Shotgun |  | 44.50 | 10 | Recreation |
| Mossberg | 930 Pro-Series | Shotgun |  | 49.00 | 5 | Recreation |
| Mossberg | 930 Slugster | Shotgun |  | 44.50 | 5 | Recreation |
| Mossberg | 930 Snow Goose | Shotgun |  | 52.50 | 13 | Recreation |
| Mossberg | 930 Tactical | Shotgun |  | 39.00 | 8 | Tactical |
| Mossberg | 935 Magnum | Shotgun |  | 49.00 | 5 | Recreation |
| Mossberg | 935 Magnum Pro-Series | Shotgun |  | 49.00 | 5 | Recreation |
| Mossberg | Flex 500 Tactical with Pistol Grip | Shotgun |  | 31.00 | 6 | Tactical |
| Mossberg | Maverick 88 | Shotgun |  | 47.75 | 8 | Recreation |
| Mossberg | Maverick HS12 | Shotgun |  | 35.25 | 2 | Recreation |
| Mossberg | Maverick Hunter | Shotgun |  | 46.00 | 2 | Recreation |
| Mossberg | Mossberg International SA-20 | Shotgun |  | 48.50 | 5 | Recreation |
| Mossberg | Mossberg International SA-28 | Shotgun |  | 46.50 | 5 | Recreation |
| Mossberg | Mossberg International Silver Reserve II | Shotgun |  | 49.50 | 2 | Recreation |
| Remington | Model 1100 Classic Trap | Shotgun | 2.75 | 50.50 | 4 | Recreation |
| Remington | Model 1100 Competition Synthetic | Shotgun | 2.75 | 50.50 | 4 | Recreation |
| Remington | Model 1100 Sporting | Shotgun | 3.00 | 49.00 | 4 | Recreation |
| Remington | Model 11-87 Sportsman | Shotgun | 3.00 | 48.50 | 4 | Recreation |
| Remington | Model 870 DM | Shotgun | 3.00 | 38.50 | 6 | Recreation |
| Remington | Model 870 DM Tac-14 | Shotgun | 3.00 | 26.30 | 6 | Tactical |
| Remington | Model 870 Express | Shotgun | 3.50 | 48.50 | 4 | Recreation |
| Remington | Model 870 Express Tactical or Home Defense | Shotgun | 3.00 | 40.50 | 6 | Tactical |
| Remington | Model 870 Marine Magnum | Shotgun | 3.50 | 38.50 | 6 | Recreation |
| Remington | Model 870 SPS Super Mag Turkey/Predator | Shotgun | 3.50 | 40.50 | 4 | Recreation |
| Remington | Model 870 SPS Super Slug | Shotgun | 3.50 | 47.00 | 4 | Recreation |
| Remington | Model 870 Tac-14 | Shotgun | 3.00 | 26.30 | 4 | Tactical |
| Remington | Model 870 Tac-14 Arm Brace | Shotgun | 3.00 | 33.50 | 5 | Tactical |
| Remington | Model 870 Tac-14 Hardwood | Shotgun | 3.00 | 26.30 | 5 | Tactical |
| Remington | Model 870 Tac14 Marine Magnum | Shotgun | 3.00 | 26.30 | 4 | Tactical |
| Remington | Model 870 Wingmaster | Shotgun | 3.00 | 50.50 | 4 | Recreation |
| Remington | V3 Field Sport | Shotgun | 3.00 | 49.00 | 3 | Recreation |
| Remington | Versa Max Black Synthetic | Shotgun | 3.50 | 50.00 | 3 | Recreation |
| Remington | Versa Max Competition Tactical | Shotgun | 2.75 | 44.00 | 10 | Recreation |
| Remington | Versa Max Tactical | Shotgun | 3.00 | 44.00 | 8 | Tactical |
| Remington | Versa Max Waterfowl | Shotgun | 3.50 | 50.00 | 3 | Recreation |
| Savage | 212 SLUG GUN | Shotgun |  | 43.2 | 2 | Recreation |
| Savage | 220 SLUG GUN/ 220 Slug Gun SS Camo | Shotgun |  | 42.75-43.3 | 2 | Recreation |
| Savage | 301 SINGLE SHOT | Shotgun | 3 |  | 1 | Recreation |
| Savage | 320 SECURITY | Shotgun | 3" |  | 5 | Recreation |
| Savage | FOX A-GRADE | Shotgun | 3 |  | 2 | Recreation |
| Savage | STEVENS 555 | Shotgun | 2.75-3" |  | 2 | Recreation |
| Winchester | Model 101 Field | Shotgun | 3 | 26 | 2 | Recreation |
| Winchester | Model 101 Field | Shotgun | 3 | 28 | 2 | Recreation |
| Winchester | Model 101 Pigeon Trap | Shotgun | 2 3/4 | 30 | 2 | Recreation |
| Winchester | Model 101 Pigeon Trap | Shotgun | 2 3/4 | 32 | 2 | Recreation |
| Winchester | Model 101 Pigeon Trap (Adjustable comb) | Shotgun | 2 3/4 | 30 | 2 | Recreation |
| Winchester | Model 101 Pigeon Trap (Adjustable comb) | Shotgun | 2 3/4 | 32 | 2 | Recreation |
| Winchester | Model 101 Sporting | Shotgun | 2 3/4 | 28 | 2 | Recreation |
| Winchester | Model 101 Sporting | Shotgun | 2 3/4 | 30 | 2 | Recreation |
| Winchester | Model 101 Sporting | Shotgun | 2 3/4 | 32 | 2 | Recreation |
| Winchester | SX3 Black Shadow | Shotgun | 3 | 24 | 4 | Recreation |
| Winchester | SX3 Black Shadow | Shotgun | 3 | 26 | 4 | Recreation |
| Winchester | SX3 Black Shadow | Shotgun | 3 | 28 | 4 | Recreation |
| Winchester | SX3 Cantilever Buck | Shotgun | 3 | 22 | 4 | Recreation |
| Winchester | SX3 Composite Sporting | Shotgun | 2 3/4 | 28 | 4 | Recreation |
| Winchester | SX3 Composite Sporting | Shotgun | 2 3/4 | 30 | 4 | Recreation |
| Winchester | SX3 Composite Sporting | Shotgun | 2 3/4 | 32 | 4 | Recreation |
| Winchester | SX3 Composite Sporting Carbon Fiber | Shotgun | 2 3/4 | 28 | 4 | Recreation |
| Winchester | SX3 Composite Sporting Carbon Fiber | Shotgun | 2 3/4 | 30 | 4 | Recreation |
| Winchester | SX3 Composite Sporting Carbon Fiber | Shotgun | 2 3/4 | 32 | 4 | Recreation |
| Winchester | SX3 Field | Shotgun | 3 | 26 | 4 | Recreation |
| Winchester | SX3 Field | Shotgun | 3 | 28 | 4 | Recreation |
| Winchester | SX3 Field Compact | Shotgun | 3 | 24 | 4 | Recreation |
| Winchester | SX3 Field Compact | Shotgun | 3 | 26 | 4 | Recreation |
| Winchester | SX3 Field Compact | Shotgun | 3 | 28 | 4 | Recreation |
| Winchester | SX3 Long Beard | Shotgun | 3 | 24 | 4 | Recreation |
| Winchester | SX3 Long Beard | Shotgun | 3 1/2 | 24 | 4 | Recreation |
| Winchester | SX3 NWTF Cantilever Turkey | Shotgun | 3 | 24 | 4 | Recreation |
| Winchester | SX3 Ultimate Sporting Adjustable | Shotgun | 2 3/4 | 28 | 4 | Recreation |
| Winchester | SX3 Ultimate Sporting Adjustable | Shotgun | 2 3/4 | 30 | 4 | Recreation |
| Winchester | SX3 Ultimate Sporting Adjustable | Shotgun | 2 3/4 | 32 | 4 | Recreation |
| Winchester | SX3 Waterfowl Hunter | Shotgun | 3 | 26 | 4 | Recreation |
| Winchester | SX3 Waterfowl Hunter | Shotgun | 3 | 28 | 4 | Recreation |
| Winchester | SX4 | Shotgun | 3 | 28 | 4 | Recreation |
| Winchester | SX4 | Shotgun | 3 | 28 | 4 | Recreation |
| Winchester | SX4 | Shotgun | 3 1/2 | 26 | 4 | Recreation |
| Winchester | SX4 | Shotgun | 3 1/2 | 28 | 4 | Recreation |
| Winchester | SX4 Catilever Buck | Shotgun | 3 | 22 | 4 | Recreation |
| Winchester | SX4 Field | Shotgun | 3 | 26 | 4 | Recreation |
| Winchester | SX4 Field | Shotgun | 3 | 28 | 4 | Recreation |
| Winchester | SX4 Field Compact | Shotgun | 3 | 24 | 4 | Recreation |
| Winchester | SX4 Field Compact | Shotgun | 3 | 26 | 4 | Recreation |
| Winchester | SX4 Field Compact | Shotgun | 3 | 28 | 4 | Recreation |
| Winchester | SX4 NWTF Cantilever Turkey | Shotgun | 3 1/2 | 24 | 4 | Recreation |
| Winchester | SX4 Universal Hunter | Shotgun | 3 1/2 | 24 | 4 | Recreation |
| Winchester | SX4 Universal Hunter | Shotgun | 3 1/2 | 26 | 4 | Recreation |
| Winchester | SX4 Universal Hunter | Shotgun | 3 1/2 | 28 | 4 | Recreation |
| Winchester | SX4 Waterfowl Hunter | Shotgun | 3 | 28 | 4 | Recreation |
| Winchester | SX4 Waterfowl Hunter | Shotgun | 3 | 28 | 4 | Recreation |
| Winchester | SX4 Waterfowl Hunter | Shotgun | 3 1/2 | 26 | 4 | Recreation |
| Winchester | SX4 Waterfowl Hunter | Shotgun | 3 1/2 | 28 | 4 | Recreation |
| Winchester | SXP Black Shadow | Shotgun | 3 | 24 | 4 | Recreation |
| Winchester | SXP Black Shadow | Shotgun | 3 | 26 | 4 | Recreation |
| Winchester | SXP Black Shadow | Shotgun | 3 | 28 | 4 | Recreation |
| Winchester | SXP Black Shadow | Shotgun | 3 | 24 | 5 | Recreation |
| Winchester | SXP Black Shadow | Shotgun | 3 | 26 | 5 | Recreation |
| Winchester | SXP Black Shadow | Shotgun | 3 | 28 | 5 | Recreation |
| Winchester | SXP Black Shadow | Shotgun | 3 1/2 | 24 | 4 | Recreation |
| Winchester | SXP Black Shadow | Shotgun | 3 1/2 | 26 | 4 | Recreation |
| Winchester | SXP Black Shadow | Shotgun | 3 1/2 | 28 | 4 | Recreation |
| Winchester | SXP Black Shadow Deer | Shotgun | 3 | 22 | 4 | Recreation |
| Winchester | SXP Black Shadow Deer | Shotgun | 3 | 22 | 5 | Recreation |
| Winchester | SXP Extreme Deer | Shotgun | 3 | 22 | 4 | Recreation |
| Winchester | SXP Extreme Hunter | Shotgun | 3 | 22 | 4 | Recreation |
| Winchester | SXP Field | Shotgun | 3 | 26 | 4 | Recreation |
| Winchester | SXP Field | Shotgun | 3 | 28 | 4 | Recreation |
| Winchester | SXP Field | Shotgun | 3 | 26 | 5 | Recreation |
| Winchester | SXP Field | Shotgun | 3 | 28 | 5 | Recreation |
| Winchester | SXP Field Compact | Shotgun | 3 | 24 | 4 | Recreation |
| Winchester | SXP Field Compact | Shotgun | 3 | 26 | 4 | Recreation |
| Winchester | SXP Field Compact | Shotgun | 3 | 28 | 4 | Recreation |
| Winchester | SXP Field Compact | Shotgun | 3 | 24 | 5 | Recreation |
| Winchester | SXP Field Compact | Shotgun | 3 | 26 | 5 | Recreation |
| Winchester | SXP Field Compact | Shotgun | 3 | 28 | 5 | Recreation |
| Winchester | SXP Long Beard Mossy Oak Break-up Country | Shotgun | 3 | 24 | 4 | Recreation |
| Winchester | SXP Long Beard Mossy Oak Break-up Country | Shotgun | 3 | 24 | 5 | Recreation |
| Winchester | SXP Long Beard Mossy Oak Break-up Country | Shotgun | 3 1/2 | 24 | 4 | Recreation |
| Winchester | SXP Long Beard Mossy Oak Obsession | Shotgun | 3 | 24 | 4 | Recreation |
| Winchester | SXP Long Beard Mossy Oak Obsession | Shotgun | 3 | 24 | 5 | Recreation |
| Winchester | SXP Long Beard Mossy Oak Obsession | Shotgun | 3 1/2 | 24 | 4 | Recreation |
| Winchester | SXP Shadow Defender | Shotgun | 3 | 18 | 5 | Self Defense |
| Winchester | SXP Shadow Defender | Shotgun | 3 | 18 | 5 | Self Defense |
| Winchester | SXP Shadow Marine Defender | Shotgun | 3 | 18 | 5 | Self Defense |
| Winchester | SXP Shadow Marine Defender | Shotgun | 3 | 24 | 5 | Self Defense |
| Winchester | SXP Trap | Shotgun | 3 | 30 | 4 | Recreation |
| Winchester | SXP Trap | Shotgun | 3 | 32 | 4 | Recreation |
| Winchester | SXP Trap Compact | Shotgun | 3 | 28 | 4 | Recreation |
| Winchester | SXP Trap Compact | Shotgun | 3 | 30 | 4 | Recreation |
| Winchester | SXP Turkey | Shotgun | 3 | 24 | 5 | Recreation |
| Winchester | SXP Turkey | Shotgun | 3 1/2 | 24 | 4 | Recreation |
| Winchester | SXP Turkey Hunter Moss oak Obsession | Shotgun | 3 | 24 | 5 | Recreation |
| Winchester | SXP Turkey Hunter Moss oak Obsession | Shotgun | 3 1/2 | 24 | 4 | Recreation |
| Winchester | SXP Turkey Hunter Mossy Oak Break-up Country | Shotgun | 3 | 24 | 5 | Recreation |
| Winchester | SXP Turkey Hunter Mossy Oak Break-up Country | Shotgun | 3 1/2 | 24 | 4 | Recreation |
| Winchester | SXP Universal Hunter | Shotgun | 3 | 24 | 4 | Recreation |
| Winchester | SXP Universal Hunter | Shotgun | 3 | 26 | 4 | Recreation |
| Winchester | SXP Universal Hunter | Shotgun | 3 | 28 | 4 | Recreation |
| Winchester | SXP Universal Hunter | Shotgun | 3 | 24 | 5 | Recreation |
| Winchester | SXP Universal Hunter | Shotgun | 3 | 26 | 5 | Recreation |
| Winchester | SXP Universal Hunter | Shotgun | 3 | 28 | 5 | Recreation |
| Winchester | SXP Universal Hunter | Shotgun | 3 1/2 | 24 | 4 | Recreation |
| Winchester | SXP Universal Hunter | Shotgun | 3 1/2 | 26 | 4 | Recreation |
| Winchester | SXP Universal Hunter | Shotgun | 3 1/2 | 28 | 4 | Recreation |
| Winchester | SXP Waterfowl Hunter | Shotgun | 3 | 26 | 4 | Recreation |
| Winchester | SXP Waterfowl Hunter | Shotgun | 3 | 28 | 4 | Recreation |
| Winchester | SXP Waterfowl Hunter | Shotgun | 3 | 26 | 5 | Recreation |
| Winchester | SXP Waterfowl Hunter | Shotgun | 3 | 28 | 5 | Recreation |
| Winchester | SXP Waterfowl Hunter | Shotgun | 3 1/2 | 26 | 4 | Recreation |
| Winchester | SXP Waterfowl Hunter | Shotgun | 3 1/2 | 28 | 4 | Recreation |
